# Synthesis of Fucose Derivatives with Thiol Motifs towards Suicide Inhibition of *Helicobacter pylori*

**DOI:** 10.3390/molecules25184281

**Published:** 2020-09-18

**Authors:** Mark Reihill, Lorenzo Guazzelli, Han Remaut, Stefan Oscarson

**Affiliations:** 1 Centre for Synthesis and Chemical Biology, University College Dublin, Belfield, Dublin 4, Ireland; mark.reihill@ucdconnect.ie; 2 Department of Pharmacy, University of Pisa, Via Bonanno 33, 56126 Pisa, Italy; lorenzo.guazzelli@unipi.it; 3 VIB-VUB Center for Structural Biology, Pleinlaan 2, Building E, 1050 Brussel, Belgium; han.remaut@vub.be

**Keywords:** *H. pylori*, Lewis b, fucose, suicide inhibitors, BabA, lectins, thiols

## Abstract

The syntheses of six thiol-exhibiting monosaccharides towards suicide inhibition of *Helicobacter pylori* are reported. Blood group Antigen Binding Adhesin (BabA), a bacterial membrane-bound lectin, binds to human ABO and Lewis b blood group structures displayed on the surface of host epithelial cells. Crystal structures of the carbohydrate-recognition domain revealed a conserved disulfide bonded loop that anchors a critical fucose residue in these blood group structures. Disruption of this loop by *N*-acetylcysteine results in reduced BabA-mediated adherence to human gastric tissue sections and attenuated virulence in Lewis b-expressing transgenic mice. With a view of creating specific inhibitors of the lectin, we designed and successfully synthesised six fucose-derived compounds with thiol motifs to engage in a thiol-disulfide exchange with this disulfide bond of BabA and form a glycan-lectin disulfide linkage. Branching and extending the fucose backbone with 2- and 3-carbon thiol motifs delivered a range of candidates to be tested for biological activity against BabA.

## 1. Introduction

*Helicobacter pylori*, a Gram-negative helical-shaped bacterium, infects the stomach of almost 50% of the population [1,2,3]. Though most infections tend to be asymptomatic, in up to 10% of carriers, *H. pylori* is associated with the development of diseases such as gastritis, peptic ulcers and, in extreme cases, stomach cancers [4,5,6,7,8]. The bacterium survives the harsh acidic conditions of the stomach due to generation of ammonia and bicarbonate from epithelial urea and buries itself into the gastric mucosa, where the pH is relatively neutral [9,10]. Current treatments for infections have moved towards combination therapies, which involve the use of a proton-pump inhibitor and at least two antibiotics due to increasing levels of antibiotic resistance [11,12,13,14,15].

Binding of *H. pylori* to host epithelial cells is multivalent, but a prominent interaction is that of the blood group Antigen Binding Adhesin BabA to human ABO and Lewis b blood group structures of the lacto series displayed on the surface of the cells [16,17]. Transfer of effector proteins like VacA and CagA is facilitated through this binding, which increases the virulence of BabA-positive strains. Studies have shown that strains which are triple-positive for BabA, VacA and CagA are significantly more likely to cause gastric diseases [18].

**Figure 1:**
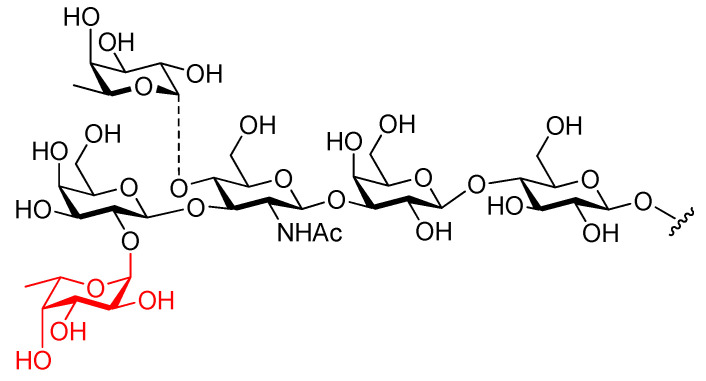
Highlighted in red is the fucose residue bound in CL2 of BabA [19].

*Moonens* et al. reported a series of crystal structures of the carbohydrate-recognition domain of BabA bound to a Lewis b hexasaccharide synthesised in our group [19,20,21]. Structures of BabA originating from different clinical isolates showed a highly polymorphous binding site, with the exception of a fucose-binding subsite. A particular paratope, Cysteine-clasped Loop 2 (CL2), was observed to be strongly conserved and served as a critical anchoring point for the binding of the α(1→2) linked fucose residue in the blood group antigen structures (Figure 1).

CL2 was reported by Moonens et al. to contain a disulfide bond between Cys189 and Cys197, constraining the loop into the conformation in which the α(1→2) fucose residue is bound [19]. Interestingly, they outlined that BabA-mediated adherence of *H. pylori* to human gastric tissue sections was prevented through treatment with the redox reagent *N*-acetylcysteine (NAC). Furthermore, Le^b^-expressing transgenic mice infected with *H. pylori* showed reduced bacterial titers and neutrophil infiltration when their drinking water was dosed with NAC. The authors attributed these observations to disruption of the Cys189-Cys197 disulfide bond, providing optimism for this to be a target of future *H. pylori* inhibitors. However, due to the lack in binding specificity for the BabA carbohydrate binding site, high doses of NAC are required to reduce the CL2 disulfide and inhibit BabA adherence [19].

**Scheme 1:**
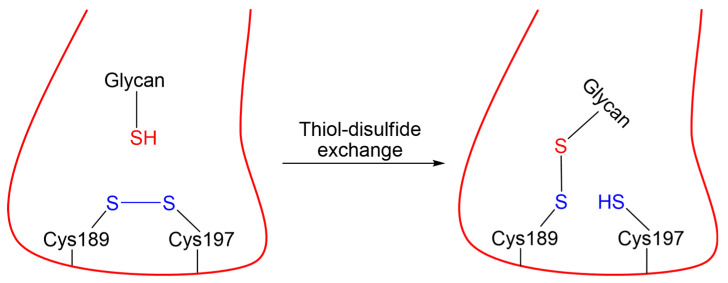
Representation of possible thiol-disulfide exchange for inhibition of BabA.

Based on the proof-of-principle of NAC in diminishing *H. pylori* binding, we hypothesised that we could design more specific redox-active inhibitors of BabA by mimicking the natural fucosyl epitope of CL2 and introducing thiol moieties onto fucose-derived backbones. These compounds could then potentially perform a thiol-disulfide exchange with the Cys189-Cys197 disulfide bond and act as suicide inhibitors of BabA (Scheme 1).

**Figure 2:**
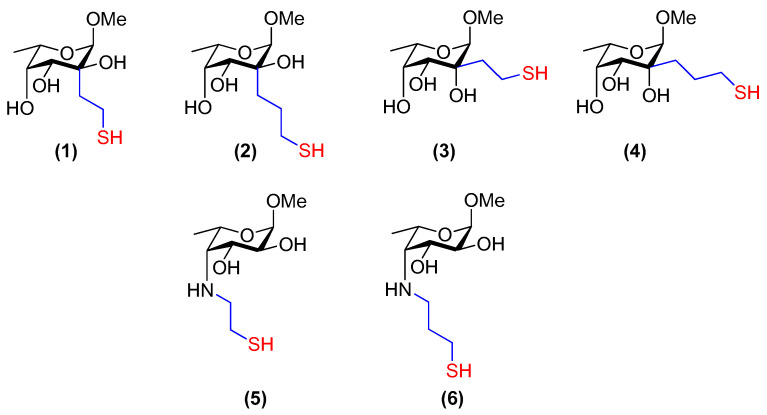
Target compounds **1**–**6**.

Based on rational design, we targeted the syntheses of compounds **1**–**6** (Figure 2). The 2- and 3-carbon spacers were desired to enable reaching towards the disulfide bond in CL2. We decided to synthesise α-*O*-methyl glycosides to stay consistent with the natural α-linkage, as shown previously in Figure 1.

**Scheme 2:**
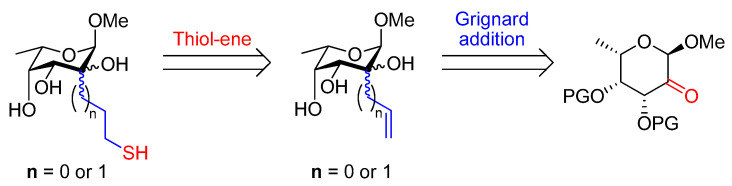
Retrosynthetic pathway for targets **1**–**4** (PG = protecting group).

Targets **1** and **2** both exhibited axial thiol motifs from the 2-position, with the structures being branched to attempt to maintain H-bonding interactions with the 2-OH. Inspired by the work of Cleator et al., we envisioned proceeding via oxidation of the 2-position followed by addition of an alkenyl Grignard reagent (Scheme 2) [22]. This would then provide us with a handle to perform thiol-ene reactions to introduce thiol groups. Since we predicted that the Grignard reaction would generate a mixture of diastereomers, we chose to also synthesise equatorial thiol compounds **3** and **4**.

**Scheme 3:**
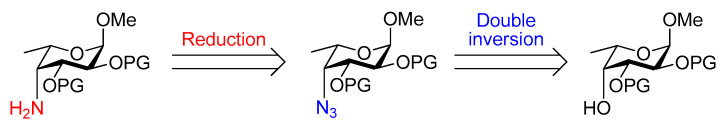
Retrosynthetic pathway of a key intermediate towards targets **5** and **6** (PG = protecting group).

Given that the 4-substituent of L-fucose is natively axial, we aimed to take advantage of this by extending the 4-position in targets **5** and **6**, rather than branching at another location. Introduction of a secondary amine at this position was also desired to aid in maintaining H-bonding interactions within CL2. We decided to follow a similar pathway to Rabuka et al. and perform a double inversion at the 4-position to introduce an axial azido group [23]. Reduction would then yield an intermediate containing a primary amine (Scheme 3), with *N*-alkylation allowing us to introduce 2- and 3-carbon spacers towards **5** and **6**.

**Scheme 4:**
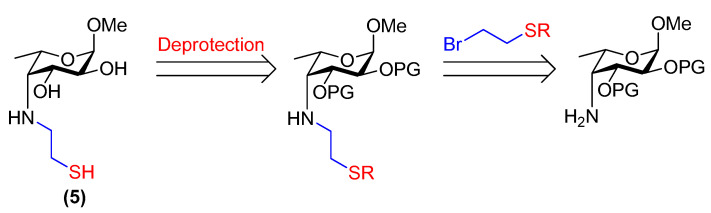
Retrosynthesis of target **5**.

For target **5**, we proposed introducing the 2-carbon motif through *N*-alkylation with an alkyl bromide containing a protected thiol (Scheme 4). Deprotection would then yield the final structure.

**Scheme 5:**
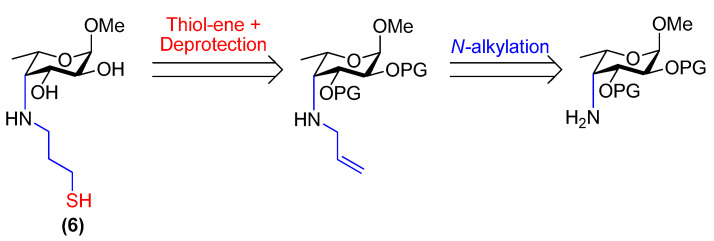
Retrosynthetic pathway for compound **6**.

Towards target **6**, we envisioned installing an *N*-allyl group and then performing a thiol-ene reaction to introduce a protected thiol. Removal of the protecting groups would then afford the target compound (Scheme 5).

## 2. Results and Discussion

### 2.1. Branched Targets ***1***–***4***

**Scheme 6:**
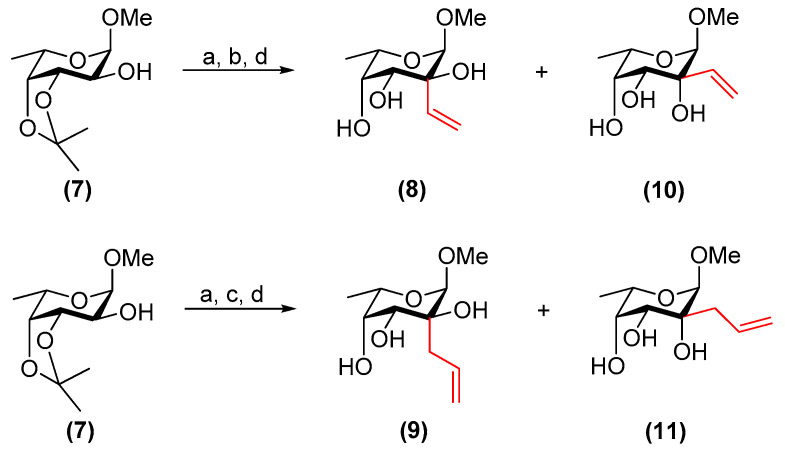
Syntheses of alkenyl compounds **8**–**11**. (**a**) NMO, TPAP (cat.), 4 Å molecular sieves, CH_2_Cl_2_, rt, 1.5–2 h; (**b**) 1 M vinylmagnesium bromide/THF, toluene, 0 °C, 10 min; (**c**) 1 M allylmagnesium bromide/Et_2_O, toluene, 0 °C, 10 min; (**d**) Dowex^®^ (H^+^) resin, MeOH, rt, 16 h–48 h. Yields over 3 steps: (**8**) = 14%, (**10**) = 50% (d.r. = 1:3.6); (**9**) = 34%; (**11**) = 21% (d.r. = 1.6:1).

In a telescoped procedure (Scheme 6) beginning from 3,4-isopropylidene protected compound **7**, the 2-OH was oxidised to the corresponding ulose with *N*-methylmorpholine *N*-oxide (NMO) and catalytic tetrapropylammonium perruthenate (TPAP) [24,25,26,27]. This was then used in a Grignard reaction with vinylmagnesium bromide, followed by acidic cleavage of the isopropylidene group, yielding triols **8** (14%) and **10** (50%) over 3 steps. Compounds **9** (34%) and **11** (21%) were prepared in a similar fashion over 3 steps using allylmagnesium bromide as the Grignard reagent. Each diastereomer was separated by flash chromatography and the stereochemistry was assigned through analysis of NOESY spectra.

**Scheme 7:**
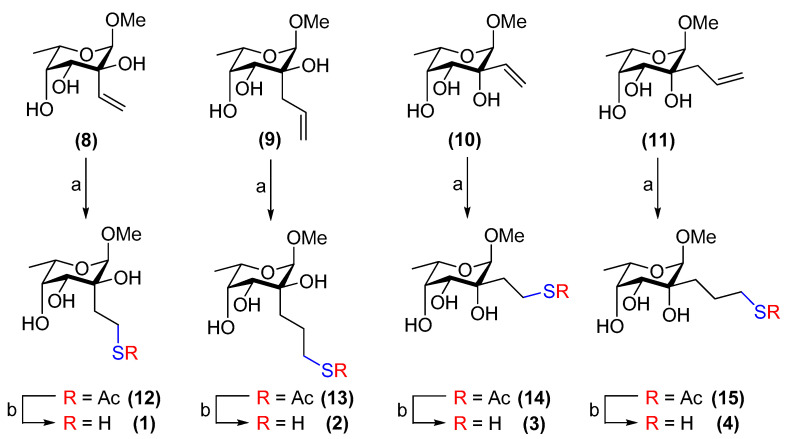
Thiol-ene reactions and deacetylation towards targets **1**–**4**. (**a**) AcSH, AIBN, 1,4-dioxane, 75 °C, 3 h, (**12**) = 73%; (**13**) = 84%; (**14**) = 85%; (**15**) = 84%; (**b**) 0.4 M NaOMe/MeOH, MeOH, rt, 5 min, (**1**) = 92%; (**2**) = 92%; (**3**) = 92%; (**4**) = 95%.

Compounds **8**, **9**, **10** and **11** underwent AIBN-initiated thiol-ene reactions with thioacetic acid to furnish corresponding thioacetate-containing compounds **12**, **13**, **14** and **15**, respectively, in yields between 73% and 85% (Scheme 7). Deacetylation with freshly prepared, de-gassed NaOMe/MeOH solution in dry, de-gassed MeOH yielded targets **1**–**4** in yields ranging from 92%–95%, with little to no evidence of unwanted disulfide side-products. Monitoring of the deacetylations proved to be difficult since the thioacetates and their corresponding free thiols had the same R*_f_* values by TLC. However, the reactions were observed to be complete in a short period of time (5–15 min) with longer reaction times (16–18 h) leading to some disulfide formation.

### 2.2. Extended Targets ***5*** and ***6***

**Scheme 8:**
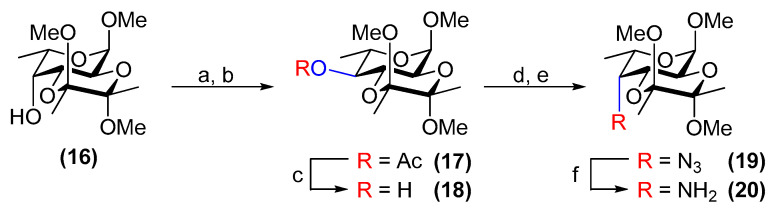
Synthesis of intermediate **20** via double inversion. (**a**) Tf_2_O, CH_2_Cl_2_/Py, −40 °C, 4 h; (**b**) Bu_4_NOAc, toluene, rt, 14 h, 68% over 2 steps; (**c**) NaOMe (cat.), MeOH, rt, 19 h, 94%; (**d**) Tf_2_O, CH_2_Cl_2_/Py, −20 °C, 4 h; (**e**) NaN_3_, DMF, 60 °C, 17 h, 70% over 2 steps; (**f**) H_2_ (5 bar), Pd/C (cat.), EtOH, rt, 21 h, 91%.

Commencing with 2,3-butanediacetal (BDA)-protected compound **16**, the 4-OH was triflated with Tf_2_O in CH_2_Cl_2_/Py at −40 °C and subsequently displaced with Bu_4_NOAc, furnishing equatorial 4-OAc compound **17** in a 68% yield over 2 steps [28]. The R_f_ values of the triflate and **17** were extremely similar by TLC and so mass spectrometry aided in monitoring of the displacement reaction. Deacetylation under Zemplén conditions then yielded compound **18** (94%) [29,30]. Triflation, similar to before, and S_N_2 inversion with NaN_3_ furnished **19** (70% over 2 steps). Pd-catalysed reduction of the azido-group, under a H_2_ atmosphere, generated primary amine intermediate **20** in a 91% yield (Scheme 8).

#### 2.2.1. Target **5**

**Scheme 9:**
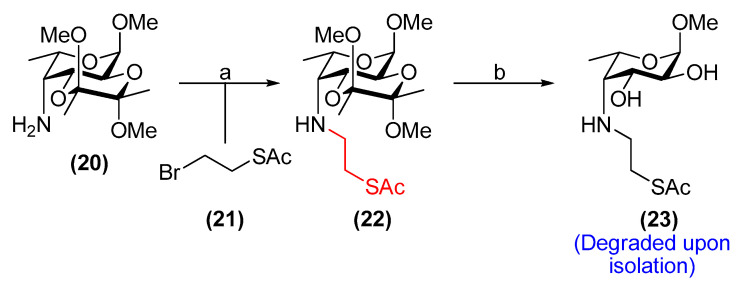
*N*-alkylation and degradation of compound **23**. (**a**) K_2_CO_3_, KI, DMF, rt, 4 days, 40%; (**b**) TFA/H_2_O (9:1, *v*/*v*), rt, 3 h, then Amberlyst^®^ A21 resin, CH_2_Cl_2_, rt, 16 h, 77%.

Towards target **5**, as detailed in Scheme 9, we opted to proceed via *N*-alkylation using alkyl bromide **21** [31]. Alkylation of the primary amine of **20**, in the presence of K_2_CO_3_ and KI, proceeded in a 40% yield to furnish compound **22**. When using these mild alkylation conditions, we typically observed little to no over-alkylation of the amine; however, we suspect the diminished yield was due to S→N acyl-transfer taking place as a side-reaction [32]. The BDA protecting group was then removed in TFA/H_2_O, yielding 77% of diol **23**. We often observed the formation of an amine-TFA salt during this reaction, but stirring with Amberlyst^®^ A21 resin afterwards removed any TFA and was therefore incorporated as part of the work-up [33].

At this point, deacetylation was attempted, but resulted in the formation of a range of products (inseparable by TLC/flash chromatography). Upon further investigation, we realised that compound **23** had degraded within 5 days. Interestingly, Paritala et al. have also reported degradation upon isolation of a product containing the same 2-carbon thioacetyl motif [34]. They reported that stability of their product could be increased by replacing the thioacetyl group with that of a thiobenzoyl, perhaps suggesting that degradation was occurring due to migration of the acetyl group. We therefore decided trial the approach of employing a benzoyl protecting group.

**Scheme 10:**
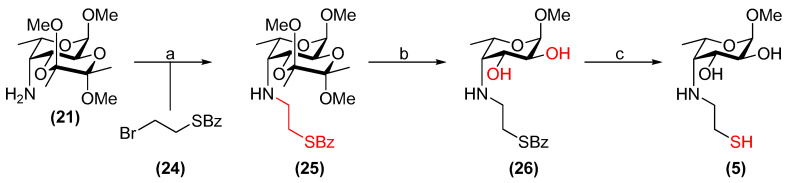
Re-designed synthesis towards target **5**. (**a**) K_2_CO_3_, KI (cat.), DMF, 80 °C, 20 h, 49%; (**b**) TFA/H_2_O (9:1, *v*/*v*), rt, 3 h, then Amberlyst^®^ A21 resin, CH_2_Cl_2_, rt, 18 h, 64%; (**c**) 1 M NaOMe/MeOH, MeOH, rt, 15 min, 58% (95% thiol, 5% disulfide).

Alkyl bromide **24** was synthesised from 1,2-dibromoethane and sodium thiobenzoate in an 86% yield [35]. Paritala et al. reported the use of microwave methodology to alkylate a primary amine in their synthetic pathway using **24** [34]. However, in our hands, we only observed S→N acyl-transfer using these conditions [32]. We then attempted *N*-alkylation at 80 °C in an oil bath in the presence of potassium iodide. In this case, we were able to isolate compound **25** in a 49% yield, with some S→N acyl-transfer still being observed by TLC (Scheme 10). The progress of the reaction if performed at room temperature, or without KI being present, was too slow to be viable.

Acid hydrolysis in TFA/H_2_O yielded diol **26** (64% yield), with no degradation being observed after isolation. De-benzoylation with freshly prepared, de-gassed 1 M NaOMe/MeOH in dry, de-gassed MeOH yielded target **5** (Scheme 10). Flash chromatography was performed through a small plug of silica gel, using de-gassed solvents, to remove methyl benzoate, the by-product from the reaction. From the mass of material collected, a 58% yield was obtained. However, according to ^1^H NMR data, up to 5% of the corresponding disulfide was isolated, giving an actual yield of 55% of thiol **5**.

#### 2.2.2. Target **6**

**Scheme 11:**
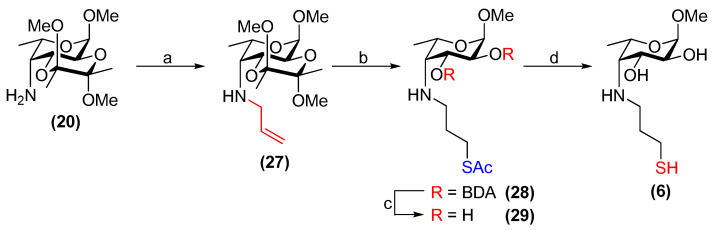
*N*-alkylation, thiol-ene and deprotection towards target **6**. (**a**) Allyl bromide, K_2_CO_3_, DMF, rt, 21 h, 68%; (**b**) 1 M Et_3_B/hexanes, AcSH, catechol, CH_2_Cl_2_, rt, 3 h, 83%; (**c**) TFA/H_2_O (9:1, *v*/*v*), rt, 4 h, then Amberlyst^®^ A21 resin, CH_2_Cl_2_, rt, 16 h, 68%; (**d**) 1 M NaOMe/MeOH, MeOH, rt, 30 min, 88% (88% thiol, 12% disulfide).

Compound **20** underwent *N*-alkylation with allyl bromide and K_2_CO_3_ in DMF to allow us to perform a thiol-ene reaction towards target **6**. Mono-alkylated product **27** was isolated in a 68% yield (Scheme 11) with what was hypothesised to be minor over-alkylation visible by TLC.

Firstly, using **27** and thioacetic acid, AIBN was trialled as an initiator in a similar procedure to what yielded compounds **12**–**15**. However, the reaction did not deliver positive results, with a complex mixture observed by TLC. Povie et al. outlined problems when performing thiol-ene reactions where an *O*-allyl or benzylic alkenyl substrate was used [36]. They reported poor yields and regioselectivity and attributed these difficulties to the stability of the radical on the carbon next to the aromatic ring or oxygen during the mechanism. This led to formation of numerous side-products due to disproportionation or recombination. Since we possessed an *N*-allyl group, we believed that our problems may have been similar to what Povie et al. observed. They reported the use of Et_3_B (initiator), catechol (repair reagent) and thioacetic acid in CH_2_Cl_2_ to mend the thiol-ene process and when these conditions were performed with **27**, the reaction proceeded smoothly to furnish compound **28** in an 83% yield (Scheme 11).

The BDA protecting group of **28** was removed via acid hydrolysis in TFA/H_2_O (68% yield), generating compound **29**, with no degradation being observed over time. Deacetylation using dry, de-gassed reagents and solvents furnished target **6** in an 88% yield based on the mass of material collected (Scheme 11). From ^1^H NMR data, up to 12% of the disulfide was observed, giving an actual yield of 77% free thiol **6**.

NMR spectra of compounds **1**–**29** are available in the Supplementary Materials.

## 3. Materials and Methods

Reactions were monitored by thin-layer chromatography (TLC) on Merck DC-Alufolien plates precoated with silica gel 60 F254 in the eluents states in parentheses (*v/v*). Visualisation was performed with UV-light (254 nm) and/or staining with 8% H2SO4/EtOH solution. All chemicals were purchased from commercial suppliers [Carbosynth Ltd. (Compton, Berkshire, UK), Fisher Scientific Ltd. (Blanchardstown, Co. Dublin, Ireland), Glycom A/S (Hørsholm, Denmark), Merck (Carrigtwohill, Co. Cork, Ireland), Sigma-Aldrich (Arklow, Co. Wicklow, Ireland), VWR (Blanchardstown, Co. Dublin, Ireland) and were used without purification. Dry solvents were obtained from a PureSolv-ENTM solvent purification system (Innovative Technology Inc., Hong Kong) or were used as purchased from Sigma-Aldrich (Arklow, Co. Wicklow, Ireland) in AcroSeal^®^ bottles. NMR spectra were recorded on Varian Inova spectrometers (Varian, Ltd., Palo Alto, CA, USA) at 25 °C. High-resolution mass spectrometry (HRMS) data were recorded on a Waters Micromass LCT LC-TOF instrument using electrospray ionisation (ESI) in positive mode. Specific rotations were recorded (Model 343) at the sodium D-line (589 nm) at 20 °C in a 1 dm cell at the stated concentration (*c* 1.0 = 10 mg/mL) on a Perkin-Elmer polarimeter (PerkinElmer Ltd., Waltham, MA, USA), except for compound **17** which was recorded on a Schmidt-Haensch UniPol L 2000 polarimeter. Deprotected sugars were lyophilised using a Christ Alpha 1-2 LDplus (SciQuip Ltd., Shropshire, UK) freeze-dryer: pressure: 0.035 mbar; ice-condenser temperature: −55 °C. Each proton and carbon belonging to the monosaccharide ring systems was numbered according to conventional guidelines [37].

### 3.1. General Procedure A (Preparation of Compounds ***12***–***15***)

Thioacetic acid (20 eq.) and azobisisobutyronitrile (1 eq.) were added to a solution of the alkene-bearing sugar (1 eq.) in dry, de-gassed 1,4-dioxane (0.5 mL) under N_2_. The reaction mixture was refluxed at 75 °C and after 3 h, the volatiles were removed in vacuo. Flash chromatography on silica gel yielded the desired product.

### 3.2. General Procedure B (Preparation of Compounds ***1***–***4***)

A freshly prepared solution of dry, de-gassed 0.4 M NaOMe/MeOH was added to a solution of the thioacetate-containing sugar in dry, de-gassed MeOH (1 mL) under N_2_ until pH 13 was reached. The mixture was stirred at room temperature for 5 min. The solution was then neutralised with Dowex^®^ (H^+^) ion-exchange resin and the resin was filtered off and washed with MeOH. The filtrate was concentrated in vacuo to furnish the product.

### 3.3. Methyl 2-C-vinyl-α-l-fucopyranoside (8) and Methyl 6-deoxy-2-C-vinyl-α-l-talopyranoside *(**10**)*

Four Å molecular sieves (150 mg), 4-methylmorpholine *N*-oxide (180 mg, 1.53 mmol), and tetrapropylammonium perruthenate (27 mg, 77 µmol) were added sequentially to a solution of compound **7** (180 mg, 0.825 mmol) in dry CH_2_Cl_2_ (7.5 mL). The resulting mixture was stirred at room temperature. After 2 h, TLC analysis (CH_2_Cl_2_/acetone, 9:1) showed the disappearance of the starting material and the formation of a less polar spot. The mixture was filtered through a Celite^®^-silica-Celite^®^ triple pad, and the filter was washed with CH_2_Cl_2_ (10 mL) and EtOAc (15 mL). The filtrate was concentrated in vacuo to afford a white solid which was used without further purification.

The crude was dissolved in dry toluene (10 mL) and the solution was cooled to 0 °C. 1 M vinylmagnesium bromide/THF (2.5 mL, 2.5 mmol) was then added dropwise. After 10 min, EtOH (1 mL) and sat. aq. NaHCO_3_ solution (10 mL) were added. The resulting mixture was extracted with EtOAc (2 × 20 mL), and the combined organic phase was dried over MgSO_4_. The solids were filtered off and the filtrate was concentrated in vacuo.

The crude residue was dissolved in MeOH (15 mL) and Dowex^®^ (H^+^) acidic ion-exchange resin (400 mg) was added. The mixture was stirred at room temperature and after 16 h, the solids were filtered off and the filtrate was concentrated in vacuo. The residue was purified by flash column chromatography on silica gel (toluene/EtOAc, 3:7→EtOAc) to afford **8** (23 mg, 14%) and **10** (85 mg, 50%) over 3 steps as white amorphous solids.

**(8)**: R*_f_* = 0.4 (EtOAc); [α]${}_{D}^{20}$ −85 (*c* 0.5, CHCl_3_); ^1^H NMR (500 MHz, CDCl_3_) δ 6.29 (dd, *J* = 17.4, 11.0 Hz, 1H, CH_2_=CH), 5.53 (dd, *J* = 17.5, 1.9 Hz, 1H, CH_2(A)_=CH), 5.32 (dd, *J* = 11.1, 1.9 Hz, 1H, CH_2(B)_=CH), 4.48 (s, 1H, H-1), 3.99 (qd, *J* = 6.6, 1.8 Hz, 1H, H-5), 3.85 (br s, 1H, H-3 or H-4), 3.81 (br s, 1H, H-3 or H-4), 3.43 (s, 3H, OCH_3_), 2.65 (s, 1H, OH), 2.57 (d, *J* = 4.6 Hz, 1H, OH), 2.30 (d, *J* = 3.3 Hz, 1H, OH), 1.32 (d, *J* = 6.6 Hz, 3H, H-6); ^13^C NMR (126 MHz, CDCl_3_) δ 136.6 (CH_2_=CH), 116.8 (CH_2_=CH), 102.7 (C-1), 74.5 (C-2), 73.3 (C-3 or C-4), 71.4 (C-3 or C-4), 65.6 (C-5), 55.7 (OCH_3_), 16.1 (C-6); HRMS (ESI) *m*/*z* calculated for C_9_H_16_O_5_ [M + Na]^+^ 227.0895, found 227.0905.

**(10)**: R*_f_* = 0.5 (EtOAc); [α]${}_{D}^{20}$−150 (*c* 1.0, CHCl_3_); ^1^H NMR (500 MHz, CDCl_3_) δ 5.91 (dd, *J* = 17.4, 10.9 Hz, 1H, CH_2_=CH), 5.48 (dd, *J* = 17.4, 1.6 Hz, 1H, CH_2(A)_=CH), 5.31 (dd, *J* = 10.9, 1.6 Hz, 1H, CH_2(B)_=CH), 4.39 (s, 1H, H-1), 4.25 (s, 1H, OH), 3.97 (q, *J* = 6.6 Hz, 1H, H-5), 3.90 (d, *J* = 6.3 Hz, 1H, OH), 3.76-3.71 (m, 2H, H-3, H-4), 3.48 (m, 1H, OH), 3.34 (s, 3H, OCH_3_), 1.32 (d, *J* = 6.6 Hz, 3H, H-6); ^13^C NMR (126 MHz, CDCl_3_) δ 137.8 (CH_2_=CH), 116.7 (CH_2_=CH), 104.4 (C-1), 75.5 (C-2), 72.1, 68.8 (C-3, C-4), 66.0 (C-5), 55.4 (OCH_3_), 16.5 (C-6); HRMS (ESI) *m*/*z* calculated for C_9_H_16_O_5_ [M + Na]^+^ 227.0895, found 227.0894.

### 3.4. Methyl 2-C-allyl-α-l-fucopyranoside (9) and Methyl 2-C-allyl-6-deoxy-α-l-talopyranoside *(**11**)*

Four Å molecular sieves (150 mg), 4-methylmorpholine *N*-oxide (252 mg, 2.15 mmol), and tetrapropylammonium perruthenate (38 mg, 0.11 mmol) were added sequentially to a solution of **7** (252 mg, 1.15 mmol) in dry CH_2_Cl_2_ (10 mL). The resulting mixture was stirred at room temperature. After 1.5 h, TLC analysis (CH_2_Cl_2_/acetone, 9:1) showed the disappearance of the starting material and the formation of a less polar spot. The mixture was filtered through a Celite^®^-silica-Celite^®^ triple pad, and the filter was washed with CH_2_Cl_2_ (10 mL) and EtOAc (15 mL). The filtrate was concentrated in vacuo to afford a white solid which was used without further purification.

The crude was dissolved in dry toluene (10 mL) and the solution was cooled to 0 °C before adding 1 M allylmagnesium bromide/Et_2_O (3.5 mL, 3.5 mmol) dropwise. After 10 min, EtOH (1 mL) and sat. aq. NaHCO_3_ solution (10 mL) were added. The resulting mixture was extracted with EtOAc (2 × 20 mL), and the organic phase was dried over MgSO_4_. The solids were filtered off and the filtrate was concentrated in vacuo.

The crude residue was dissolved in MeOH (15 mL) and Dowex^®^ (H^+^) acidic ion-exchange resin (600 mg) was added. The mixture was stirred at room temperature and after 48 h, the solids were filtered off and the filtrate was concentrated in vacuo. The residue was purified by flash chromatography on silica gel (toluene/EtOAc, 1:1→EtOAc) to afford **9** (86 mg, 34%) and **11** (54 mg, 21%) over 3 steps as white amorphous solids.

**(9)**: R*_f_* = 0.3 (EtOAc); [α]${}_{D}^{20}$−145 (*c* 1.0, CHCl_3_); ^1^H NMR (500 MHz, CDCl_3_) δ 5.93 (m, 1H, CH_2_=CH), 5.25–5.02 (m, 2H, CH_2_=CH), 4.56 (s, 1H, H-1), 3.93 (q, *J* = 6.5 Hz, 1H, H-5), 3.88 (d, *J* = 3.5 Hz, 1H, H-3 or H-4), 3.81 (br s, 1H, H-3 or H-4), 3.61–3.47 (m, 1H, OH), 3.39 (s, 3H, OCH_3_), 2.86–2.58 (m, 4H, CH-CH_2_, 2 × OH), 1.30 (d, *J* = 6.5 Hz, 3H, H-6); ^13^C NMR (151 MHz, CDCl_3_) δ 133.9 (CH_2_=CH), 118.7 (CH_2_=CH), 101.1 (C-1), 73.45 (C-2), 73.41 (C-3 or C-4), 71.4 (C-3 or C-4), 65.6 (C-5), 55.6 (OCH_3_), 36.1 (CH-CH_2_), 16.0 (C-6); HRMS (ESI) *m*/*z* calculated for C_10_H_18_O_5_ [M + Na]^+^ 241.1052, found 241.1064.

**(11)**: R*_f_* = 0.4 (EtOAc); [α]${}_{D}^{20}$−66 (*c* 1.0, CHCl_3_); ^1^H NMR (500 MHz, CDCl_3_) δ 5.87 (m, 1H, CH_2_=CH), 5.18 (s, 1H, CH_2__(A)_=CH), 5.15 (m, 1H, CH_2__(B)_=CH), 4.45 (s, 1H, H-1), 3.92 (q, *J* = 6.6 Hz, 1H, H-5), 3.68–3.52 (m, 4H, H-3, H-4, 2 × OH), 3.35 (s, 3H, OCH_3_), 2.60 (m, 1H, CH-CH_2__(A)_), 2.33 (m, 1H, CH-CH_2(B)_), 1.30 (d, *J* = 6.6 Hz, 1H, H-6); ^13^C NMR (151 MHz, CDCl_3_) δ 132.6 (CH_2_=CH), 119.4 (CH_2_=CH), 102.9 (C-1), 74.6 (C-2), 72.8 (C-3 or C-4), 69.7 (C-3 or C-4), 65.8 (C-5), 55.3 (OCH_3_), 38.8 (CH-CH_2_), 16.5 (C-6); HRMS (ESI) *m*/*z* calculated for C_10_H_18_O_5_ [M + Na]^+^ 241.1052, found 241.1054.

### 3.5. Methyl 2-C-[2-acetylthioethyl]-α-l-fucopyranoside *(**12**)*

Compound **8** (14 mg, 69 µmol) was subjected to **General Procedure A** and purified by flash chromatography on silica gel (toluene/EtOAc, 3:2→EtOAc) to afford **12** (14 mg, 73%) as a white amorphous solid. R*_f_* = 0.6 (EtOAc); [α]${}_{D}^{20}$−59 (*c* 1.0, CHCl_3_); ^1^H NMR (500 MHz, CDCl_3_) δ 4.61 (s, 1H, H-1), 3.93 (qd, *J* = 6.6, 1.9 Hz, 1H, H-5), 3.88 (br s, 1H, H-3), 3.82 (br s, 1H, H-4), 3.42 (s, 3H, OCH_3_), 3.13 (ddd, *J* = 13.3, 11.1, 5.3 Hz, 1H, CH_2__(A)_SAc), 3.02 (d, *J* = 3.8 Hz, 1H, OH), 2.90 (ddd, *J* = 13.3, 11.3, 5.4 Hz, 1H, CH_2__(B)_SAc), 2.63 (d, *J* = 1.4 Hz, 1H, OH), 2.44 (m, 1H, OH), 2.32 (s, 3H, CH_3(SAc)_), 2.20 (ddd, *J* = 14.5, 11.4, 5.3 Hz, 1H, C_(sugar)_-CH_2__(A)_), 2.06 (m, 1H, C_(sugar)_-CH_2__(B)_), 1.30 (d, *J* = 6.7 Hz, 3H, H-6); ^13^C NMR (126 MHz, CDCl_3_) δ 196.7 (C=O), 101.2 (C-1), 73.6 (C-3), 73.4 (C-2), 71.3 (C-4), 65.8 (C-5), 55.8 (OCH_3_), 31.9 (CH_2_-SAc), 30.7 (CH_3(SAc)_), 23.9 (C_(sugar)_-CH_2_), 15.9 (C-6); HRMS (ESI) *m*/*z* calculated for C_11_H_20_O_s_S [M + Na]^+^ 303.0878, found 303.0878.

### 3.6. Methyl 2-C-[3-acetylthiopropyl]-α-l-fucopyranoside *(**13**)*

Compound **9** (67 mg, 0.31 mmol) was subjected to **General Procedure A** and purified by flash chromatography on silica gel (toluene/EtOAc, 1:1→EtOAc) to afford **13** (76 mg, 84%) as a white amorphous solid. R*_f_* = 0.5 (EtOAc); [α]${}_{D}^{20}$#x2212;74 (*c* 1.0, CHCl_3_); ^1^H NMR (500 MHz, CDCl_3_) δ 4.55 (s, 1H, H-1), 3.93 (m, 1H, H-5), 3.85 (t, *J* = 3.9 Hz, 1H, H-3), 3.81 (br s, 1H, H-4), 3.56 (d, *J* = 4.1 Hz, 1H, OH), 3.40 (s, 3H, OCH_3_), 2.87 (m, 2H, CH_2_-SAc), 2.82 (m, 1H, OH), 2.72 (d, *J* = 1.3 Hz, 1H, OH), 2.32 (s, 3H, CH_3(SAc)_), 1.99–1.87 (m, 2H, -CH_2_-), 1.79 (m, 1H, C_(sugar)_-CH_2__(A)_), 1.61 (m, 1H, C_(sugar)_-CH_2__(B)_), 1.29 (d, *J* = 6.7 Hz, 3H, H-6); ^13^C NMR (126 MHz, CDCl_3_) δ 196.4 (C=O), 100.9 (C-1), 73.5 (C-2), 73.5 (C-3), 71.4 (C-4), 65.6 (C-5), 55.6 (OCH_3_), 30.7 (CH_3(SAc)_), 30.2 (-CH_2_-), 29.8 (CH_2_-SAc), 23.4 (C_(sugar)_-CH_2_), 15.9 (C-6); HRMS (ESI) *m*/*z* calculated for C_12_H_22_O_6_S [M + Na]^+^ 317.1035, found 317.1027.

### 3.7. Methyl 2-C-[2-acetylthioethyl]-6-deoxy-α-l-talopyranoside *(**14**)*

Compound **10** (50 mg, 0.25 mmol) was subjected to **General Procedure A** and purified by flash chromatography on silica gel (toluene/EtOAc, 3:2→EtOAc) to afford **14** (59 mg, 85%) as a white amorphous solid. R*_f_* = 0.6 (EtOAc); [α]${}_{D}^{20}$−72 (*c* 1.0, CHCl_3_); ^1^H NMR (500 MHz, CDCl_3_) δ 4.57 (s, 1H, H-1), 4.14 (s, 1H, OH), 3.95–3.82 (m, 2H, H-5, OH), 3.74 (d, *J* = 9.2 Hz, 1H, OH), 3.67 (dd, *J* = 6.9, 3.0 Hz, 1H, H-4), 3.55 (dd, *J* = 9.3, 3.0 Hz, 1H, H-3), 3.35 (s, 3H, OCH_3_), 3.04 (ddd, *J* = 13.3, 11.4, 4.7 Hz, 1H, CH_2__(A)_-SAc), 2.85 (ddd, *J* = 13.3, 11.2, 5.7 Hz, 1H, CH_2__(B)_-SAc), 2.33 (s, 3H, CH_3(SAc)_), 2.05 (ddd, *J* = 14.1, 11.2, 4.7 Hz, 1H, C_(sugar)_-CH_2__(A)_), 1.83 (ddd, *J* = 14.1, 11.2, 5.7 Hz, 1H, C_(sugar)_-CH_2__(B)_), 1.30 (d, *J* = 6.6 Hz, 3H, H-6); ^13^C NMR (126 MHz, CDCl_3_) δ 196.9 (C=O), 102.6 (C-1), 74.6 (C-2), 72.7 (C-4), 69.7 (C-3), 65.7 (C-5), 55.3 (OCH_3_), 34.6 (CH_2_-SAc), 30.7 (CH_3(SAc)_), 23.0 (C_(sugar)_-CH_2_), 16.5 (C-6); HRMS (ESI) *m*/*z* calculated for C_11_H_20_O_s_S [M + Na]^+^ 303.0878, found 303.0872.

### 3.8. Methyl 2-C-[3-acetylthiopropyl]-6-deoxy-α-l-talopyranoside *(**15**)*

Compound **11** (38 mg, 0.17 mmol) was subjected to **General Procedure A** and purified by flash chromatography on silica gel (toluene/EtOAc, 3:2→EtOAc) to afford **15** (43 mg, 84%) as a white amorphous solid. R*_f_* = 0.4 (toluene/EtOAc, 1:4); [α]${}_{D}^{20}$−67 (*c* 1.0, CHCl_3_); ^1^H NMR (500 MHz, CDCl_3_) δ 4.50 (s, 1H, H-1), 3.92–3.86 (m, 2H, H-5, OH), 3.79 (d, *J* = 7.1 Hz, 1H, OH), 3.65 (m, 1H, H-3 or H-4), 3.59 (d, *J* = 8.6 Hz, 1H, OH), 3.52 (br s, 1H, H-3 or H-4), 3.35 (s, 3H, OCH_3_), 2.90–2.82 (m, 2H, CH_2_-SAc), 2.33 (s, 3H, CH_3(SAc)_), 1.85 (ddd, *J* = 14.5, 7.0, 4.0 Hz, 1H, -CH_2__(A)_-), 1.75 (m, 1H, C_(sugar)_-CH_2__(A)_), 1.65–1.48 (m, 2H, -CH_2__(B)_-, C_(sugar)_-CH_2__(B)_), 1.29 (d, *J* = 6.6 Hz, 3H, H-6); ^13^C NMR (126 MHz, CDCl_3_) δ 196.4 (C=O), 102.5 (C-1), 74.5 (C-2), 72.7 (C-3 or C-4), 70.0 (C-3 or C-4), 65.6 (C-5), 55.3 (OCH_3_), 33.3 (-CH_2_-), 30.8 (CH_3(SAc)_), 29.7 (CH_2_-SAc), 22.4 (C_(sugar)_-CH_2_), 16.5 (C-6); HRMS (ESI) *m*/*z* calculated for C_12_H_22_O_6_S [M + Na]^+^ 317.1035, found 317.1024.

### 3.9. Methyl 2-C-[2-thioethyl]-α-l-fucopyranoside *(**1**)*

Compound **12** was subjected to **General Procedure B** (14 mg, 50 µmol) to afford **1** (11 mg, 92%) as a colourless, amorphous solid. R*_f_* = 0.5 (CH_2_Cl_2_/MeOH, 19:1); [α]${}_{D}^{20}$−78 (*c* 0.9, CH_3_OH); ^1^H NMR (400 MHz, CD_3_OD) δ 4.52 (s, 1H, H-1), 3.95 (m, 1H, H-5), 3.82 (d, *J* = 3.9 Hz, 1H, H-4), 3.70 (dd, *J* = 3.9, 1.6 Hz, 1H, H-3), 3.40 (s, 3H, OCH_3_), 2.73 (m, 1H, CH_2__(A)_-SH), 2.59 (m, 1H, CH_2__(B)_-SH), 2.25 (ddd, *J* = 14.5, 11.8, 5.3 Hz, 1H, C_(sugar)_-CH_2__(A)_), 2.15 (ddd, *J* = 14.5, 11.8, 5.3 Hz, 1H, C_(sugar)_-CH_2__(B)_), 1.25 (d, *J* = 6.6 Hz, 3H, H-6); ^13^C NMR (Taken from HSQC) δ 103.3 (C-1), 74.3 (C-4), 74.1 (C-3), 67.7 (C-5), 56.3 (OCH_3_), 39.9 (C_(sugar)_-CH_2_), 20.1 (CH_2_-SH), 16.8 (C-6); HRMS (ESI) *m*/*z* calculated for C_9_H_18_O_s_S [M + Na]^+^ 261.0773, found 261.0767.

### 3.10. Methyl 2-C-[3-thiopropyl]-α-l-fucopyranoside *(**2**)*

Compound **13** (33 mg, 0.11 mmol) was subjected to **General Procedure B** to afford **2** (26 mg, 92%) as a colourless, amorphous solid. R*_f_* = 0.4 (CH_2_Cl_2_/MeOH, 19:1); [α]${}_{D}^{20}$−78 (*c* 0.9, CH_3_OH); ^1^H NMR (400 MHz, CD_3_OD) δ 4.53 (s, 1H, H-1), 3.97 (m, 1H, H-5), 3.83 (d, *J* = 4.0 Hz, 1H, H-4), 3.72 (dd, *J* = 4.0, 1.9 Hz, 1H, H-3), 3.41 (s, 3H, OCH_3_), 2.52 (t, *J* = 7.3 Hz, 2H, CH_2_-SH), 2.00–1.90 (m, 2H, -CH_2_-), 1.83 (m, 1H, C_(sugar)-_CH_2__(A)_), 1.65 (m, 1H, C_(sugar)-_CH_2__(B)_), 1.25 (d, *J* = 6.7 Hz, 3H, H-6); ^13^C NMR (Taken from HSQC) δ 103.0 (C-1), 74.2 (C-4), 73.4 (C-3), 67.4 (C-5), 56.1 (OCH_3_), 32.1 (-CH_2_-), 29.3 (C_(sugar)_-CH_2_), 26.3 (CH_2_-SH), 16.6 (C-6); HRMS (ESI) *m*/*z* calculated for C_10_H_20_O_5_S [M + Na]^+^ 275.0929, found 275.0919.

### 3.11. Methyl 6-deoxy-2-C-[2-thioethyl]-α-l-talopyranoside *(**3**)*

Compound **14** (10 mg, 36 µmol) was subjected to **General Procedure B** to afford **3** (8 mg, 92%) as a colourless, amorphous solid. R*_f_* = 0.5 (CH_2_Cl_2_/MeOH, 19:1); [α]${}_{D}^{20}$−57 (*c* 0.9, CH_3_OH); ^1^H NMR (500 MHz, CD_3_OD) δ 4.48 (s, 1H, H-1), 3.89 (m, 1H, H-5), 3.61 (d, *J* = 2.5 Hz, 1H, H-4), 3.50 (d, *J* = 3.3 Hz, 1H, H-3), 3.37 (s, 3H, OCH_3_), 2.69 (td, *J* = 12.3, 4.4 Hz, 1H, CH_2__(A)_-SH), 2.50 (td, *J* = 12.3, 5.3 Hz, 1H, CH_2__(B)_-SH), 2.12 (ddd, *J* = 13.8, 12.3, 4.4 Hz, 1H, C_(sugar)_-CH_2__(A)_), 1.82 (ddd, *J* = 13.8, 12.3, 5.2 Hz, 1H, C_(sugar)_-CH_2__(B)_), 1.26 (d, *J* = 6.6 Hz, 3H, H-6); ^13^C NMR (Taken from HSQC) δ 105.1 (C-1), 74.8 (C-4), 71.0 (C-3), 68.0 (C-5), 55.9 (OCH_3_), 41.4 (C_(sugar)_-CH_2_), 19.3 (CH_2_-SH), 17.3 (C-6); HRMS (ESI) *m*/*z* calculated for C_9_H_18_O_s_S [M + Na]^+^ 261.0773, found 261.0773.

### 3.12. Methyl 6-deoxy-2-C-[3-thiopropyl]-α-l-talopyranoside *(**4**)*

Compound **15** (30 mg, 0.10 mmol) was subjected to **General Procedure B** to afford **4** (24 mg, 95%) as a colourless, amorphous solid. R*_f_* = 0.4 (CH_2_Cl_2_/MeOH, 19:1); [α]${}_{D}^{20}$−71 (*c* 0.8, CH_3_OH); ^1^H NMR (400 MHz, CD_3_OD) δ 4.48 (s, 1H, H-1), 3.90 (m, 1H, H-5), 3.62 (dd, *J* = 3.4, 1.0 Hz, 1H, H-4), 3.51 (d, *J* = 3.4 Hz, 1H, H-3), 3.38 (s, 3H, OCH_3_), 2.56–2.44 (m, 2H, CH_2_-SH), 1.97–1.73 (m, 2H, C_(sugar)_-CH_2__(A)_, -CH_2_-_(A)_), 1.67–1.53 (m, 2H, C_(sugar)_-CH_2__(B)_, -CH_2_-_(B)_), 1.27 (d, *J* = 6.6 Hz, 3H, H-6); ^13^C NMR (Taken from HSQC) δ 104.9 (C-1), 74.7 (C-4), 71.3 (C-3), 68.0 (C-5), 56.0 (OCH_3_), 34.8 (-CH_2_-), 29.0 (C_(sugar)_-CH_2_), 26.3 (CH_2_-SH), 17.3 (C-6); HRMS (ESI) *m*/*z* calculated for C_10_H_20_O_5_S [M + Na]^+^ 275.0929, found 275.0917.

### 3.13. Methyl 4-O-acetyl-2,3-O-(2′,3′-dimethoxybutane-2′,3′-diyl)-α-l-quinovopyranoside *(**17**)*

Compound **16** (6.09 g, 20.6 mmol) was placed under N_2_ and dissolved in dry CH_2_Cl_2_ (90 mL) and dry pyridine (14 mL). The solution was cooled to −40 °C and trifluoromethanesulfonic anhydride (4.2 mL, 25 mmol) was added. The reaction was stirred at −40 °C for 7 h, then allowed to reach room temperature. The organic phase was washed with sat. NaHCO_3_ solution (100 mL) and brine (100 mL), dried over MgSO_4_, filtered and concentrated in vacuo. The dark red/brown oil obtained was immediately used in the next step.

The crude and tetrabutylammonium acetate (7.55 g, 25.0 mmol) were placed under N_2_ and dry toluene (60 mL) was added. The reaction was stirred for 14 h at room temperature and the solvent was then removed under reduced pressure. The product was purified via flash chromatography on silica gel (cyclohexane/EtOAc, 4:1→1:1) to yield **17** a pale-yellow syrup (4.71 g, 68% over 2 steps). R*_f_* = 0.7 (cyclohexane/EtOAc, 1:1); [α]${}_{D}^{20}$+47 (*c* 1.0, CHCl_3_); ^1^H NMR (300 MHz, CDCl_3_) δ 4.80 (t, *J* = 9.7 Hz, 1H, H-4), 4.69 (d, *J* = 3.6 Hz, 1H, H-1), 4.06 (t, *J* = 9.7 Hz, 1H, H-3), 3.84–3.72 (m, 2H, H-2, H-5), 3.41 (s, 3H, OCH_3_), 3.24 (s, 3H, OCH_3_), 3.22 (s, 3H, OCH_3_), 2.07 (s, 3H, CH_3(OAc)_), 1.32 (s, 3H, CH_3(BDA)_), 1.24 (s, 3H, CH_3(BDA)_), 1.17 (d, *J* = 6.3 Hz, 3H, H-6); ^13^C NMR (126 MHz, CDCl_3_) δ 169.9 (C=O), 100.0, 99.5 (2 × C_(ketal)_), 97.9 (C-1), 73.3 (C-4), 68.7 (C-2), 67.1 (C-3), 66.1 (C-5), 55.2, 48.0, 47.6 (3 × OCH_3_), 21.0 (CH_3(OAc)_), 17.9, 17.8 (2 × CH_3(BDA)_), 17.5 (C-6); HRMS (ESI) *m*/*z* calculated for C_15_H_26_O_8_ [M + Na]^+^ 357.1525, found 357.1523.

### 3.14. Methyl 2,3-O-(2′,3′-dimethoxybutane-2′,3′-diyl)-α-l-quinovopyranoside *(**18**)*

Compound **17** (3.33 g, 9.96 mmol) was dissolved in MeOH (100 mL) and NaOMe (293 mg, 5.06 mmol) was added. The reaction was stirred for 19 h at room temperature and then Amberlite^®^ IR120 resin (H^+^ form) was added to neutralise the solution. The mixture was filtered, and the filtrate was concentrated under reduced pressure. The resulting yellow syrup was purified via flash chromatography on silica gel (toluene→toluene/EtOAc, 1:1) to yield **18** as a white foam (2.74 g, 94%). R*_f_* = 0.4 (cyclohexane/EtOAc, 1:1); [α]${}_{D}^{20}$+59 (*c* 1.0, CHCl_3_); ^1^H NMR (300 MHz, CDCl_3_) δ 4.68 (d, *J* = 3.5 Hz, 1H, H-1), 3.95 (dd, *J* = 10.4, 9.2 Hz, 1H, H-3), 3.74–3.63 (m, 2H, H-2, H-5), 3.40 (s, 3H, OCH_3_), 3.35 (m, 1H, H-4), 3.27 (s, 3H, OCH_3_), 3.24 (s, 3H, OCH_3_), 2.23 (d, *J* = 2.8 Hz, 1H, OH), 1.33 (s, 3H, CH_3(BDA)_), 1.30 (s, 3H, CH_3(BDA)_), 1.28 (d, *J* = 6.3 Hz, 3H, H-6); ^13^C NMR (126 MHz, CDCl_3_) δ 100.0, 99.6 (2 × C_(ketal)_), 98.1 (C-1), 73.8 (C-4), 69.5 (C-3), 68.6 (C-2), 67.9 (C-5), 55.1, 48.1, 48.0 (3 × OCH_3_), 18.0, 17.8 (2 × CH_3(BDA)_), 17.7 (C-6); HRMS (ESI) *m*/*z* calculated for C_13_H_24_O_7_ [M + Na]^+^ 315.1420, found 315.1410.

### 3.15. Methyl 4-azido-4-deoxy-2,3-O-(2′,3′-dimethoxybutane-2′,3′-diyl)-α-l-fucopyranoside *(**19**)*

Compound **18** (3.96 g, 13.6 mmol) was placed under an N_2_ atmosphere and dissolved in dry CH_2_Cl_2_ (37 mL) and dry pyridine (8 mL). The solution was cooled to -20 °C and trifluoromethanesulfonic anhydride (2.6 mL, 16 mmol) was added slowly. The reaction was stirred at -20 °C for 4 h, then allowed to reach room temperature. The solution was then diluted with CH_2_Cl_2_ (35 mL) and washed with sat. aq. NaHCO_3_ (70 mL) and brine (70 mL). The combined aqueous phase was extracted with CH_2_Cl_2_ (70 mL) and the collective organic layer was dried over MgSO_4_, filtered and concentrated. The crude was used immediately in the next step.

The crude triflate and sodium azide (5.31 g, 81.7 mmol) were placed under N_2_ and dry DMF (110 mL) was added. The resulting suspension was stirred at 60 °C for 17 h and the solvent was then removed in vacuo. EtOAc (100 mL) and water (100 mL) were added to the resulting residue. The organic layer was separated and then washed with brine (100 mL). The collective aqueous phase was extracted with EtOAc (2 × 50 mL) and the combined organic layer was dried over MgSO_4_, filtered and concentrated under reduced pressure. The product was purified by flash chromatography on silica gel (cyclohexane/EtOAc, 3:1) to yield **19** as a pale-yellow syrup (3.00 g, 70% over 2 steps). R*_f_* = 0.4 (cyclohexane/EtOAc, 3:1); [α]${}_{D}^{20}$+86 (*c* 1.0, CHCl_3_); ^1^H NMR (300 MHz, CDCl_3_) δ 4.71 (d, *J* = 3.6 Hz, 1H, H-1), 4.21 (dd, *J* = 10.5, 3.5 Hz, 1H, H-3), 4.11 (dd, *J* = 10.5, 3.6 Hz, 1H, H-2), 3.99 (m, 1H, H-5), 3.64 (dd, *J* = 3.5, 3.2 Hz, 1H, H-4), 3.37 (s, 3H, OCH_3_), 3.27 (s, 3H, OCH_3_), 3.26 (s, 3H, OCH_3_), 1.31 (s, 3H, CH_3(BDA)_), 1.29 (s, 3H, CH_3(BDA)_), 1.26 (d, *J* = 6.5 Hz, 3H, H-6); ^13^C NMR (101 MHz, CDCl_3_) δ 100.3, 100.2 (2 × C_(ketal)_), 98.4 (C-1), 67.0 (C-3), 65.50 (C-5), 65.49 (C-2), 63.8 (C-4), 55.4, 48.14, 48.11 (2 × OCH_3_), 17.9, 17.8 (2 × CH_3(BDA)_), 17.4 (C-6); HRMS (ESI) *m*/*z* calculated for C_13_H_23_N_3_O_6_ [M + Na]^+^ 340.1485, found 340.1469.

### 3.16. Methyl 4-amino-4-deoxy-2,3-O-(2′,3′-dimethoxybutane-2′,3′-diyl)-α-l-fucopyranoside *(**20**)*

Compound **19** (2.74 g, 8.63 mmol) was dissolved in EtOH (15 mL). 10% Pd/C (0.54 g, 0.51 mmol) suspended in EtOH (5 mL) was added and the mixture was stirred under H_2_ (5 bar) at room temperature for 22 h. The suspension was then filtered through Celite^®^ and the filtrate was concentrated. Flash chromatography on silica gel (CH_2_Cl_2_/MeOH, 49:1) yielded 20 as a white solid (2.28 g, 91%). R*_f_ =* 0.7 (CH_2_Cl_2_/MeOH, 19:1); mp = 87–89 °C; [α]${}_{D}^{20}$+41 (*c* 1.0, MeOH); ^1^H NMR (300 MHz, CD_3_OD) δ 4.66 (d, *J* = 2.2 Hz, 1H, H-1), 4.07–3.97 (m, 3H, H-2, H-3, H-5), 3.38 (s, 3H, OCH_3_), 3.25 (s, 3H, OCH_3_), 3.24 (s, 3H, OCH_3_), 2.98 (t, *J* = 1.7 Hz, 1H, H-4), 1.28–1.27 (m, 6H, 2 × CH_3(BDA)_), 1.21 (d, *J* = 6.6 Hz, 3H, H-6); ^13^C NMR (126 MHz, CD_3_OD) δ 101.5, 101.3 (2 × C_(ketal)_), 99.7 (C-1), 67.8 (C-2 or C-3), 67.4 (C-5), 66.1 (C-2 or C-3), 55.4 (OCH_3_), 54.1 (C-4), 48.22, 48.15 (2 × OCH_3_), 18.1, 18.0 (2 × CH_3(BDA)_), 16.9 (C-6); HRMS (ESI) *m*/*z* calculated for C_13_H_25_NO_6_ [M + H]^+^ 292.1760, found 292.1761.

### 3.17. 2-Bromoethyl Thioacetate *(**21**)*

Dry THF (40 mL) was added to potassium thioacetate (2.00 g, 17.5 mmol) under N_2_. 1,2-Dibromoethane (3.1 mL, 36 mmol) was added and the mixture was refluxed at 90 °C for 22 h. The suspension was then filtered through Celite^®^, and the filtrate was concentrated under reduced pressure. The resulting yellow residue was purified via flash chromatography on silica gel (cyclohexane/EtOAc, 19:1), yielding compound **21** as a colourless oil (1.18 g, 37%). R*_f_* = 0.5 (cyclohexane/EtOAc, 10:1); ^1^H NMR (400 MHz, CDCl_3_) δ 3.44 (m, 2H, CH_2_-Br), 3.30 (m, 2H, CH_2_-S), 2.35 (s, 3H, CH_3(SAc)_); ^13^C NMR (101 MHz, CDCl_3_) δ 194.7 (C=O_(SAc)_), 31.4 (CH_2_), 30.7 (CH_3(SAc)_), 30.1 (CH_2_). While the chemical shifts of the ^1^H NMR data match those reported in literature, we observed the CH_2_ signals as multiplets rather than triplets [28]. No ^13^C NMR data have been reported previously.

### 3.18. Methyl 4-[(2-acetylthioethyl)amino]-4-deoxy-2,3-O-(2′,3′-dimethoxybutane-2′,3′-diyl)-α-l-fucopyranoside *(**22**)*

Compound **20** (0.50 g, 1.7 mmol) and K_2_CO_3_ (1.19 g, 8.57 mmol) were placed under N_2_ and dry DMF (5 mL) was added. Alkyl bromide **21** (610 mg, 3.33 mmol) in dry DMF (5 mL) was added followed by KI (286 mg, 1.72 mmol) and the resulting suspension was stirred at room temperature for 4 days. The solvent was removed in vacuo and water (25 mL) was added. The aqueous phase was extracted with EtOAc (3 × 25 mL) and the combined organic layer was dried over Na_2_SO_4_, filtered and concentrated. The product was purified by flash chromatography on silica gel (toluene/EtOAc, 7:3), yielding **22** as a pale-yellow syrup (271 mg, 40%). R*_f_* = 0.4 (cyclohexane/EtOAc, 1:1); [α]${}_{D}^{20}$−8.2 (*c* 1.0, CHCl_3_); ^1^H NMR (500 MHz, CDCl_3_) δ 4.71 (d, *J* = 3.8 Hz, 1H, H-1), 4.12 (dd, *J* = 10.9, 4.4 Hz, 1H, H-3), 3.98–3.90 (m, 2H, H-2, H-5), 3.37 (s, 3H, OCH_3_), 3.24 (s, 3H, OCH_3_), 3.22 (s, 3H, OCH_3_), 3.07 (m, 1H, NH-CH_2__(A)_), 2.98 (m, 1H, NH-CH_2__(B)_), 2.93–2.84 (m, 2H, CH_2_-SAc), 2.73 (dd, *J* = 4.5, 4.4 Hz, 1H, H-4), 2.32 (s, 3H, CH_3(SAc)_), 1.31 (s, 3H, CH_3(BDA)_), 1.25 (s, 3H, CH_3(BDA)_), 1.24 (d, *J* = 6.7 Hz, 3H, H-6); ^13^C NMR (101 MHz, CDCl_3_) δ 196.1 (C=O_(SAc)_), 100.1, 99.8 (2 × C_(ketal)_), 98.3 (C-1), 67.1 (C-5), 66.5 (C-3), 65.6 (C-2), 60.1 (C-4), 55.1 (OCH_3_), 50.3 (CH_2_-SAc), 48.01, 47.99 (2 × OCH_3_), 30.8 (CH_3(SAc)_), 29.7 (NH-CH_2_), 17.91, 17.89 (2 × CH_3(BDA)_), 17.5 (C-6); HRMS (ESI) *m*/*z* calculated for C_17_H_31_NO_7_S [M + Na]^+^ 416.1719, found 416.1721.

### 3.19. Methyl 4-[(2-acetylthioethyl)amino]-4-deoxy-α-l-fucopyranoside *(**23**)*

Compound **22** (20 mg, 52 µmol) was stirred in TFA/H_2_O (1.7 mL, 9:1, *v*/*v*) at room temperature for 2.5 h. The solvents were then removed through co-evaporation with toluene under reduced pressure. The resulting residue was dissolved in CH_2_Cl_2_ (1.8 mL) and stirred with Amberlyst^®^ A21 resin (197 mg) at room temperature for 18 h. The resin was then filtered off and washed with MeOH. The filtrate was concentrated in vacuo and the product was purified by flash chromatography on silica gel (EtOAc→EtOAc/MeOH, 19:1), furnishing **23** as a colourless syrup (11 mg, 77%). R*_f_* = 0.5 (EtOAc/MeOH, 9:1); ^1^H NMR (500 MHz, CDCl_3_) δ 4.70 (d, *J* = 4.0 Hz, 1H, H-1), 4.06 (m, 1H, H-5), 3.63 (dd, *J* = 9.8, 4.6 Hz, 1H, H-3), 3.40 (s, 3H, OCH_3_), 3.38 (dd, *J* = 9.8, 4.0 Hz, 1H, H-2), 3.23 (m, 1H, CH_2__(A)_-SAc), 3.04–2.95 (m, 2H, NH-CH_2_), 2.73–2.64 (m, 2H, H-4, CH_2__(B)_-SAc), 2.36 (s, 3H, CH_3(SAc)_), 1.26 (d, *J* = 6.7 Hz, 3H, H-6); ^13^C NMR (126 MHz, CDCl_3_) δ 195.8 (C=O_(SAc)_), 99.6 (C-1), 71.3 (C-2), 70.7 (C-3), 66.3 (C-5), 62.6 (C-4), 55.6 (OCH_3_), 50.7 (CH_2_-SAc), 30.83 (CH_3(SAc)_), 30.82 (NH-CH_2_), 17.6 (C-6); HRMS (ESI) *m*/*z* calculated for C_11_H_21_NO_5_S [M + H]^+^ 280.1219, found 280.1208.

### 3.20. 2-Bromoethyl thiobenzoate *(**24**)*

Potassium thiobenzoate (0.51 g, 2.9 mmol) was placed under N_2_ and dry THF (28 mL) was added followed by 1,2-dibromoethane (2.4 mL, 28 mmol). The reaction was refluxed at 90 °C for 3 h, cooled to room temperature and the solids were removed by filtration through Celite^®^. The filtrate was concentrated in vacuo and purified by flash chromatography on silica gel (cyclohexane/toluene, 4:1). Compound **24** was obtained as a colourless oil (603 mg, 86%). R*_f_* = 0.4 (cyclohexane/toluene, 7:3); ^1^H NMR (300 MHz, CDCl_3_) δ 7.99–7.92 (m, 2H, Ar-H), 7.58 (m, 1H, Ar-H), 7.52–7.39 (m, 2H, Ar-H), 3.67–3.40 (m, 4H, CH_2_-Br, CH_2_-S); ^13^C NMR (101 MHz, CDCl_3_) δ 190.7 (C=O_(SBz)_), 136.6 (Ar-C_(quat)_), 133.9, 128.9, 127.5 (3 × Ar-CH), 31.2 (CH_2_-S), 30.2 (CH_2_-Br).

### 3.21. Methyl 4-[(2-benzoylthioethyl)amino]-4-deoxy-2,3-O-(2′,3′-dimethoxybutane-2′,3′-diyl)-α-l-fucopyranoside *(**25**)*

Compound **20** (351 mg, 1.20 mmol) was placed under N_2_ and dissolved in dry DMF (6 mL). K_2_CO_3_ (183 mg, 1.33 mmol) was added followed by a solution of alkyl bromide **24** (326 mg, 1.33 mmol) in dry DMF (6 mL). KI (97 mg, 0.58 mmol) was then added and the reaction was stirred at 80 °C for 20 h. The solvent was then removed in vacuo and water (30 mL) was added to the resulting residue. The product was extracted with EtOAc (3 × 30 mL) and the combined organic layer was dried over MgSO_4_, filtered and concentrated under reduced pressure. Flash chromatography on silica gel (toluene/acetone, 19:1) yielded compound **25** as a colourless syrup (267 mg, 49%). R*_f_* = 0.5 (toluene/EtOAc, 1:1); [α]${}_{D}^{20}$+14 (*c* 1.0, CHCl_3_); ^1^H NMR (400 MHz, CDCl_3_) δ 8.00–7.93 (m, 2H, Ar-H), 7.56 (m, 1H, Ar-H), 7.49–7.38 (m, 2H, Ar-H), 4.73 (d, *J* = 3.8 Hz, 1H, H-1), 4.14 (dd, *J* = 10.8, 4.4 Hz, 1H, H-3), 3.99 (dd, *J* = 10.8, 3.8 Hz, 1H, H-2), 3.94 (m, 1H, H-5), 3.38 (s, 3H, OCH_3_), 3.32–3.14 (m, 8H, 2 × OCH_3_, NH-CH_2__(A)_, NH-CH_2__(B)_), 3.03–2.96 (m, 2H, CH_2_-SBz), 2.79 (dd, *J* = 4.4, 1.6 Hz, 1H, H-4), 1.31 (s, 3H, CH_3(BDA)_), 1.26 (d, *J* = 6.7 Hz, 3H, H-6), 1.24 (s, 3H, CH_3(BDA)_); ^13^C NMR (101 MHz, CDCl_3_) δ 192.1 (C=O_(SBz)_), 137.3 (Ar-C_(quat)_), 133.4, 128.7, 127.3 (3 × Ar-CH), 100.1, 99.8 (2 × C_(ketal)_), 98.3 (C-1), 67.2 (C-5), 66.6 (C-3), 65.6 (C-2), 60.1 (C-4), 55.1 (OCH_3_), 50.4 (CH_2_-SBz), 48.0 (2 × OCH_3_), 29.6 (NH-CH_2_), 17.90, 17.88 (2 × CH_3(BDA)_), 17.5 (C-6). HRMS (ESI) m/z calculated for C_22_H_33_NO_7_S [M + H]^+^ 456.2056, found 456.2068.

### 3.22. Methyl 4-[(2-benzoylthioethyl)amino]-4-deoxy-α-l-fucopyranoside *(**26**)*

Compound **25** (52 mg, 0.11 mmol) was stirred in TFA/H_2_O (3.7 mL, 9:1, *v*/*v*) at room temperature for 3 h. The solvents were then co-evaporated with toluene under reduced pressure. The resulting residue was re-dissolved in CH_2_Cl_2_ (5 mL) and stirred at room temperature with Amberlyst^®^ A21 resin (507 mg) for 20 h. The resin was then filtered off and washed with CH_2_Cl_2_/MeOH (1:1, *v*/*v*). The filtrate was concentrated in vacuo and purified via flash chromatography on silica gel (toluene/EtOAc, 1:4→EtOAc), yielding **26** as a white amorphous solid (30 mg, 76%). R*_f_* = 0.3 (EtOAc); [α]${}_{D}^{20}$−120 (*c* 1.0, CHCl_3_); ^1^H NMR (500 MHz, CDCl_3_) δ 7.98–7.93 (m, 2H, Ar-H), 7.57 (m, 1H, Ar-H), 7.48–7.41 (m, 2H, Ar-H), 4.69 (d, *J* = 4.0 Hz, 1H, H-1), 4.06 (m, 1H, H-5), 3.63 (dd, *J* = 9.8, 4.7 Hz, 1H, H-3), 3.42–3.37 (m, 4H, H-2, OCH_3_), 3.31 (m, 1H, CH_2__(A)_-SBz), 3.22–3.19 (m, 2H, NH-CH_2_), 2.81–2.71 (m, 2H, H-4, CH_2__(B)_-SBz), 1.26 (d, *J* = 6.7 Hz, 3H, H-6); ^13^C NMR (126 MHz, CDCl_3_) δ 191.8 (C=O_(SBz)_), 137.0 (Ar-C_(quat)_), 133.7 (Ar-CH), 128.8 (Ar-CH), 127.4 (Ar-CH), 99.6 (C-1), 71.3 (C-2), 70.7 (C-3), 66.3 (C-5), 62.6 (C-4), 55.6 (OCH_3_), 50.8 (CH_2_-SBz), 30.7 (NH-CH_2_), 17.6 (C-6). HRMS (ESI) *m*/*z* calculated for C_16_H_23_NO_5_S [M + H]^+^ 342.1375, found 342.1366.

### 3.23. Methyl 4-deoxy-4-(2-thioethyl)amino-α-l-fucopyranoside *(**5**)*

Compound **26** (20 mg, 57 µmol) was placed under N_2_. Dry, de-gassed MeOH (0.58 mL) was added followed by freshly prepared, de-gassed 1 M NaOMe/MeOH (0.15 mL, 0.15 mmol). The reaction was stirred at room temperature for 15 min and then neutralised with Amberlite^®^ IR120 resin (H^+^ form). The resin was filtered off and the filtrate was concentrated. The product was purified by flash chromatography through a short column of silica gel (CH_2_Cl_2_/MeOH, 19:1→9:1) under an N_2_ atmosphere and using de-gassed solvents. Compound **5** was obtained as a colourless residue (8 mg, 58% by mass, 95% thiol, 5% disulfide). R*_f_* = 0.2 (EtOAc/MeOH, 19:1); [α]${}_{D}^{20}$+2.7 (*c* 0.75, H_2_O); ^1^H NMR (500 MHz, D_2_O) δ 4.79 (d, 1H, H-1, underneath HDO peak), 4.14 (m, 1H, H-5), 3.97 (dd, *J* = 10.4, 4.3 Hz, 1H, H-3), 3.68 (dd, *J* = 10.4, 4.0 Hz, 1H, H-2), 3.40 (s, 3H, OCH_3_), 3.09 (m, 1H, CH_2__(A)_-SH), 2.99 (d, *J* = 4.3 Hz, 1H, H-4), 2.85 (m, 1H, CH_2__(B)_-SH), 2.77–2.67 (m, 2H, NH-CH_2_), 1.31 (d, *J* = 6.7 Hz, 3H, H-6); ^13^C NMR (126 MHz, D_2_O) δ 99.2 (C-1, *J*_C-1,H-1_ = 170.9 Hz), 68.8 (C-3), 68.0 (C-2), 65.9 (C-5), 61.8 (C-4), 55.0 (OCH_3_), 53.4 (CH_2_-SH), 23.3 (NH-CH_2_), 16.5 (C-6); HRMS (ESI) *m*/*z* calculated for C_9_H_19_NO_4_S [M + H]^+^ 238.1113, found 238.1119.

### 3.24. Methyl 4-allylamino-4-deoxy-2,3-O-(2′,3′-dimethoxybutane-2′,3′-diyl)-α-l-fucopyranoside *(**27**)*

Compound **20** (374 mg, 1.28 mmol) and K_2_CO_3_ (196 mg, 1.42 mmol) were subjected to an N_2_ atmosphere. Dry DMF (7.5 mL) was added followed by allyl bromide (0.13 mL, 1.5 mmol). The resulting suspension was stirred at room temperature for 21 h and the solvent was then removed under reduced pressure. Water (25 mL) was added and aqueous phase was extracted with EtOAc (3 × 25 mL). The combined organic layer was dried over Na_2_SO_4_, filtered and concentrated in vacuo. The crude was purified by flash chromatography on silica gel (toluene/EtOAc, 4:1→7:3) to yield **27** as a pale-yellow, waxy solid (290 mg, 68%). R*_f_* = 0.3 (cyclohexane/EtOAc, 1:1); [α]${}_{D}^{20}$+28 (*c* 1.0, CHCl_3_); ^1^H NMR (500 MHz, CDCl_3_) δ 5.89 (m, 1H, CH_2_=CH), 5.19 (dq, *J* = 17.2, 1.4 Hz, 1H, CH_2__(A)_=CH), 5.06 (dq, *J* = 10.2, 1.4 Hz, 1H, CH_2__(B)_=CH), 4.72 (d, *J* = 3.8 Hz, 1H, H-1), 4.13 (dd, *J* = 10.8, 4.5 Hz, 1H, H-3), 3.99 (dd, *J* = 10.8, 3.8 Hz, 1H, H-2), 3.91 (m, 1H, H-5), 3.40 (ddt, *J* = 14.3, 5.4, 1.6 Hz, 1H, NH-CH_2__(A)_), 3.37 (s, 3H, OCH_3_), 3.27 (m, 1H, NH-CH_2__(B)_), 3.24 (s, 3H, OCH_3_), 3.21 (s, 3H, OCH_3_), 2.75 (dd, *J* = 4.5, 4.3 Hz, 1H, H-4), 1.30 (s, 3H, CH_3(BDA)_), 1.25–1.24 (m, 6H, CH_3(BDA)_, H-6); ^13^C NMR (126 MHz, CDCl_3_) δ 137.5 (CH_2_=CH), 115.7 (CH_2_=CH), 100.0, 99.8 (2 × C_(ketal)_), 98.3 (C-1), 67.3 (C-5), 66.5 (C-3), 65.6 (C-2), 59.5 (C-4), 55.1 (OCH_3_), 53.8 (NH-CH_2_), 47.98, 47.94 (2 × OCH_3_), 17.90, 17.88 (2 × CH_3(BDA)_), 17.80 (C-6); HRMS (ESI) *m*/*z* calculated for C_16_H_29_NO_6_ [M + H]^+^ 332.2073, found 332.2079.

### 3.25. Methyl 4-[(3-acetylthiopropyl)amino]-4-deoxy-2,3-O-(2′,3′-dimethoxybutane-2′,3′-diyl)-α-l-fucopyran -oside *(**28**)*

Compound **27** (281 mg, 0.849 mmol) and 1,2-dihydroxybenzene (116 mg, 1.05 mmol) were placed under N_2_ and dissolved in dry CH_2_Cl_2_ (1.1 mL). Thioacetic acid (0.12 mL, 1.7 mmol) was added followed by 1 M Et_3_B/hexanes (1.3 mL, 1.3 mmol). The reaction was stirred for 2.5 h at room temperature and then the volatiles were then removed under reduced pressure. The product was purified by flash chromatography on silica gel (toluene/EtOAc, 9:1→3:2), yielding **28** as a pale-yellow syrup (287 mg, 83%). R*_f_* = 0.2 (cyclohexane/EtOAc, 3:2); [α]${}_{D}^{20}$+8.4 (*c* 1.0, CHCl_3_); ^1^H NMR (500 MHz, CDCl_3_) δ 4.71 (d, *J* = 3.8 Hz, 1H, H-1), 4.11 (dd, *J* = 10.8, 4.4 Hz, 1H, H-3), 3.97 (dd, *J* = 10.8, 3.8 Hz, 1H, H-2), 3.92 (m, 1H, H-5), 3.36 (s, 3H, OCH_3_), 3.24 (s, 3H, OCH_3_), 3.21 (s, 3H, OCH_3_), 3.01–2.91 (m, 2H, CH_2_-SAc), 2.81–2.68 (m, 2H, NH-CH_2_), 2.66 (dd, *J* = 4.5, 4.4 Hz, 1H, H-4), 2.31 (s, 3H, CH_3(SAc)_), 1.83–1.68 (m, 2H, -CH_2_-), 1.30 (s, 3H, CH_3(BDA)_), 1.25 (s, 3H, CH_3(BDA)_), 1.23 (d, *J* = 6.7 Hz, 3H, H-6); ^13^C NMR (126 MHz, CDCl_3_) δ 196.1 (C=O_(SAc)_), 100.0, 99.8 (2 × C_(ketal)_), 98.3 (C-1), 67.2 (C-5), 66.6 (C-3), 65.6 (C-2), 60.8 (C-4), 55.1 (OCH_3_), 50.3 (CH_2_-SAc), 47.98, 47.97 (2 × OCH_3_), 30.7 (CH_3(SAc)_), 30.2 (-CH_2_-), 26.9 (NH-CH_2_), 17.92, 17.88 (2 × CH_3(BDA)_), 17.6 (C-6); HRMS (ESI) *m*/*z* calculated for C_18_H_33_NO_7_S [M + H]^+^ 408.2056, found 408.2076.

### 3.26. Methyl 4-[(3-acetylthiopropyl)amino]-4-deoxy-α-l-fucopyranoside *(**29**)*

Compound **28** (272 mg, 0.667 mmol) was stirred in TFA/H_2_O (22 mL, 9:1, *v*/*v*) at room temperature for 3 h. The solvents were then co-evaporated with toluene in vacuo. The resulting residue re-dissolved in CH_2_Cl_2_ (25 mL) and stirred at room temperature with Amberlyst^®^ A21 resin (2.42 g) for 16 h. The resin was then filtered off and washed with CH_2_Cl_2_/MeOH (1:1, *v*/*v*). The filtrate was concentrated in vacuo and purified via flash chromatography on silica gel (EtOAc→EtOAc/MeOH, 4:1) to yield compound **29** as a yellow syrup (133 mg, 68%). R*_f_* = 0.3 (EtOAc/MeOH, 9:1); [α]${}_{D}^{20}$−136 (*c* 1.0, CHCl_3_); ^1^H NMR (500 MHz, CDCl_3_) δ 4.69 (d, *J* = 4.0 Hz, 1H, H-1), 4.05 (m, 1H, H-5), 3.63 (dd, *J* = 9.8, 4.7 Hz, 1H, H-3), 3.39 (m, 4H, H-2, OCH_3_), 3.04–2.89 (m, 3H, NH-CH_2_, CH_2(A)_-SAc), 2.69 (dd, *J* = 4.8, 4.7 Hz, 1H, H-4), 2.58 (m, 1H, CH_2__(B)_-SAc), 2.33 (s, 3H, CH_3(SAc)_), 1.82–1.73 (m, 2H, -CH_2_-), 1.26 (d, *J* = 6.7 Hz, 3H, H-6); ^13^C NMR (126 MHz, CDCl_3_) δ 196.1 (C=O_(SAc)_), 99.6 (C-1), 71.1 (C-2), 70.4 (C-3), 66.2 (C-5), 62.6 (C-4), 55.6 (OCH_3_), 50.2 (CH_2_-SAc), 30.9 (-CH_2_-), 30.8 (CH_3(SAc)_), 26.7 (NH-CH_2_), 17.6 (C-6); HRMS (ESI) *m*/*z* calculated for C_12_H_23_NO_5_S [M + H]^+^ 294.1375, found 294.1364.

### 3.27. Methyl 4-deoxy-4-[(3-thiopropyl)amino]-α-l-fucopyranoside *(**6**)*

Compound **29** (27 mg, 91 µmol) was placed under N_2_ and dissolved in dry, de-gassed MeOH (0.90 mL). Freshly prepared, de-gassed 1 M NaOMe/MeOH (0.19 mL, 0.19 mmol) was then added and the reaction was stirred at room temperature for 30 min. The solution was then diluted with dry, de-gassed MeOH (1.0 mL), neutralised with activated Amberlite^®^ IR120 resin (H^+^ form), filtered and concentrated in vacuo. Compound **6** was obtained as a yellow/orange residue (20 mg, 88% by mass, 88% thiol, 12% disulfide). R*_f_* = 0.4 (CH_2_Cl_2_/MeOH, 9:1); [α]${}_{D}^{20}$−180 (*c* 1.0, H_2_O); ^1^H NMR (400 MHz, D_2_O) δ 4.79 (d, 1H, H-1, underneath HDO peak), 4.12 (m, 1H, H-5), 3.97 (dd, *J* = 10.5, 4.6 Hz, 1H, H-3), 3.66 (dd, *J* = 10.5, 4.0 Hz, 1H, H-2), 3.40 (s, 3H, OCH_3_), 3.03–2.88 (m, 2H, H-4, CH_2__(A)_-SH), 2.79 (m, 1H, CH_2__(B)_-SH), 2.61 (t, *J* = 7.0 Hz, 2H, NH-CH_2_), 2.01–1.79 (m, 2H, -CH_2_-), 1.31 (d, *J* = 6.8 Hz, 3H, H-6); ^13^C NMR (126 MHz, D_2_O) δ 99.2 (C-1, *J*_C-1,H-1_ = 170.8 Hz), 68.7 (C-3), 68.0 (C-2), 66.1 (C-5), 62.2 (C-4), 55.0 (OCH_3_), 49.8 (CH_2_-SH), 32.5 (-CH_2_-), 21.4 (NH-CH_2_), 16.5 (C-6); HRMS (ESI) *m*/*z* calculated for C_10_H_21_NO_4_S [M + H]^+^ 252.1270, found 252.1261.

## 4. Conclusions

In conclusion, we successfully synthesised six potential suicide inhibitors of BabA which are currently being evaluated for biological activity. Our attention has now turned to the synthesis of disaccharide inhibitors to include more binding interactions in CL2 of BabA, in theory creating stronger inhibitors.

## Supplementary Materials

The Supplementary Materials are available online.
