# Supplementary material for: Synthesis of Fucose Derivatives with Thiol Motifs towards Suicide Inhibition of Helicobacter pylori
Source: Molecules. 2020 Sep 18;25(18):4281. doi: 10.3390/molecules25184281 (PMC7571248; doi:10.3390/molecules25184281)
Supplement: Supplementary file 1 [file molecules-25-04281-s001.pdf]

Methyl 2-*C*-vinyl-α-L-fucopyranoside (8)

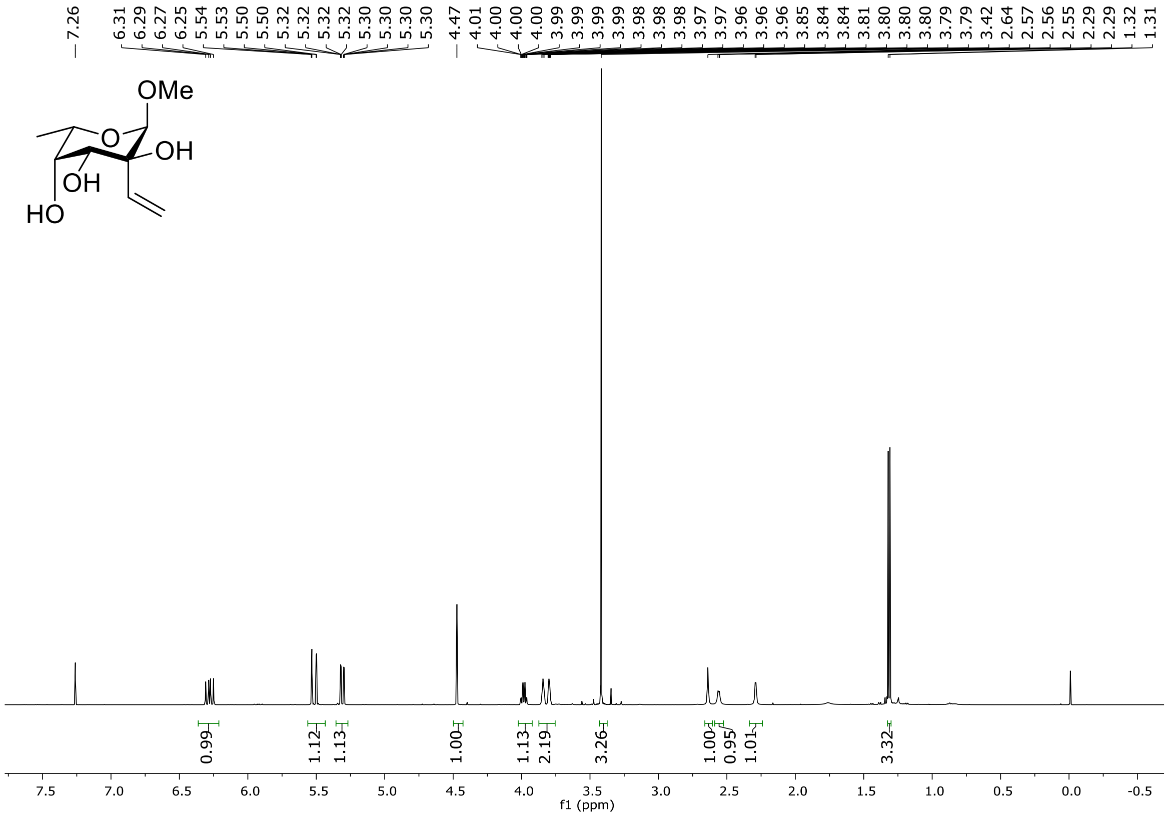

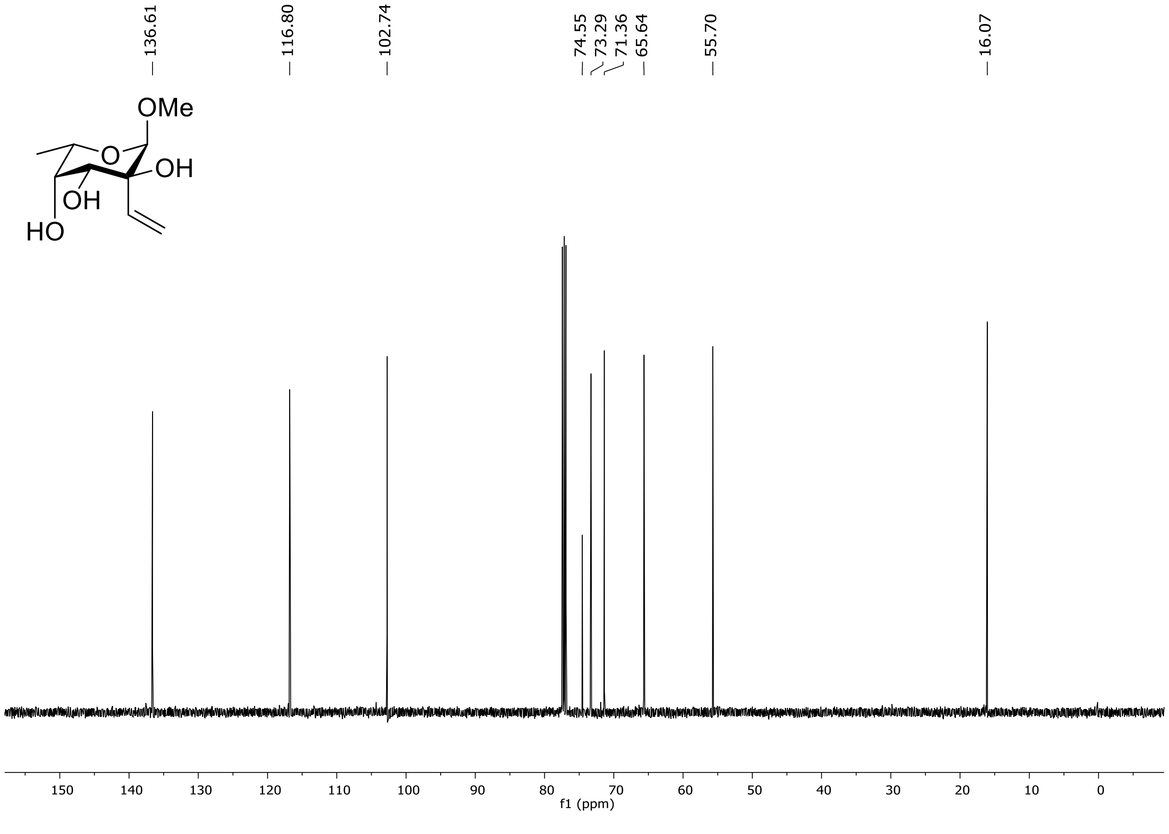

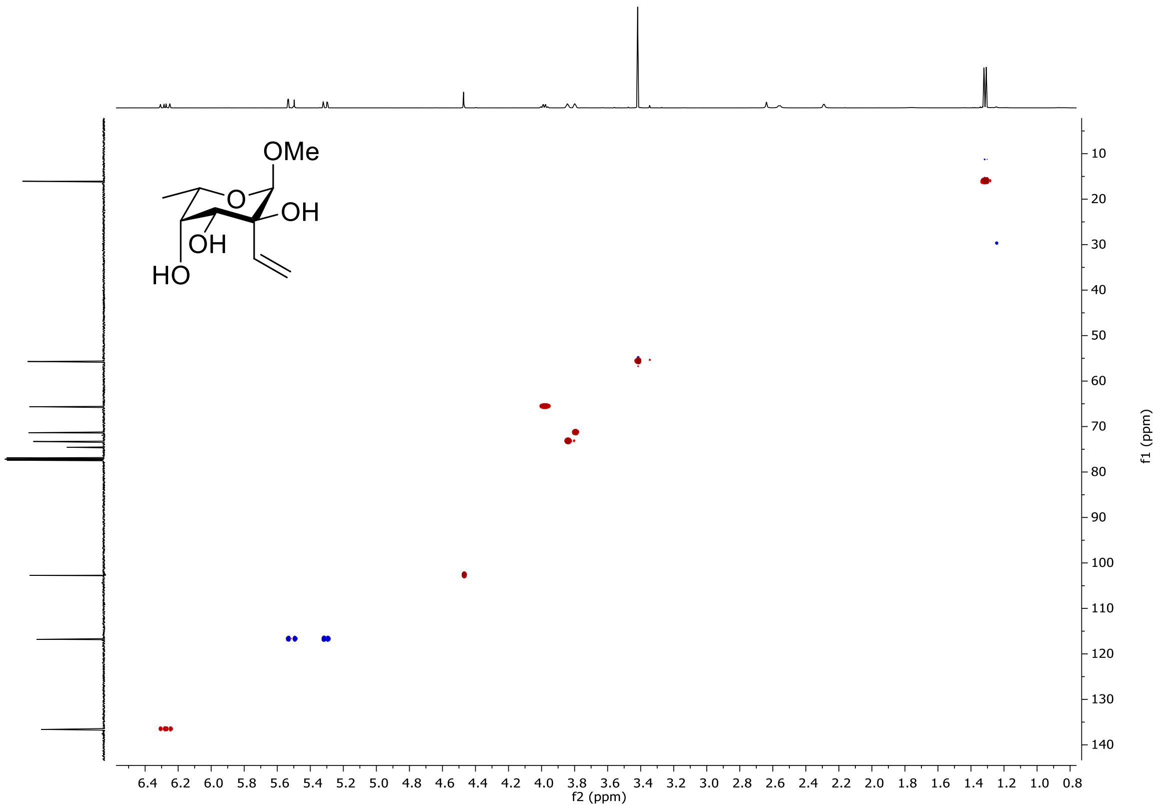

OMe
OH
OH
HO
f1 (ppm)
f2 (ppm)

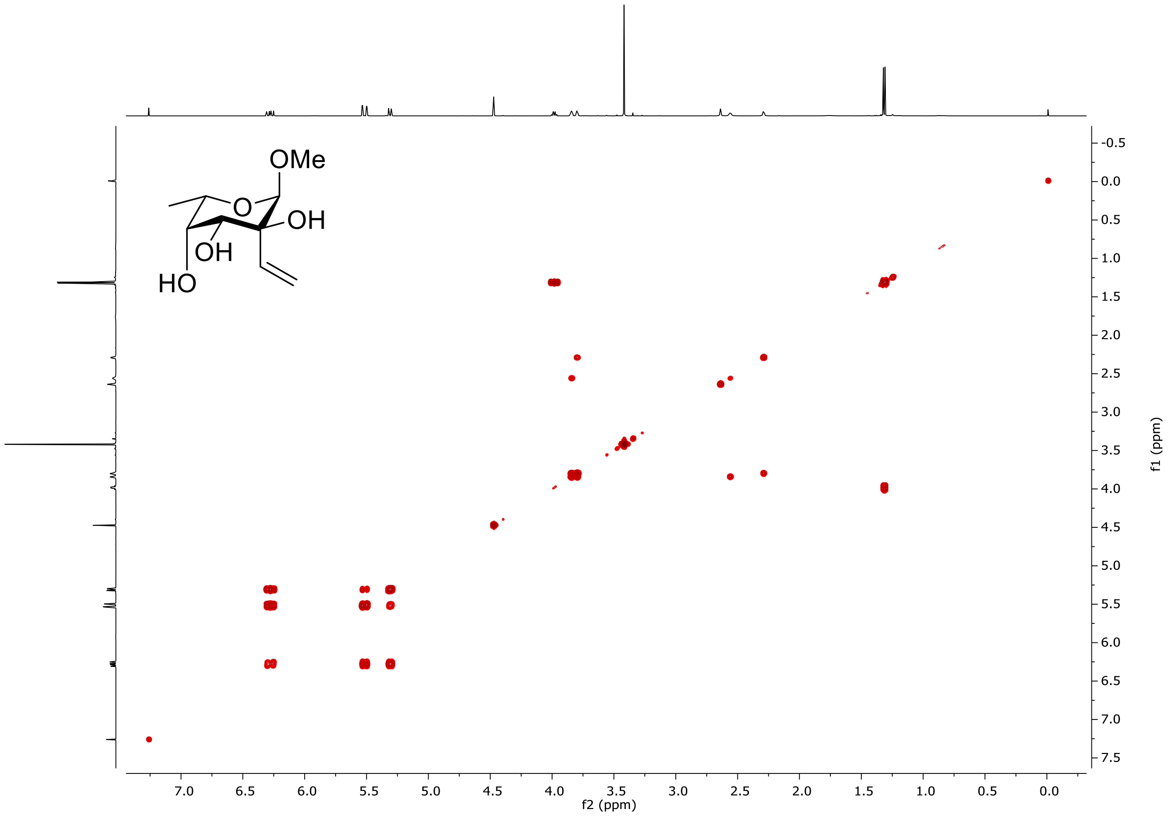

OMe
OH
OH
HO
f1 (ppm)
f2 (ppm)

Methyl 6-deoxy-2-*C*-vinyl-α-L-talopyranoside (10)

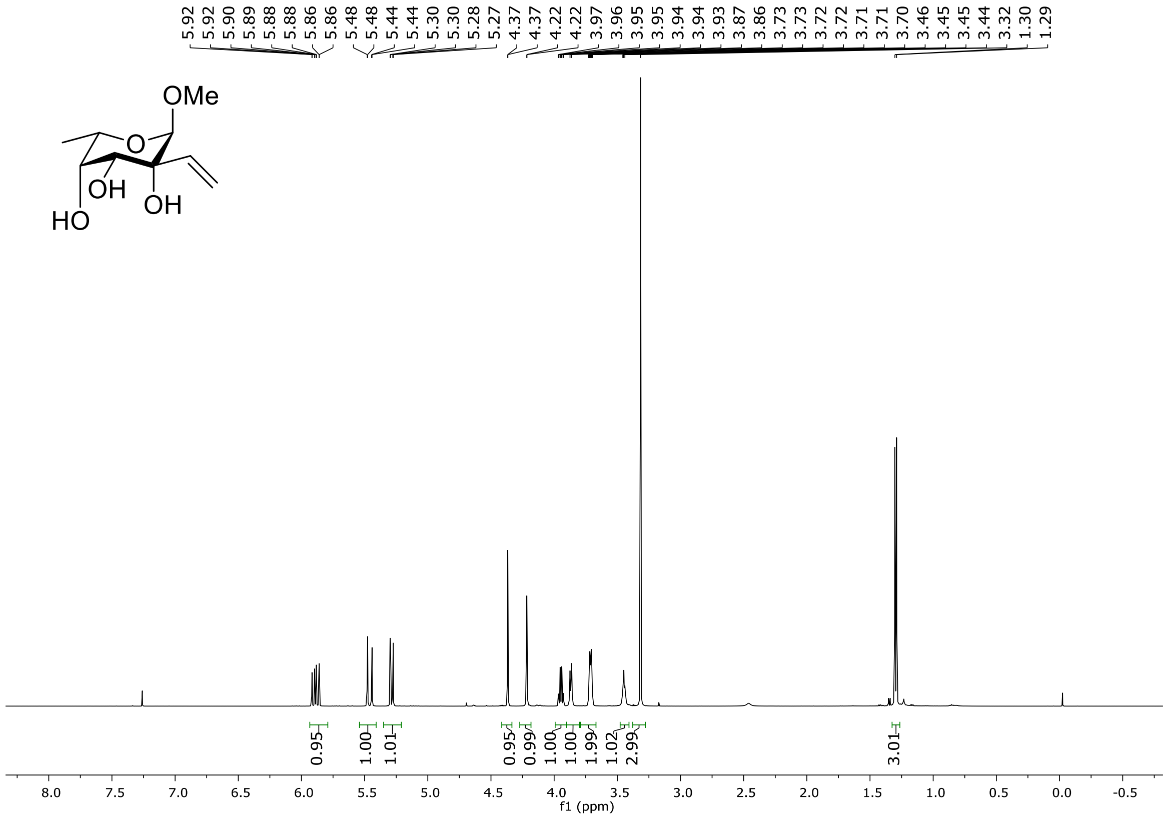

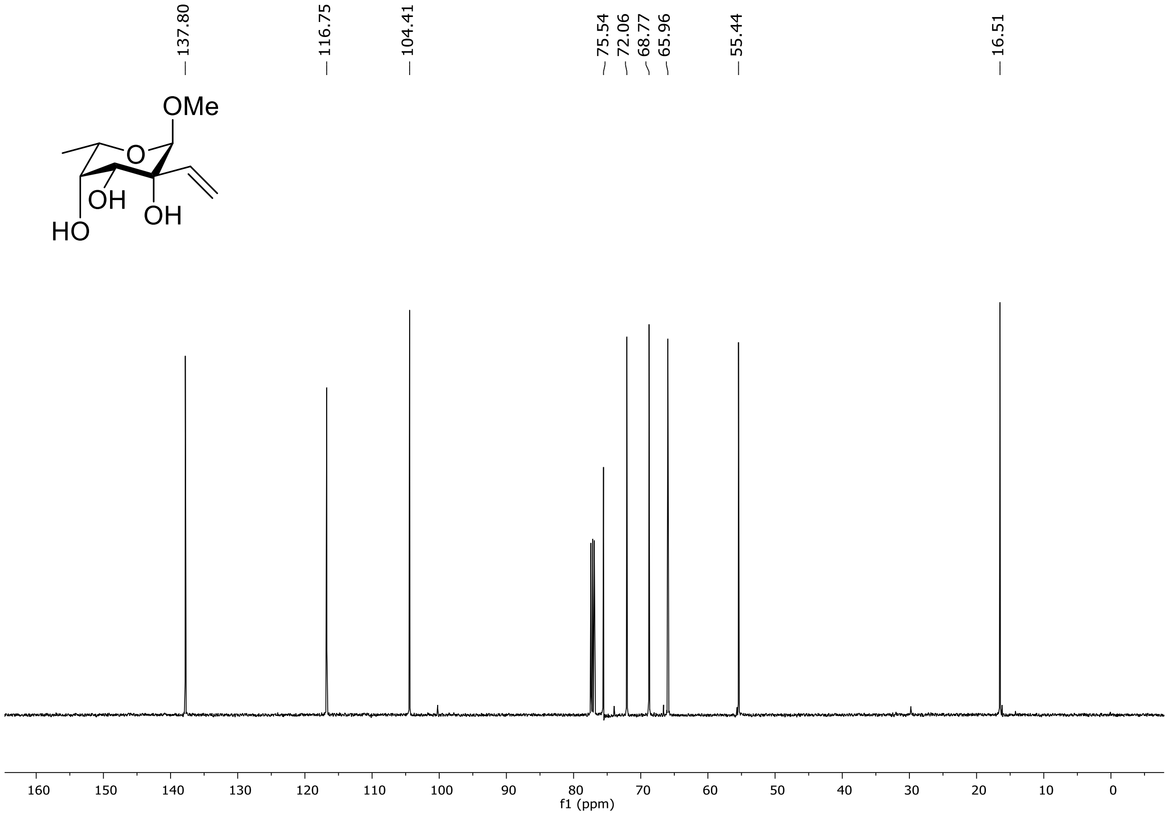

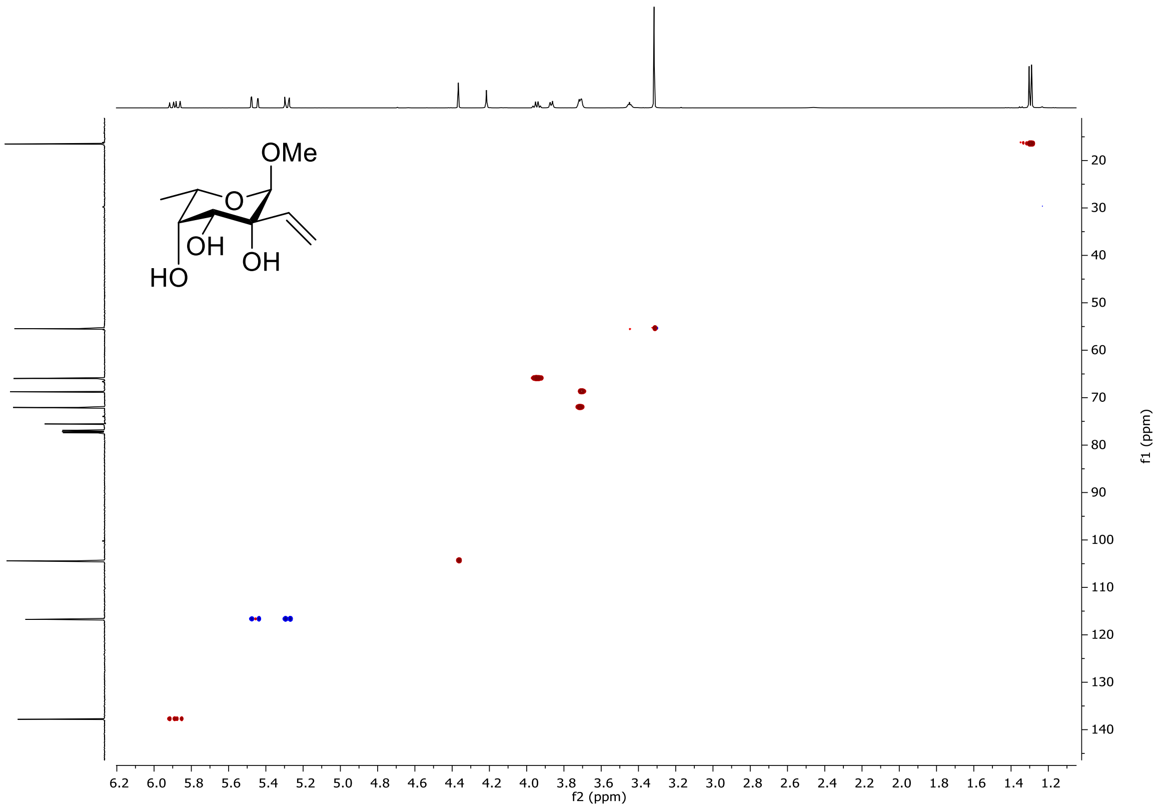
OMe
O
OH
OH
HO
f1 (ppm)
f2 (ppm)
6.2 6.0 5.8 5.6 5.4 5.2 5.0 4.8 4.6 4.4 4.2 4.0 3.8 3.6 3.4 3.2 3.0 2.8 2.6 2.4 2.2 2.0 1.8 1.6 1.4 1.2
20 30 40 50 60 70 80 90 100 110 120 130 140

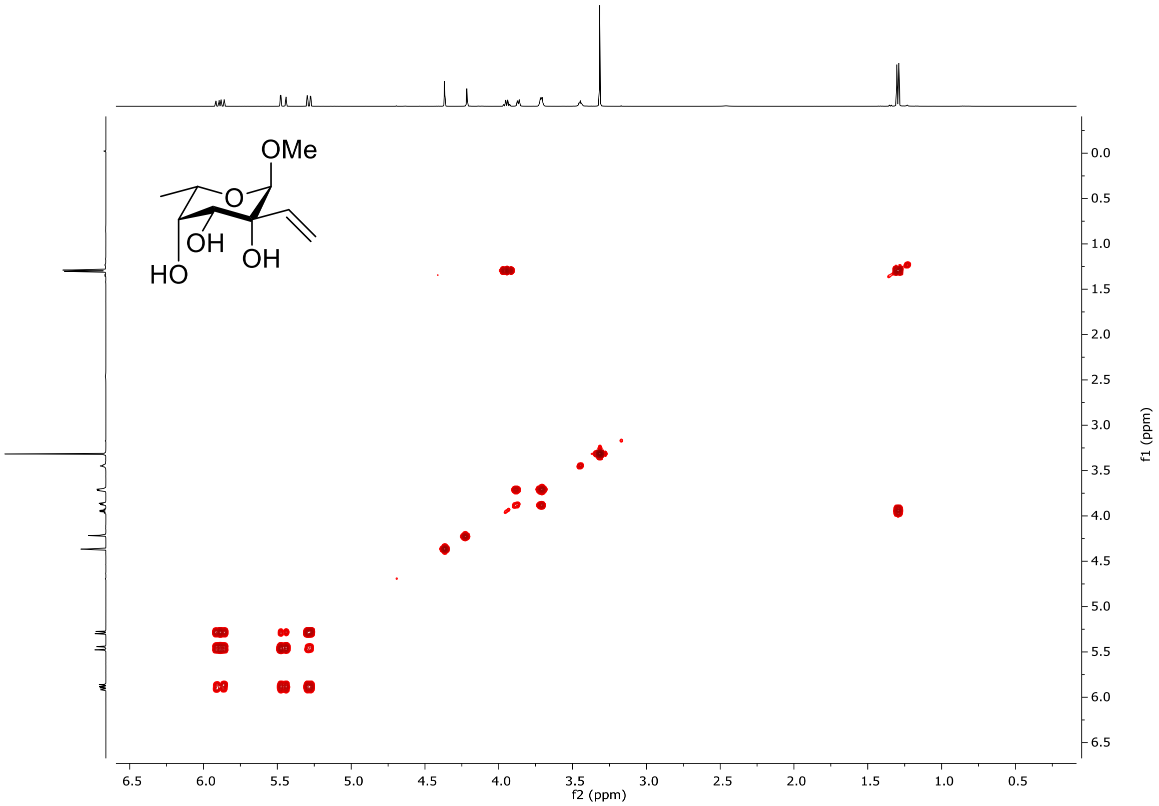
OMe
O
OH
OH
HO
f1 (ppm)
f2 (ppm)
6.5 6.0 5.5 5.0 4.5 4.0 3.5 3.0 2.5 2.0 1.5 1.0 0.5
0.0 0.5 1.0 1.5 2.0 2.5 3.0 3.5 4.0 4.5 5.0 5.5 6.0 6.5

Methyl 2-*C*-allyl-α-L-fucopyranoside (9)

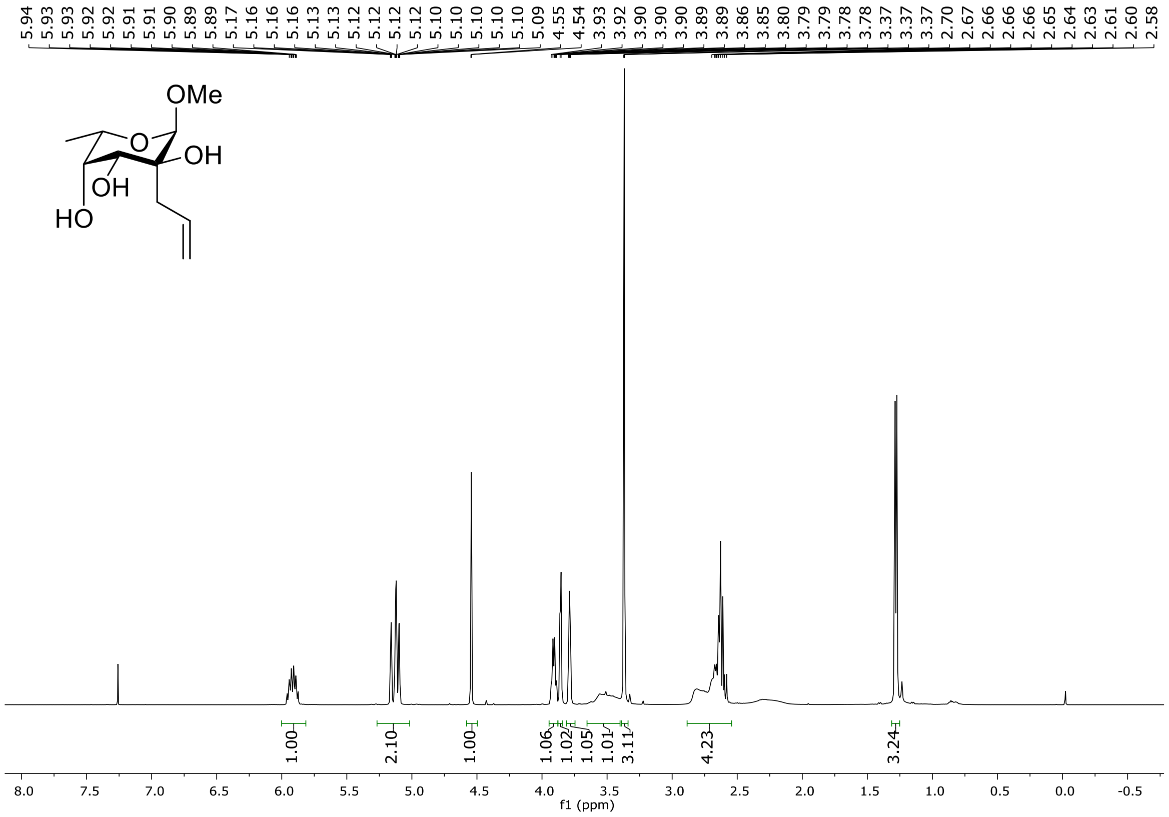

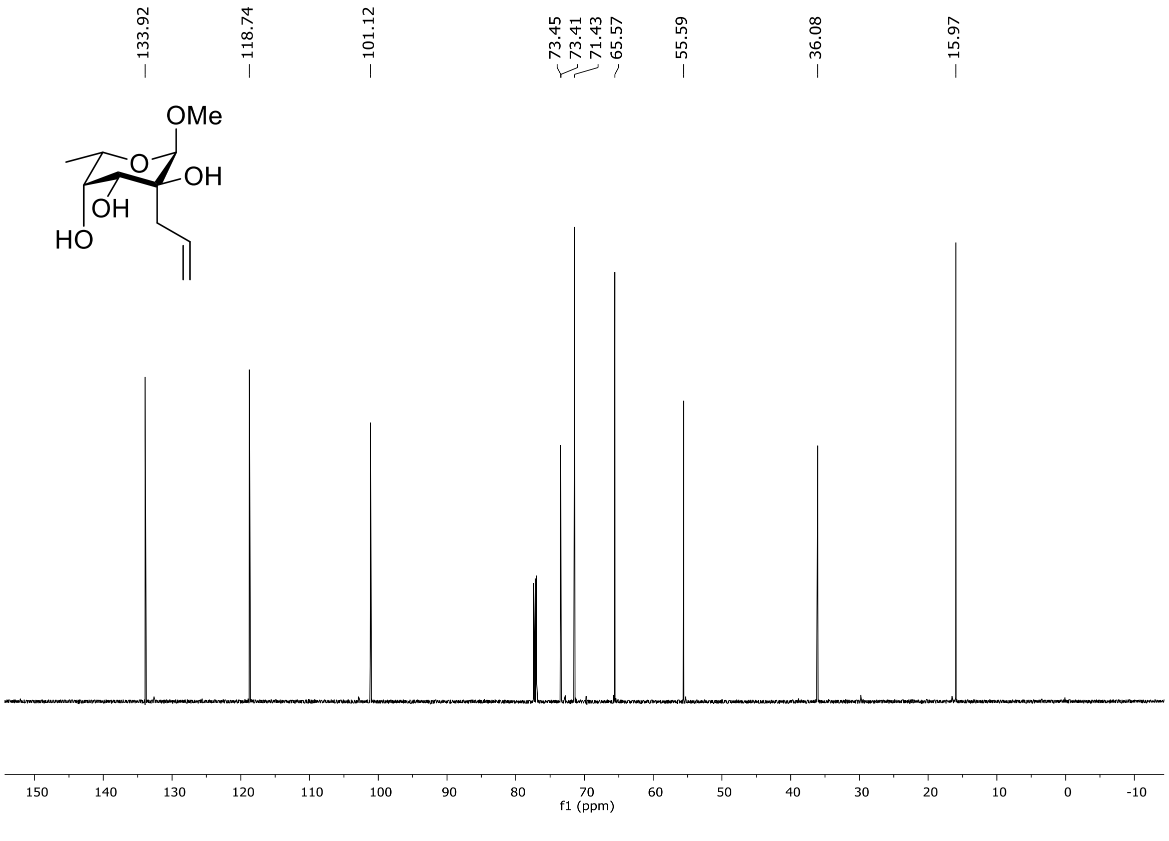

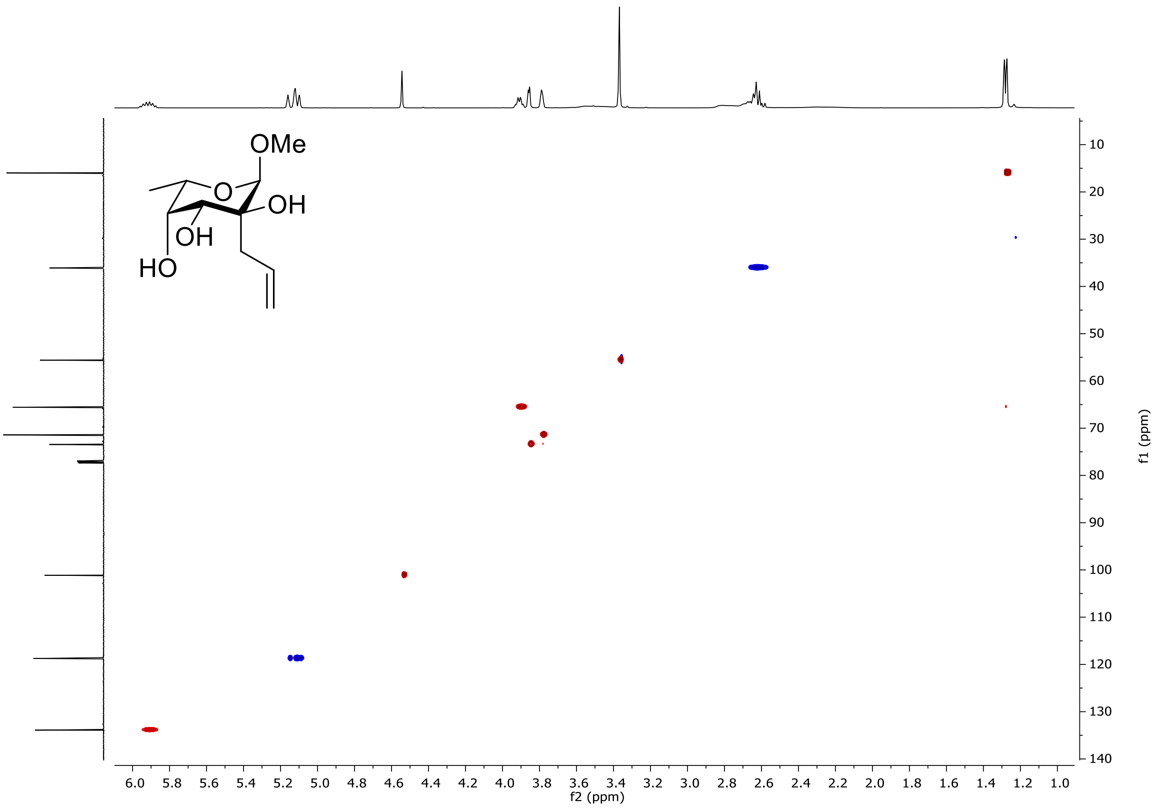
OMe
OH
OH
HO
6.0 5.8 5.6 5.4 5.2 5.0 4.8 4.6 4.4 4.2 4.0 3.8 3.6 3.4 3.2 3.0 2.8 2.6 2.4 2.2 2.0 1.8 1.6 1.4 1.2 1.0
f2 (ppm)
10 20 30 40 50 60 70 80 90 100 110 120 130 140
f1 (ppm)

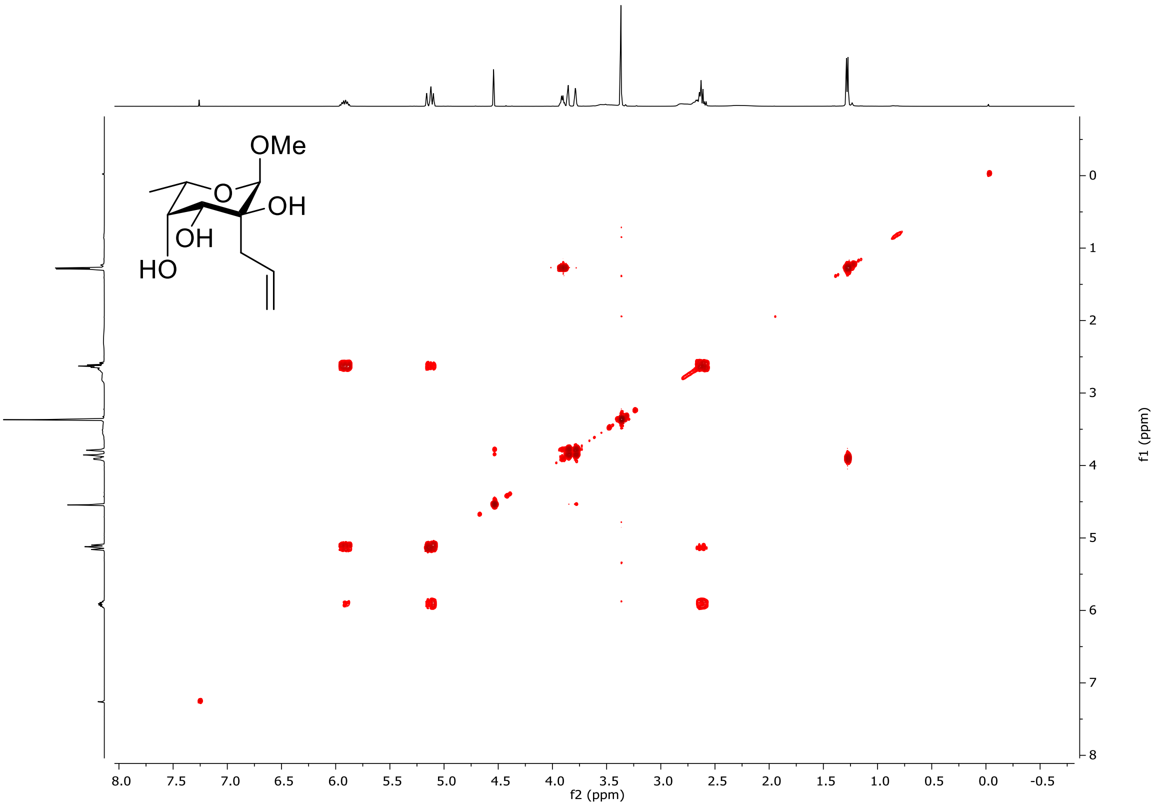
OMe
OH
OH
HO
8.0 7.5 7.0 6.5 6.0 5.5 5.0 4.5 4.0 3.5 3.0 2.5 2.0 1.5 1.0 0.5 0.0 -0.5
f2 (ppm)
0 1 2 3 4 5 6 7 8
f1 (ppm)

^{1}H-^{1}H NOESY

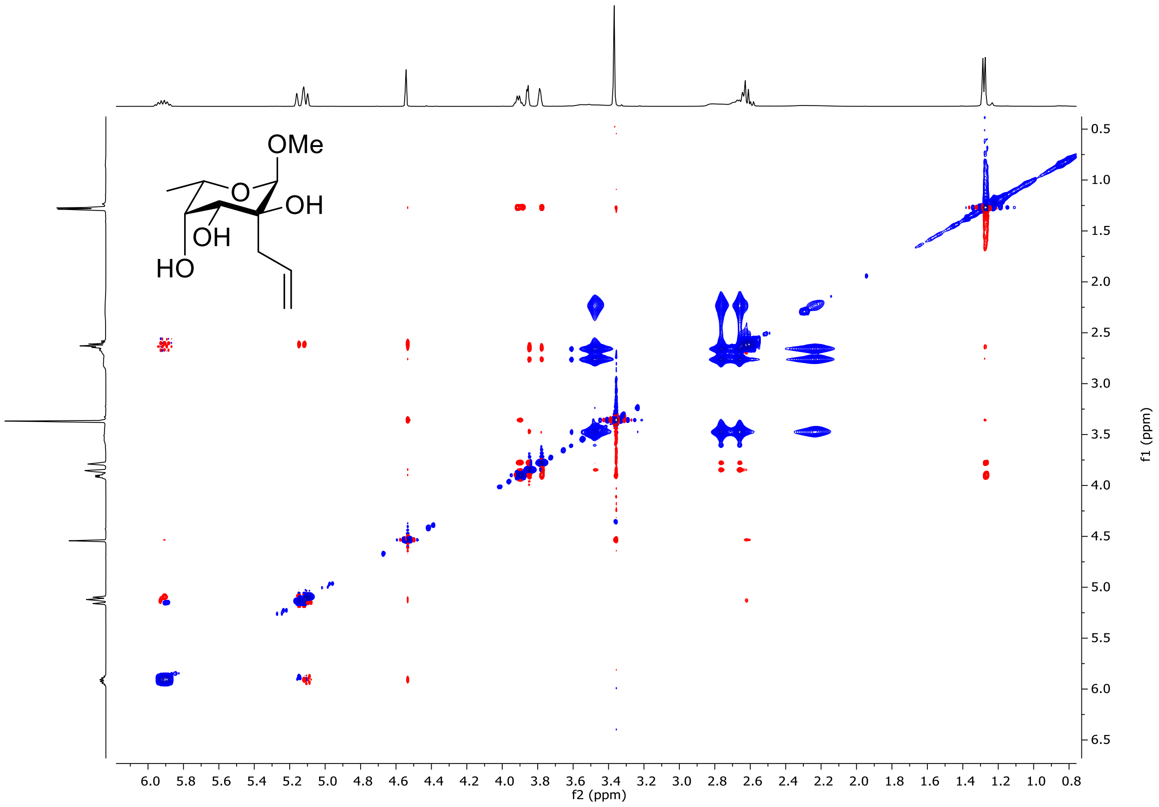

Methyl 2-*C*-allyl-6-deoxy-α-L-talopyranoside (11)

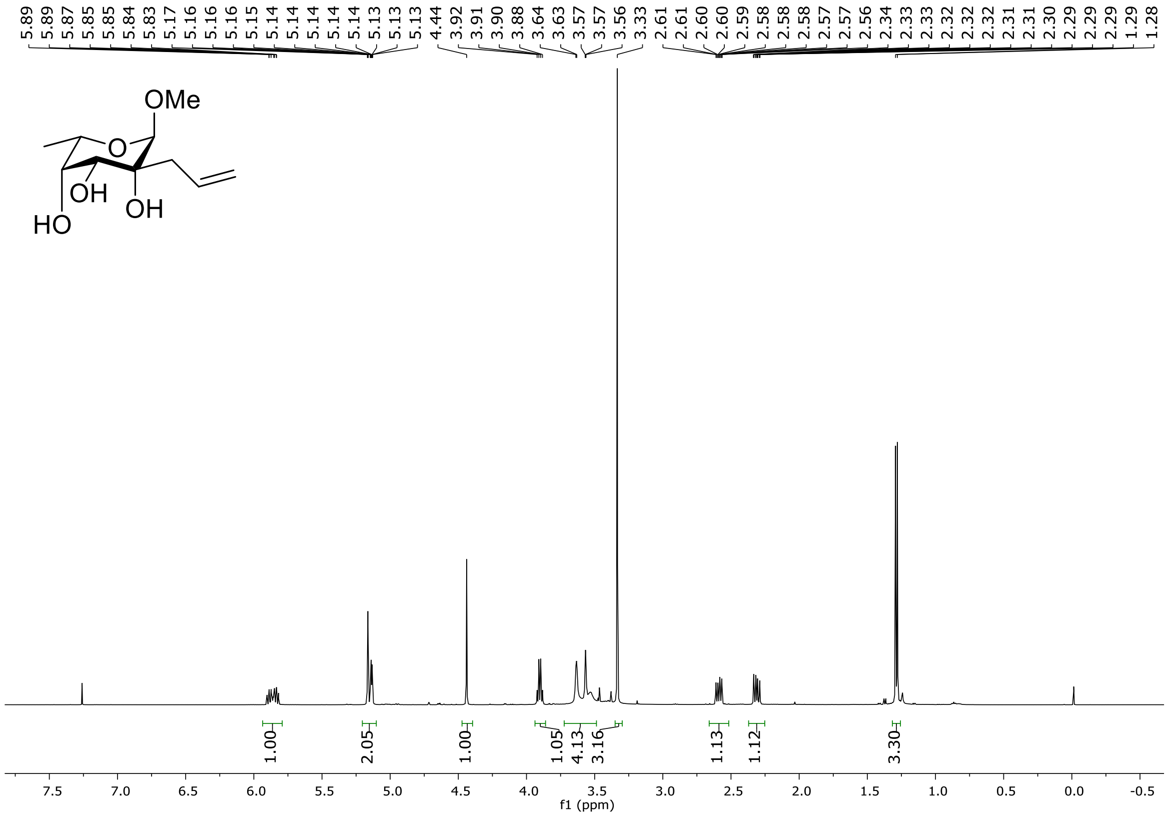

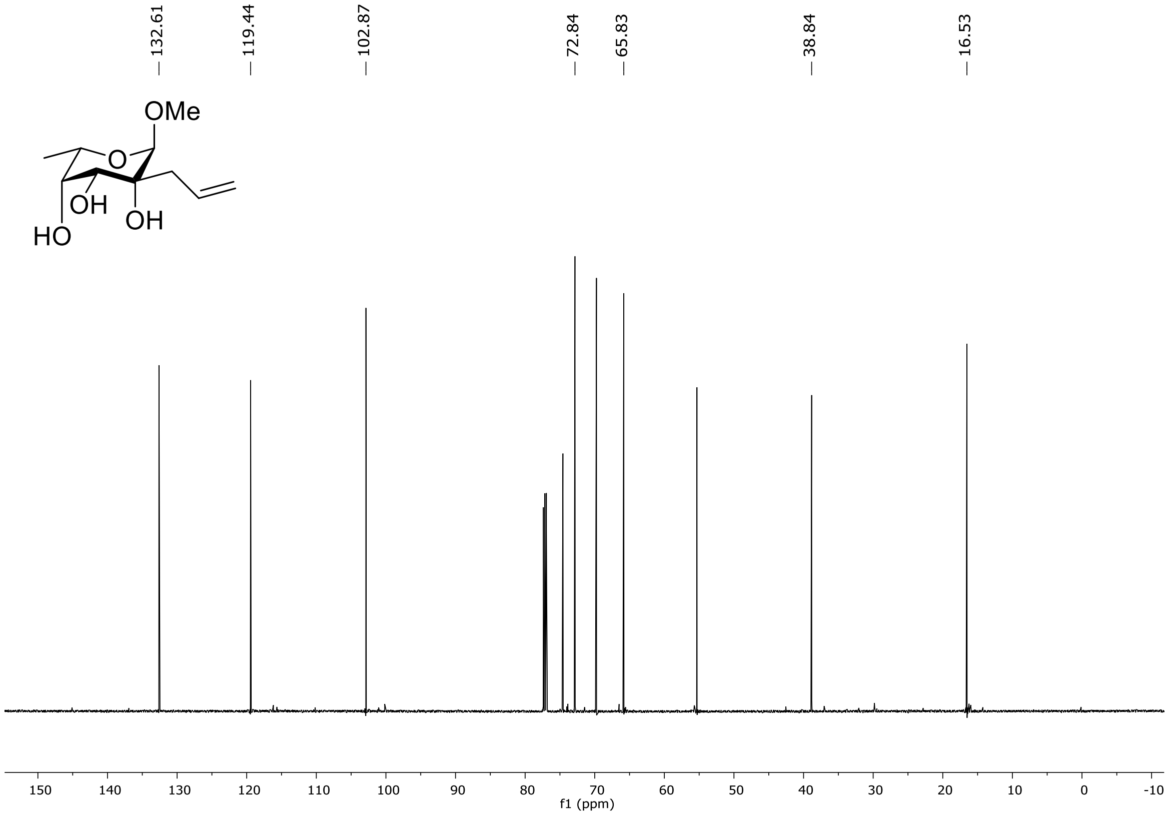

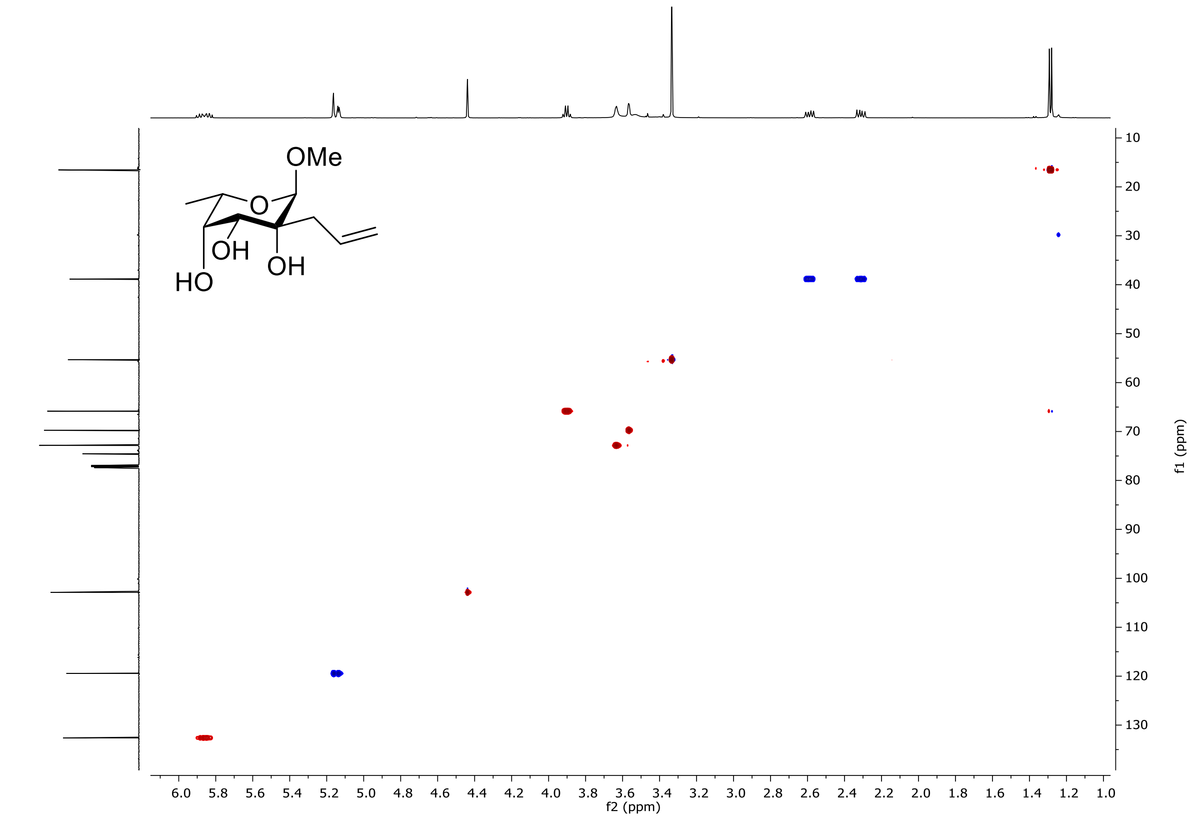
OMe
O
OH
OH
HO
f2 (ppm)
f1 (ppm)

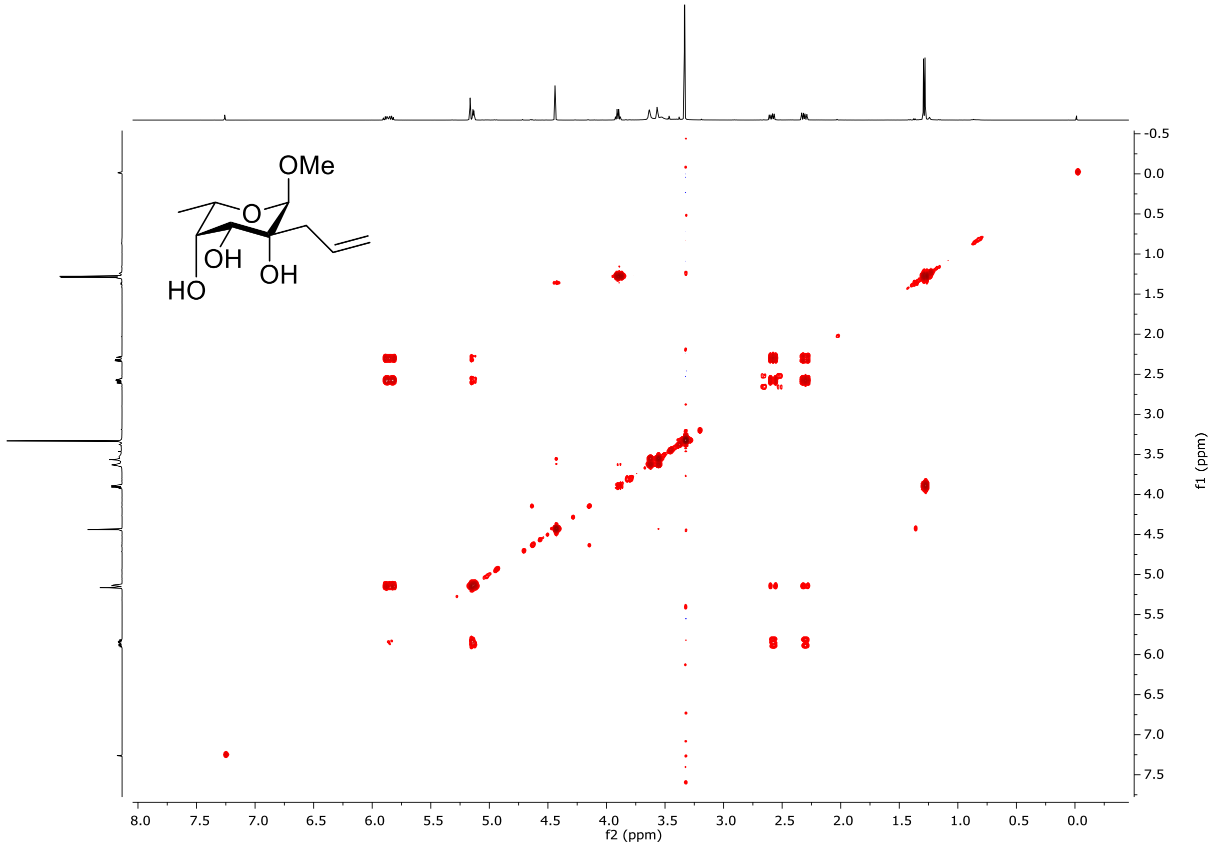
OMe
O
OH
OH
HO
f2 (ppm)
f1 (ppm)

^{1}H-^{1}H NOESY

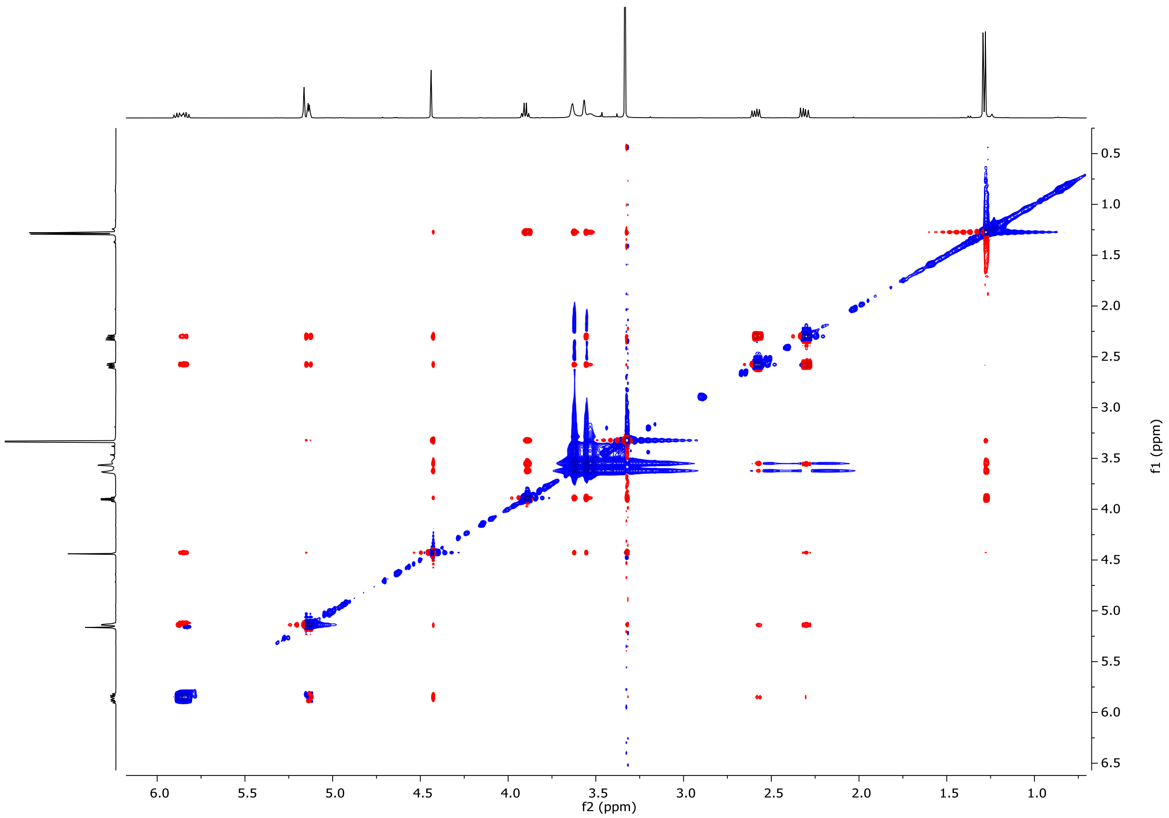

Methyl 2-*C*-[2-acetylthioethyl]-α-L-fucopyranoside (12)

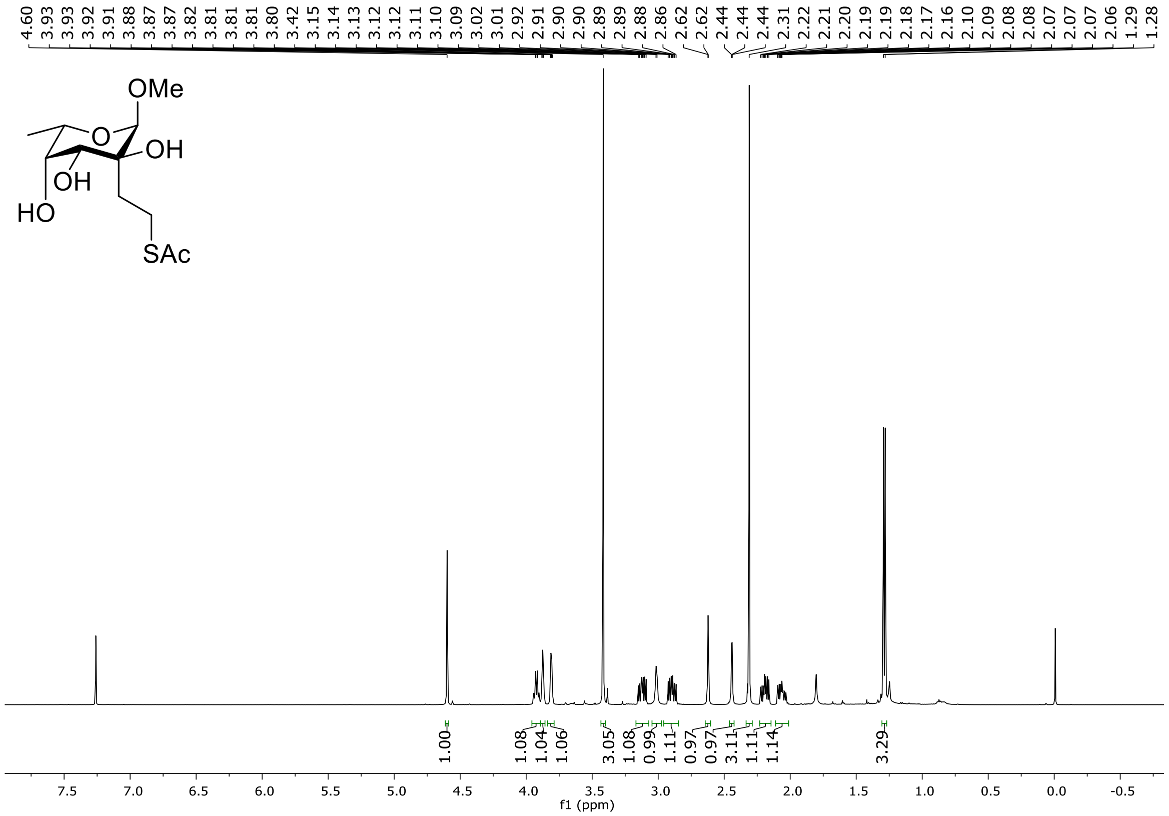

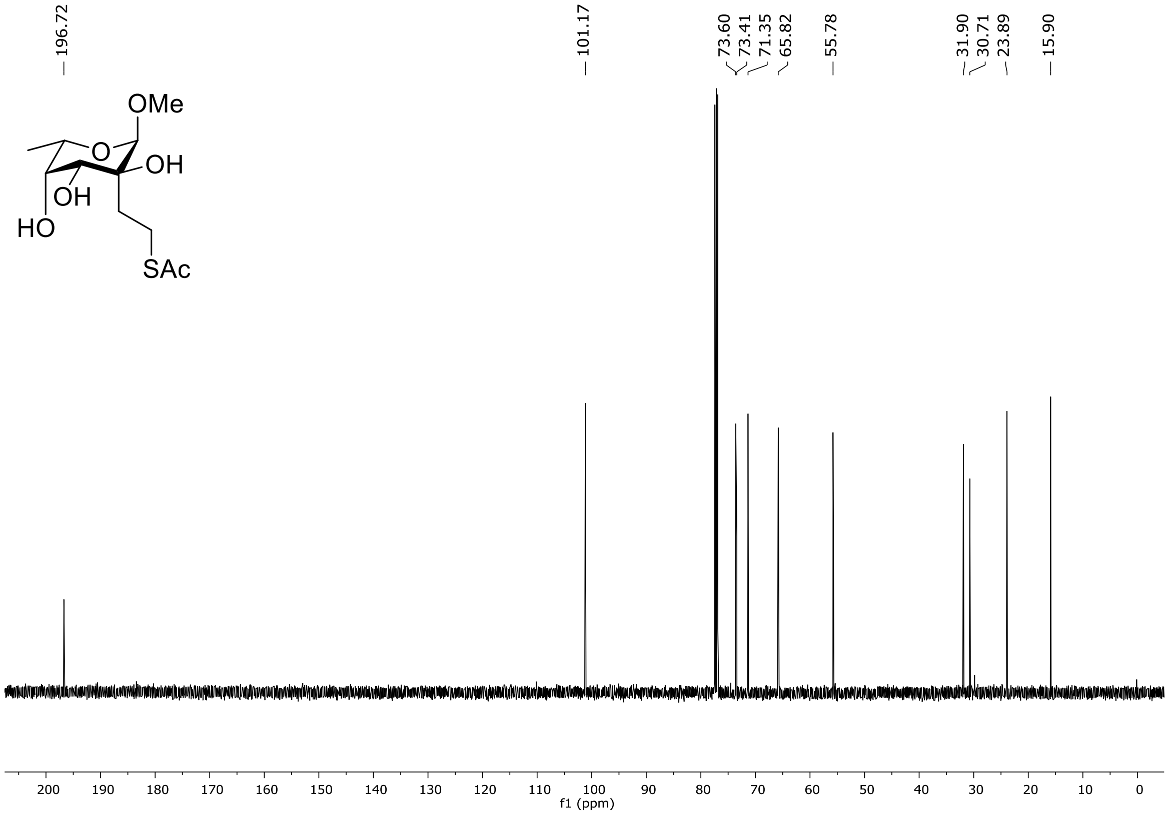

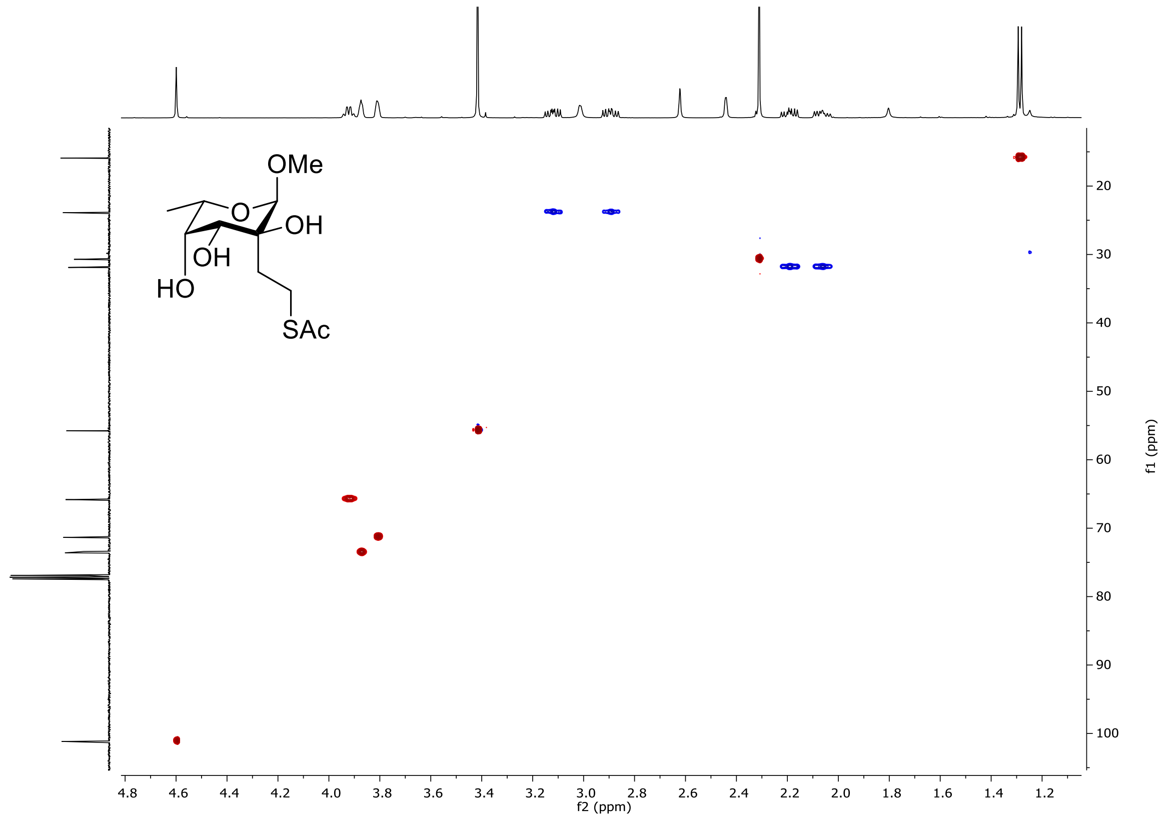

OMe
O
OH
OH
HO
SAc
f2 (ppm)
f1 (ppm)

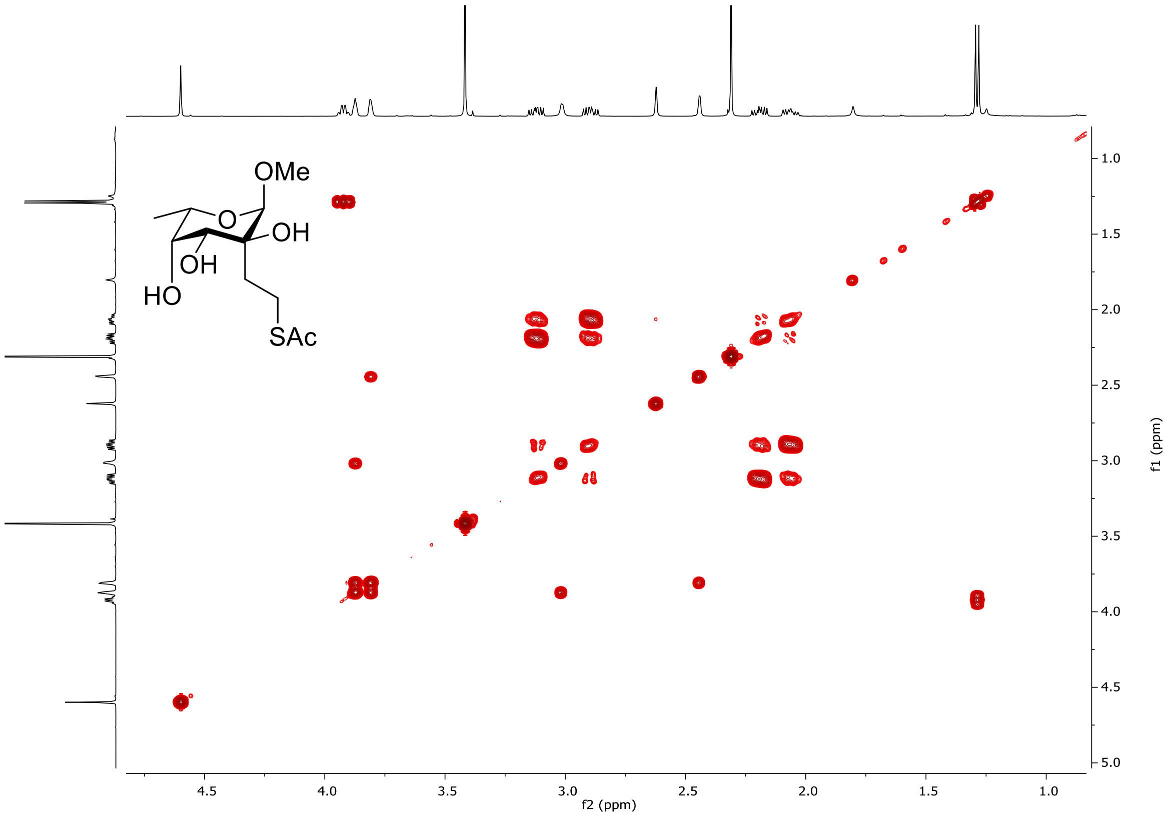

OMe
O
OH
OH
HO
SAc
f2 (ppm)
f1 (ppm)

Methyl 2-*C*-[3-acetylthiopropyl]-α-L-fucopyranoside (13)

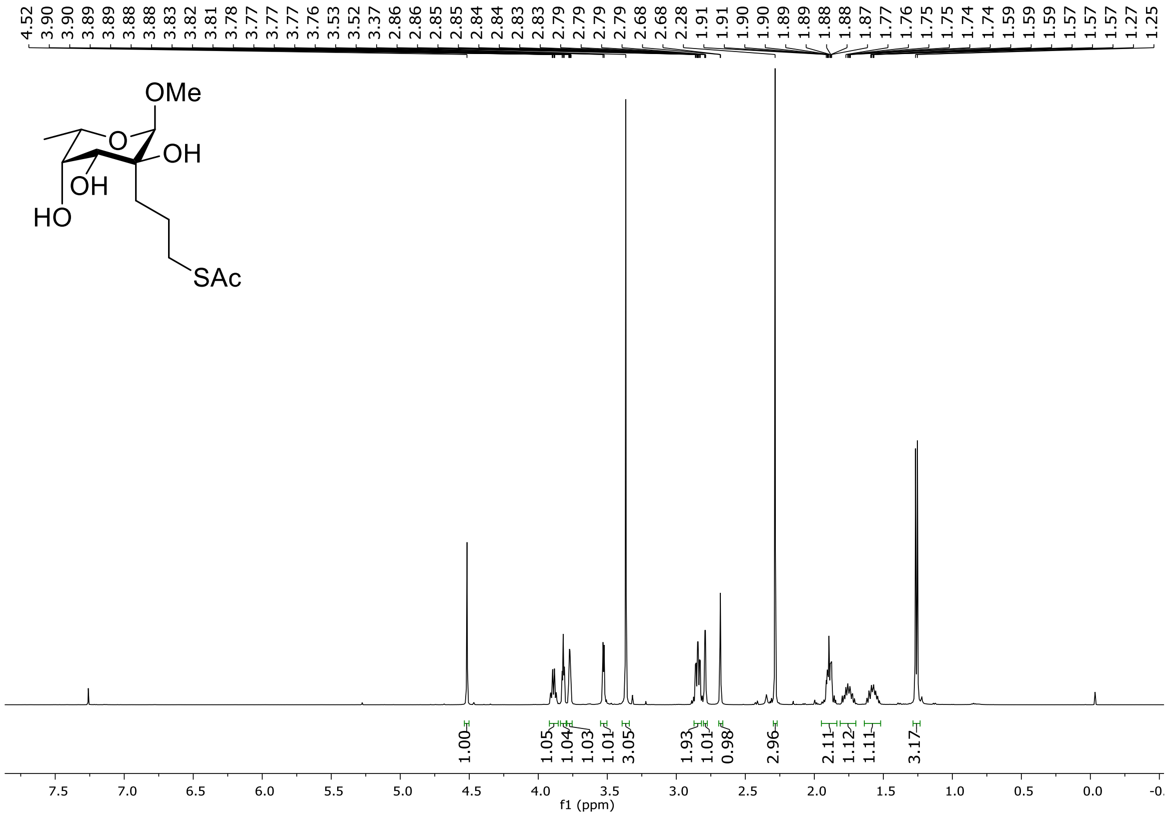

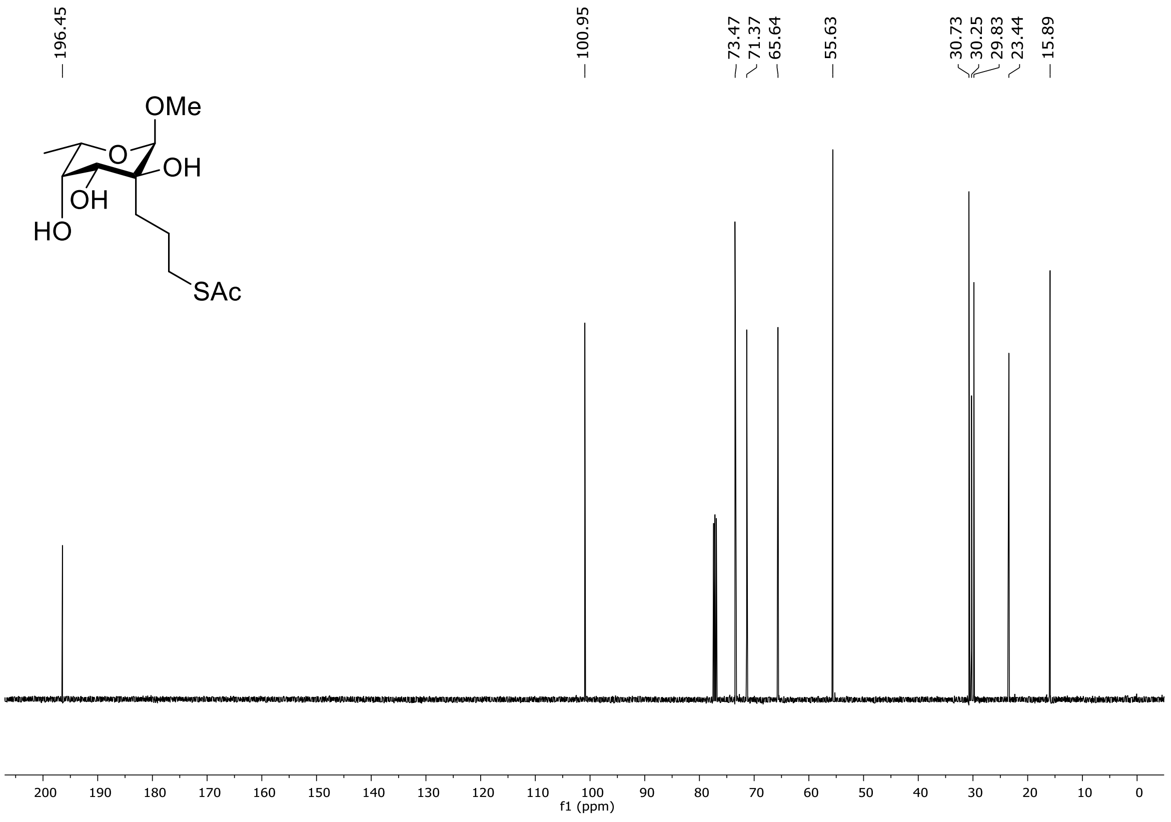

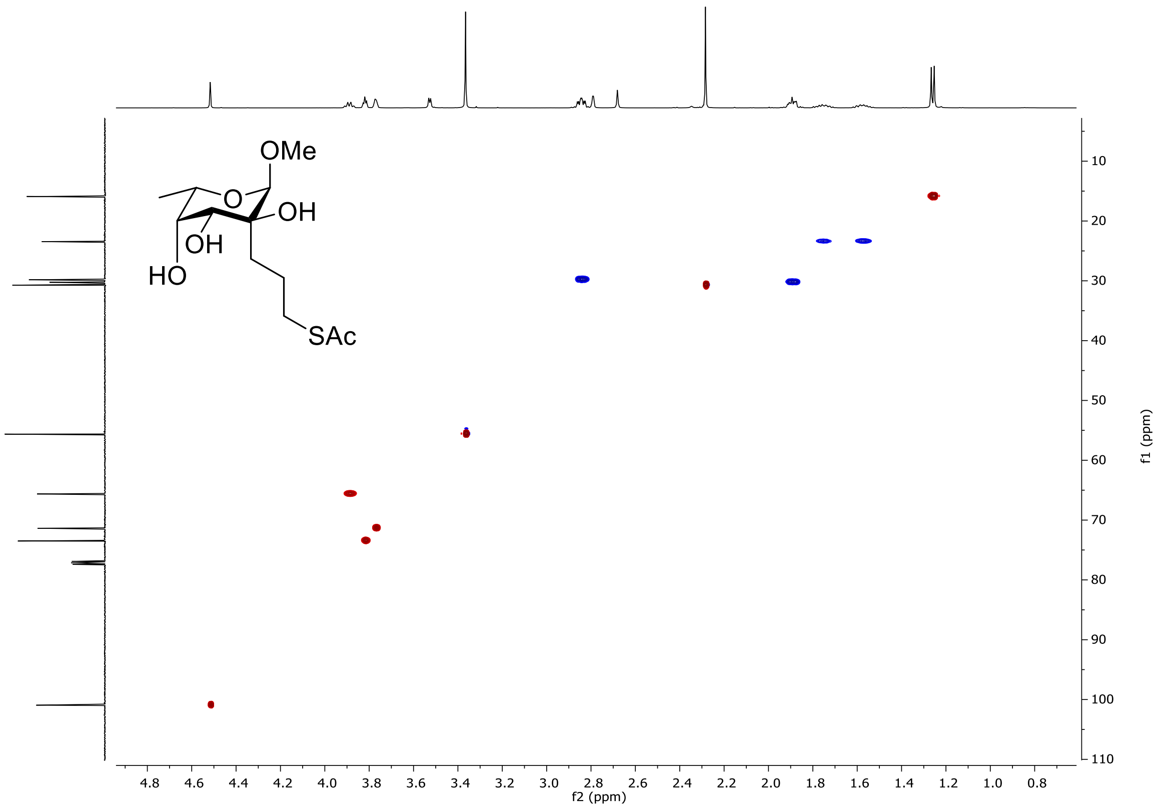

OMe
O
OH
OH
HO
SAc
f2 (ppm)
f1 (ppm)

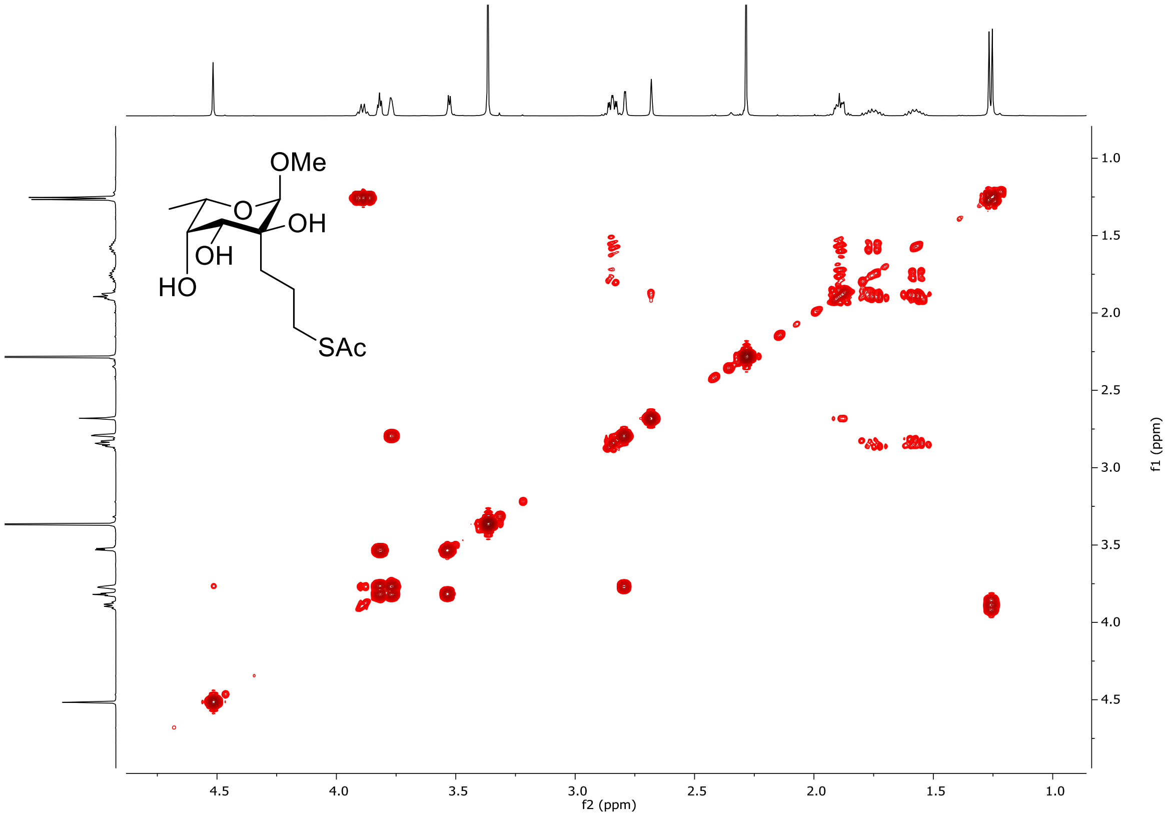

OMe
O
OH
OH
HO
SAc
f2 (ppm)
f1 (ppm)

Methyl 2-*C*-[2-acetylthioethyl]-6-deoxy-α-L-talopyranoside (14)

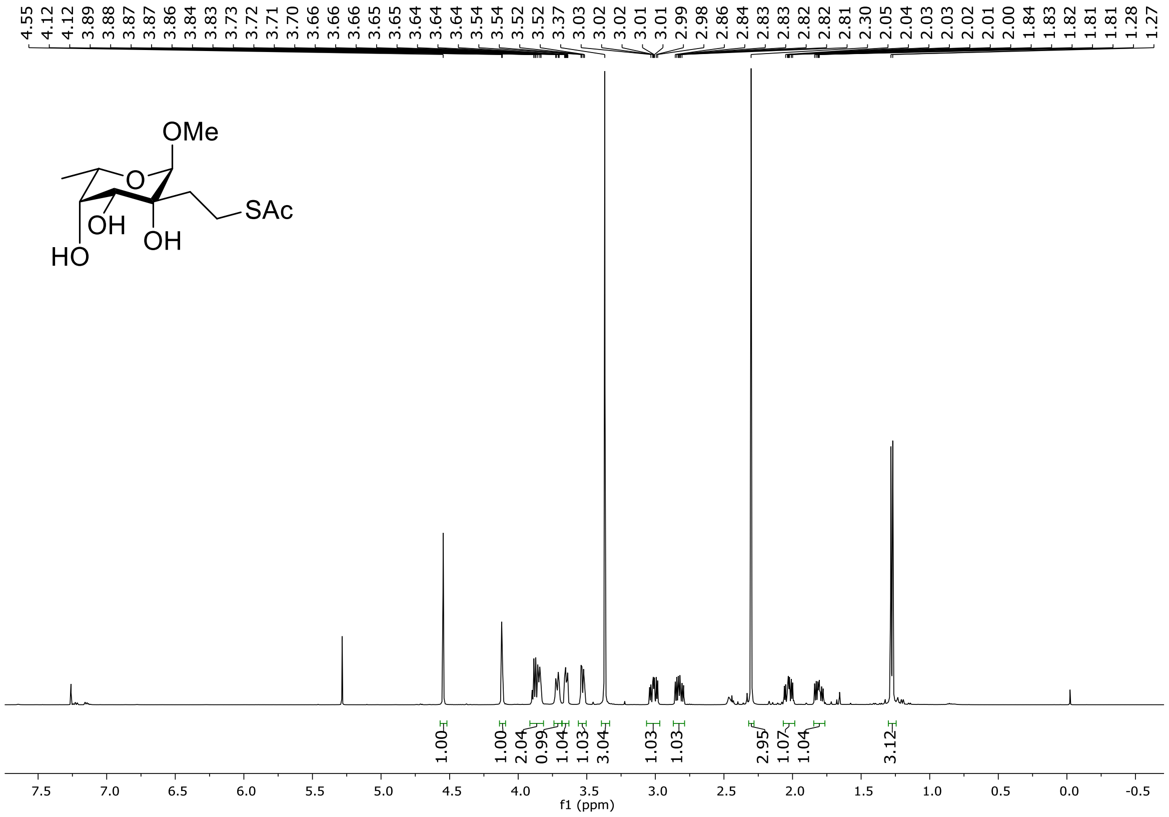

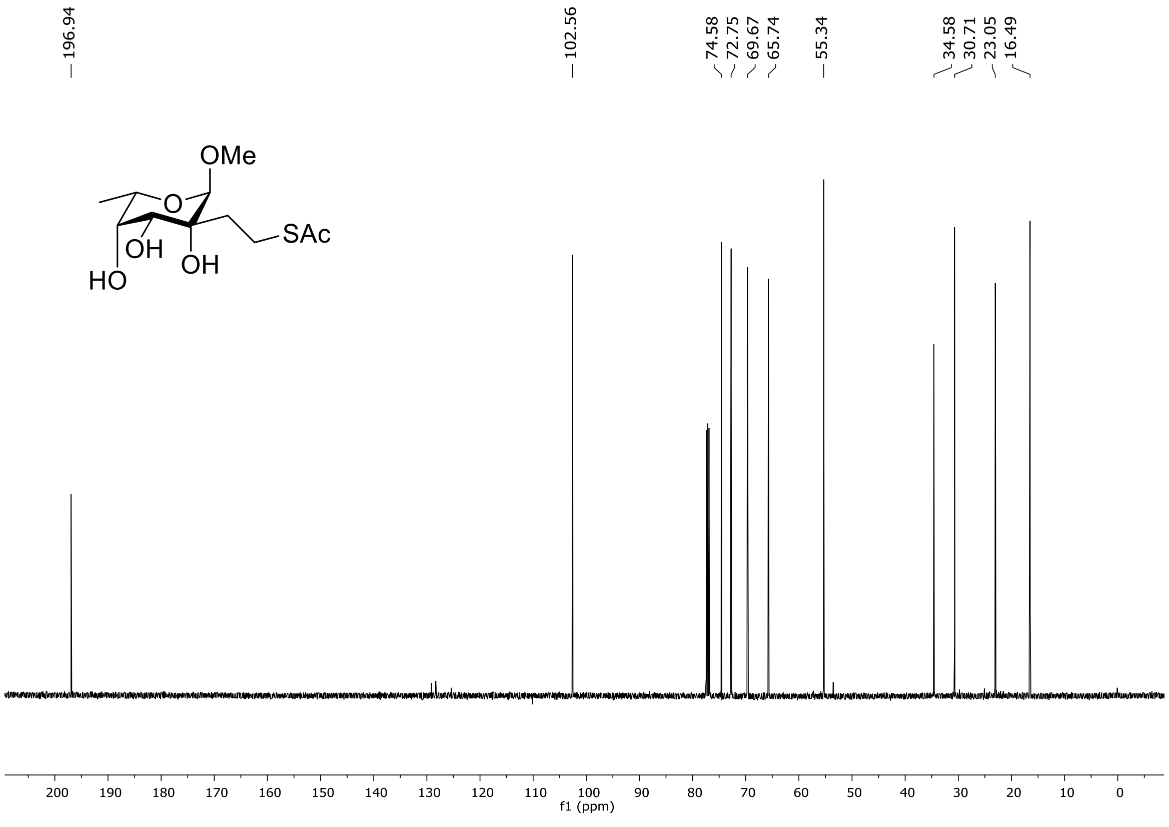

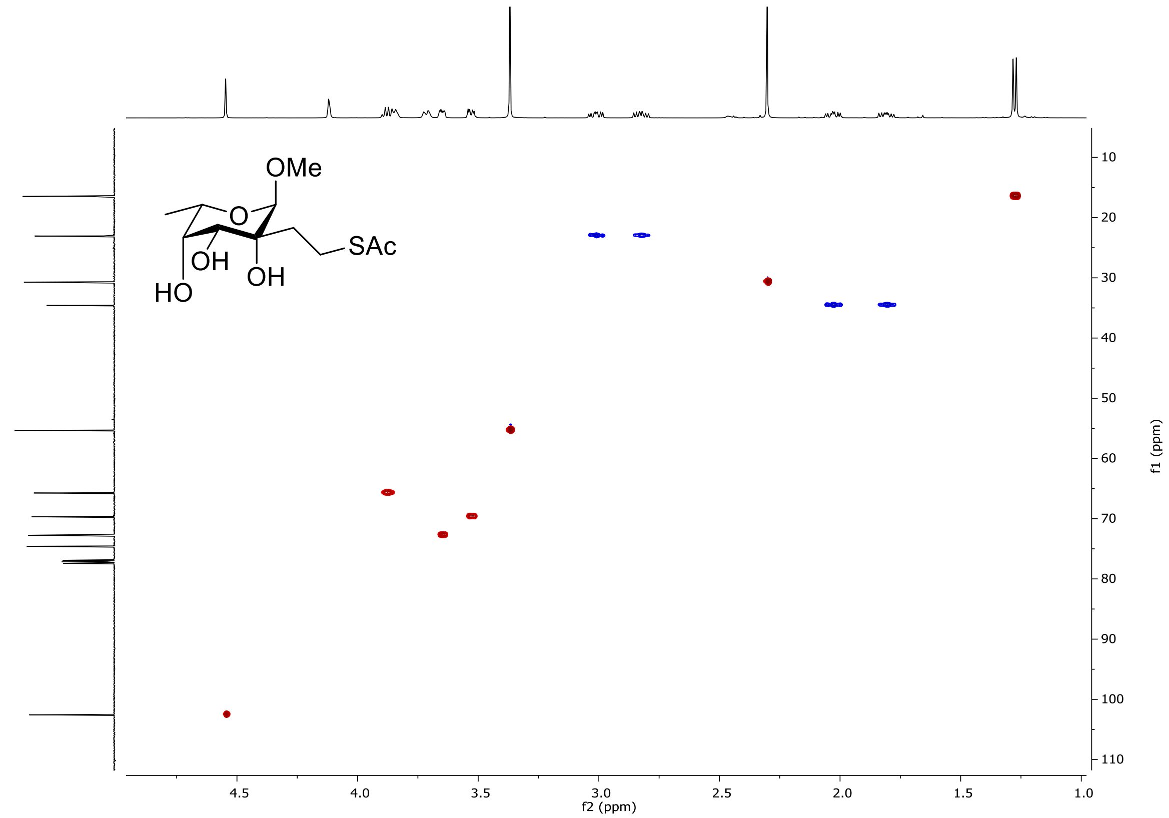
OMe
O
SAc
OH
OH
HO
f1 (ppm)
f2 (ppm)
10
20
30
40
50
60
70
80
90
100
110
4.5
4.0
3.5
3.0
2.5
2.0
1.5
1.0

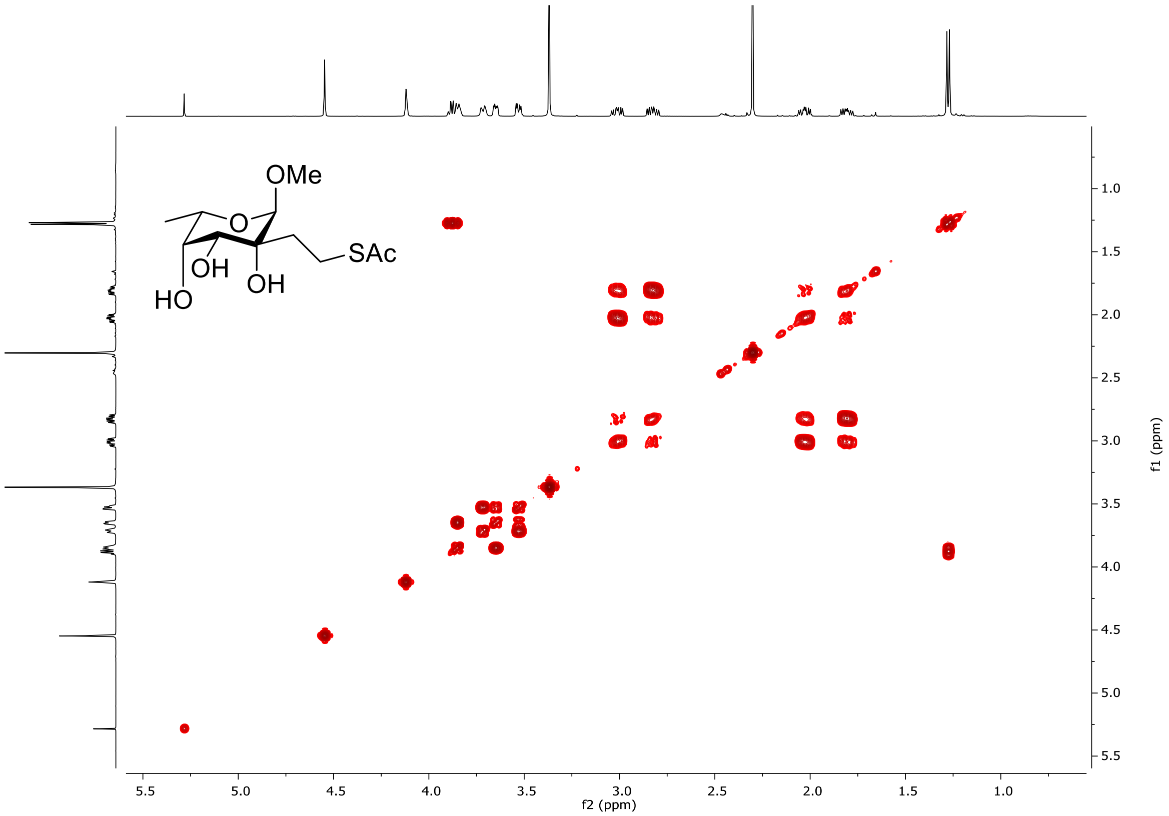
OMe
O
SAc
OH
OH
HO
f1 (ppm)
f2 (ppm)
1.0
1.5
2.0
2.5
3.0
3.5
4.0
4.5
5.0
5.5

Methyl 2-*C*-[3-acetylthiopropyl]-6-deoxy-α-L-talopyranoside (15)

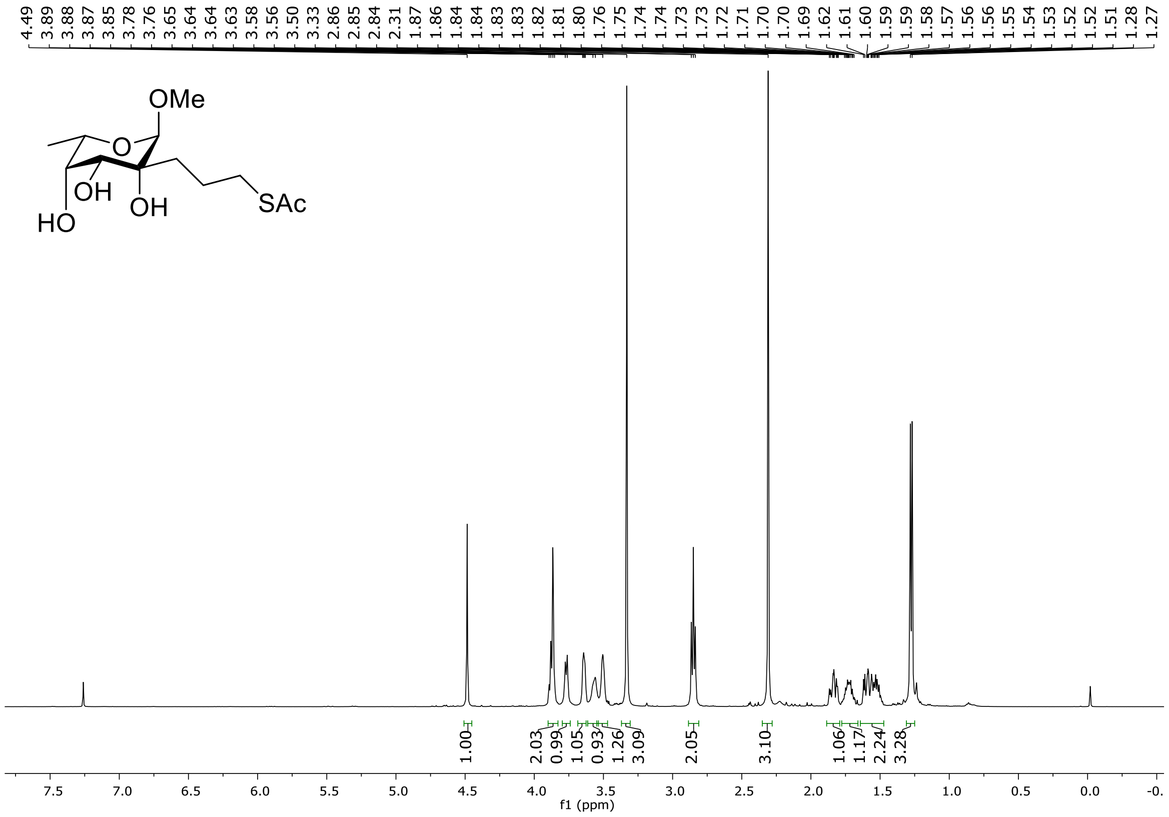

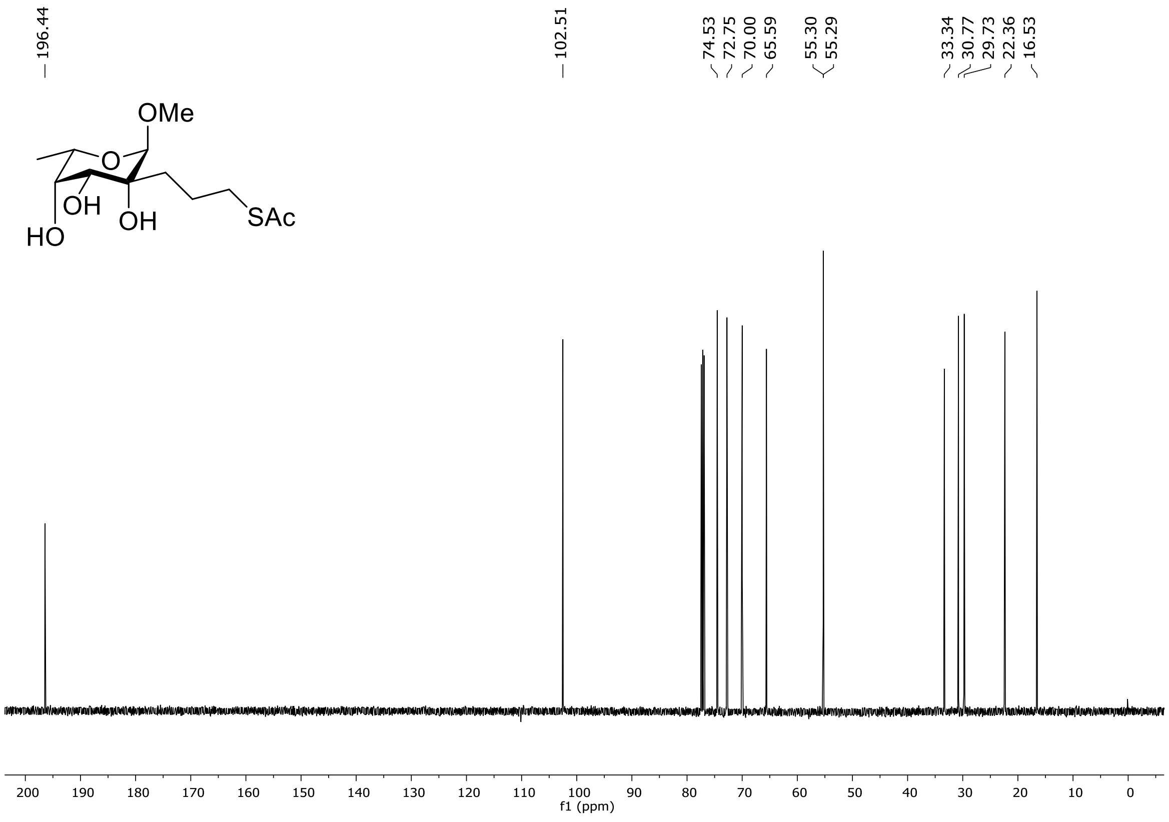

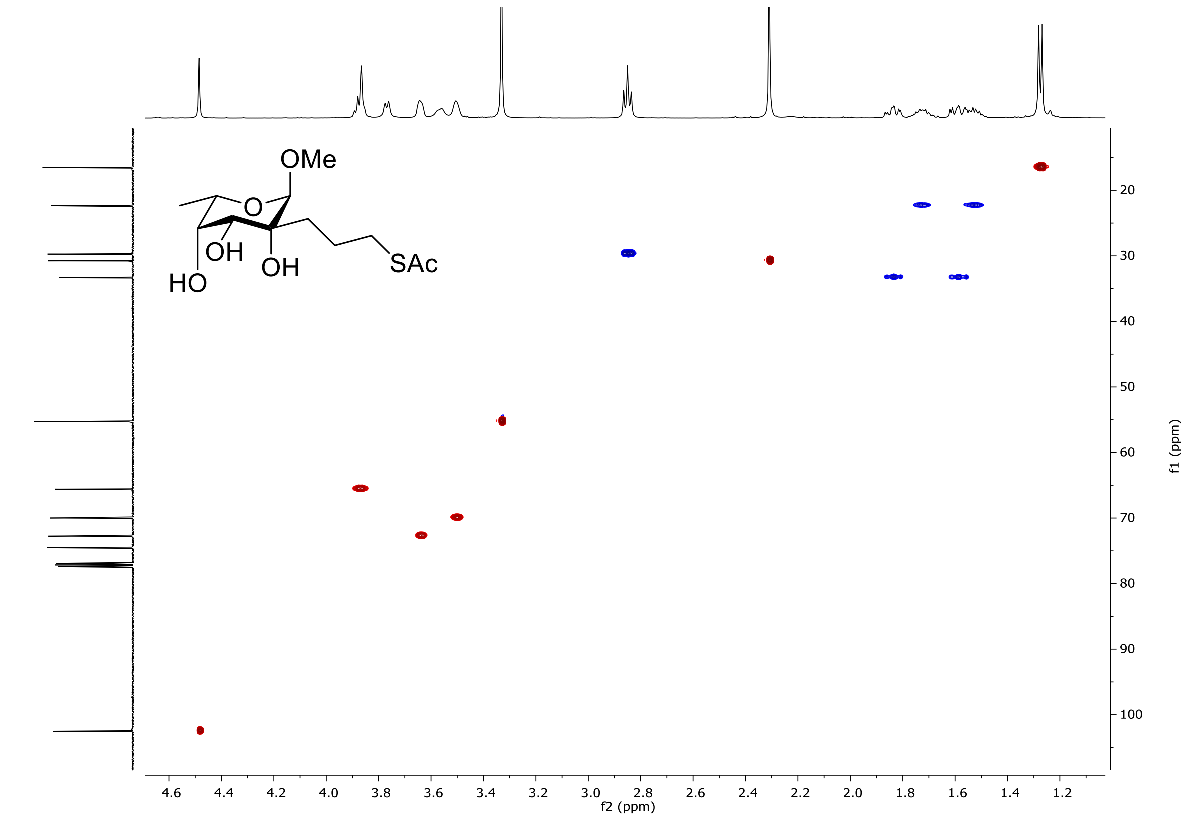
OMe
O
OH
OH
HO
SAc
4.6 4.4 4.2 4.0 3.8 3.6 3.4 3.2 3.0 2.8 2.6 2.4 2.2 2.0 1.8 1.6 1.4 1.2
f2 (ppm)
20 30 40 50 60 70 80 90 100
f1 (ppm)

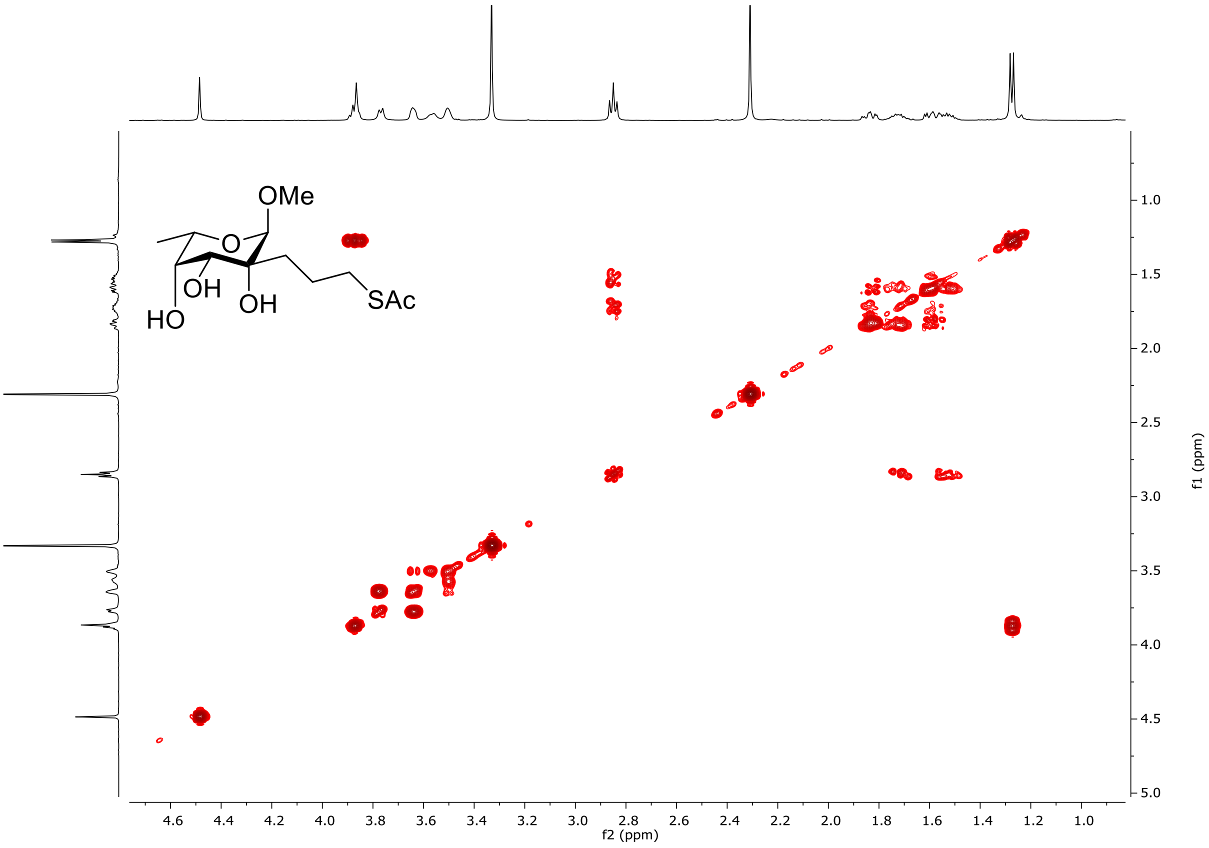
OMe
O
OH
OH
HO
SAc
4.6 4.4 4.2 4.0 3.8 3.6 3.4 3.2 3.0 2.8 2.6 2.4 2.2 2.0 1.8 1.6 1.4 1.2 1.0
f2 (ppm)
1.0 1.5 2.0 2.5 3.0 3.5 4.0 4.5 5.0
f1 (ppm)

Methyl 2-*C*-[2-thioethyl]-α-L-fucopyranoside (1)

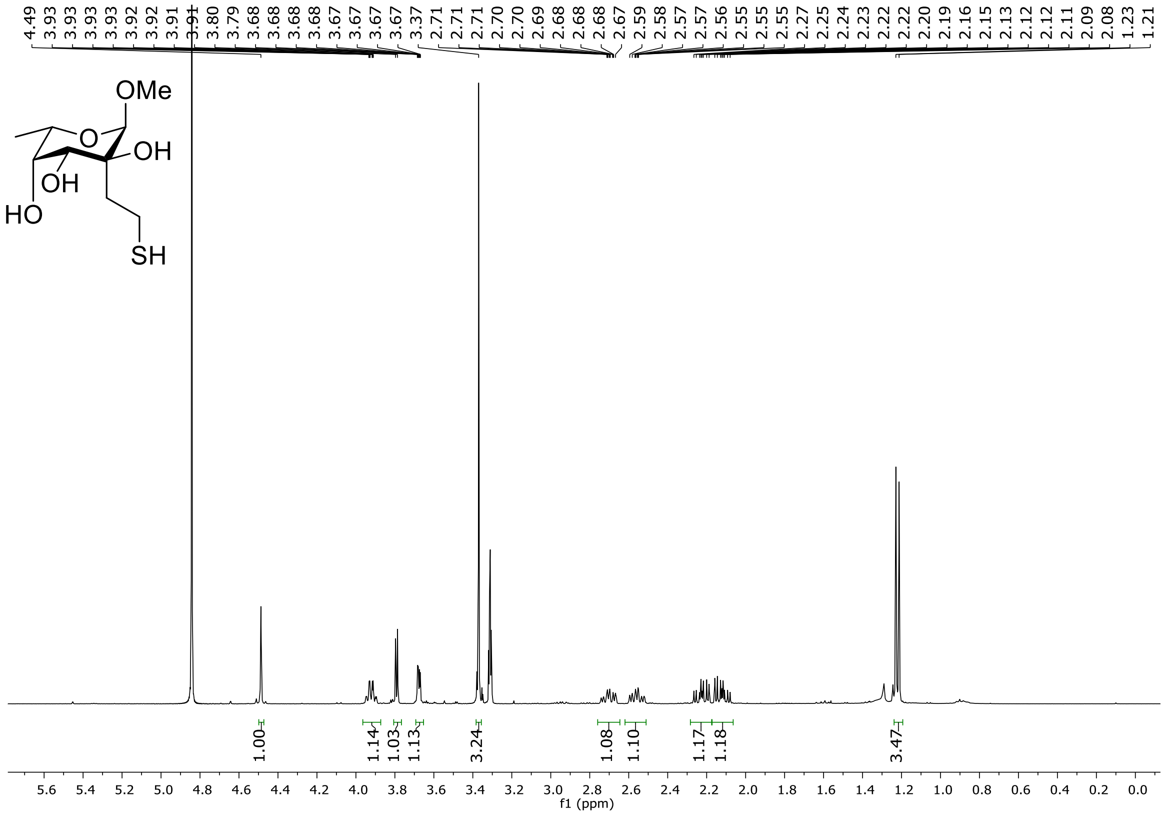

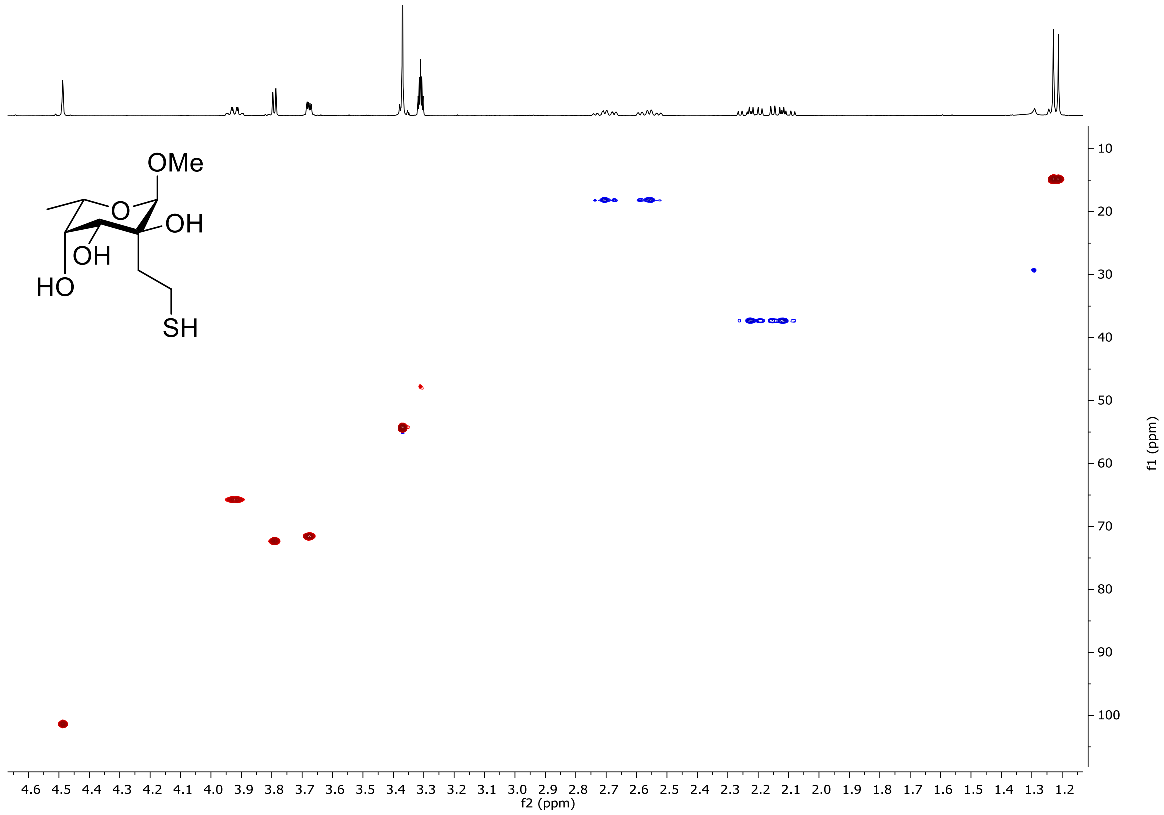

Methyl 2-*C*-[3-thiopropyl]-α-L-fucopyranoside (2)

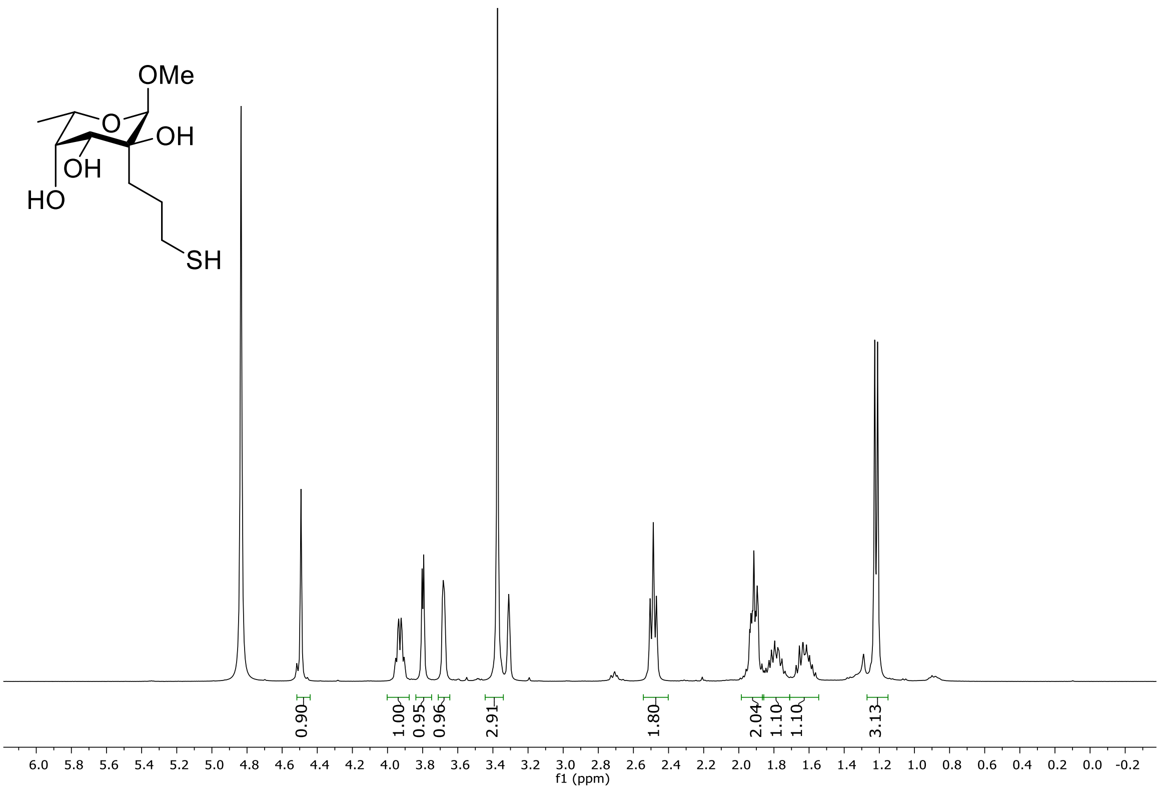

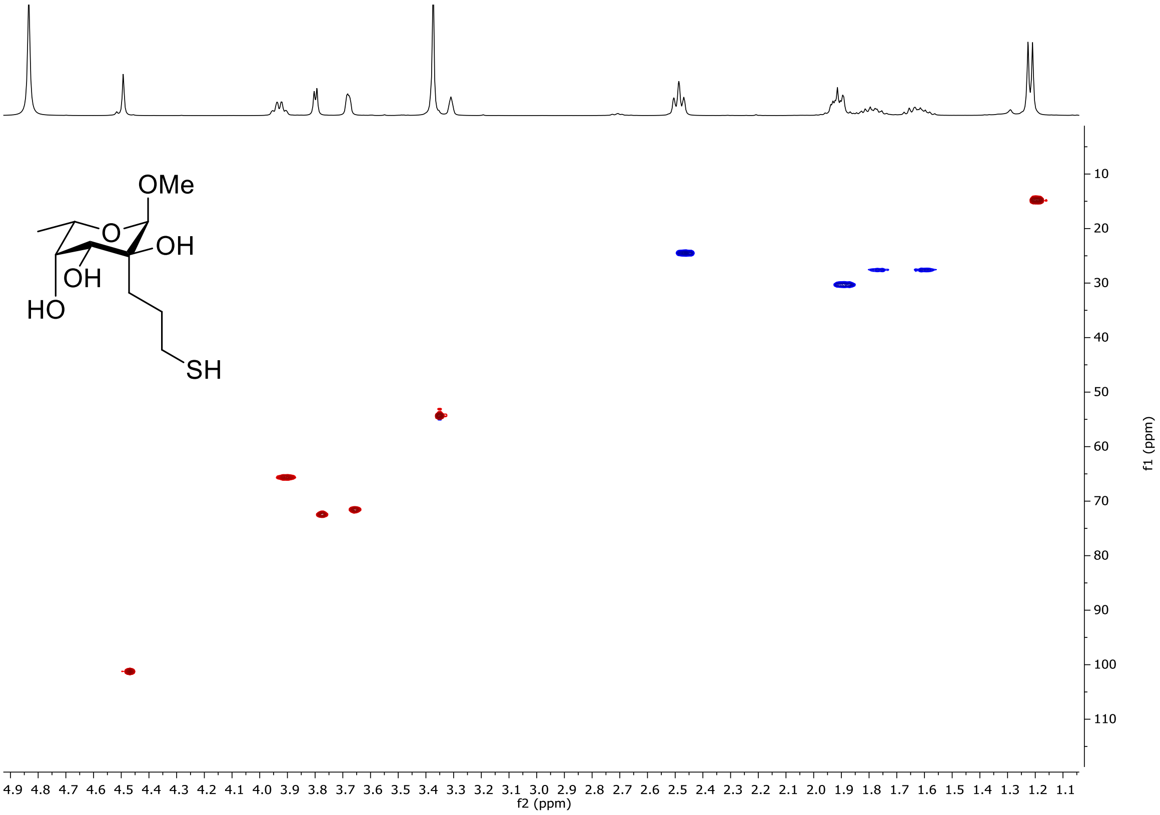

Methyl 6-deoxy-2-*C*-[2-thioethyl]-α-L-talopyranoside (3)

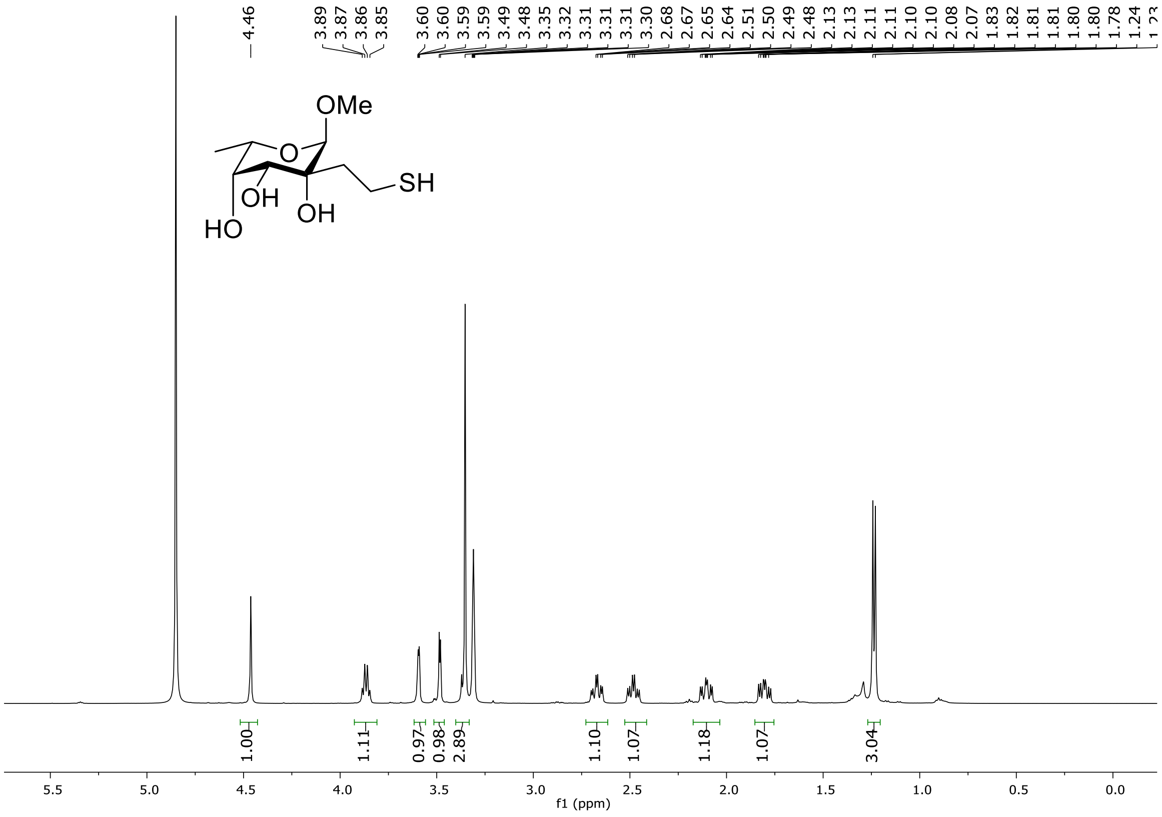

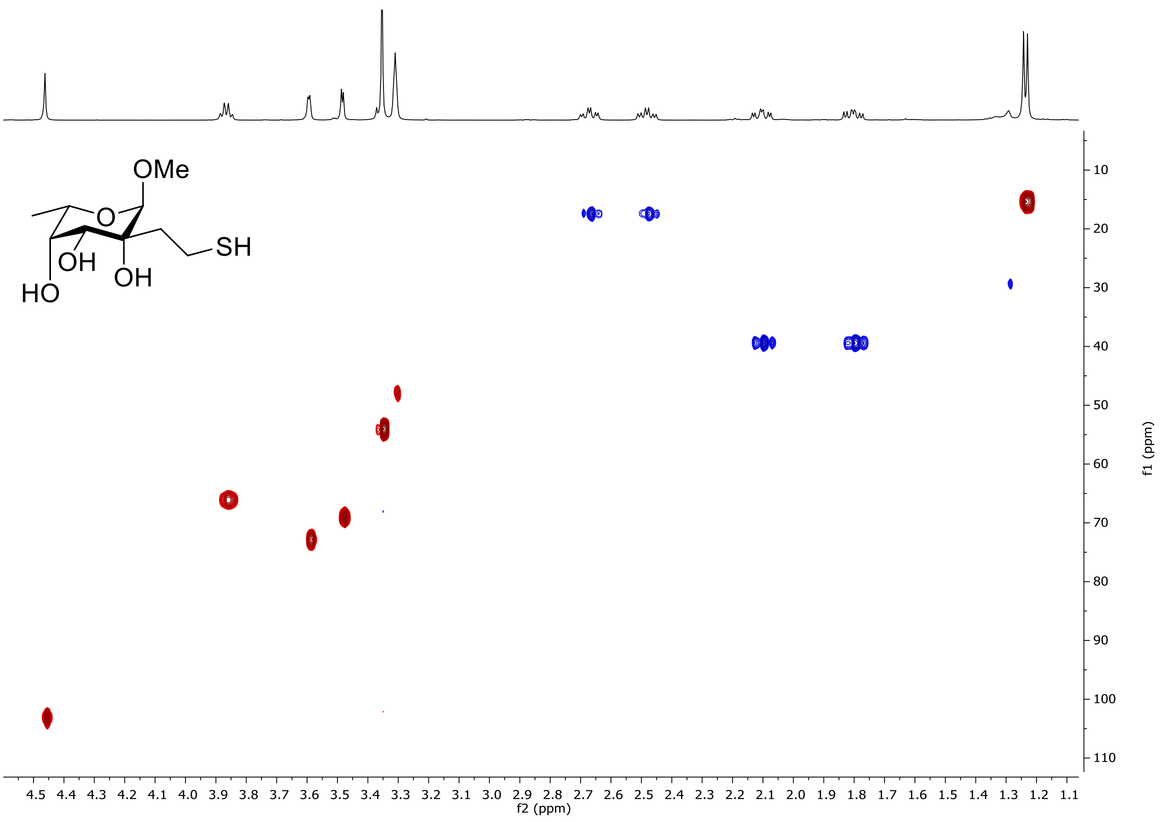

Methyl 6-deoxy-2-*C*-[3-thiopropyl]-α-L-talopyranoside (4)

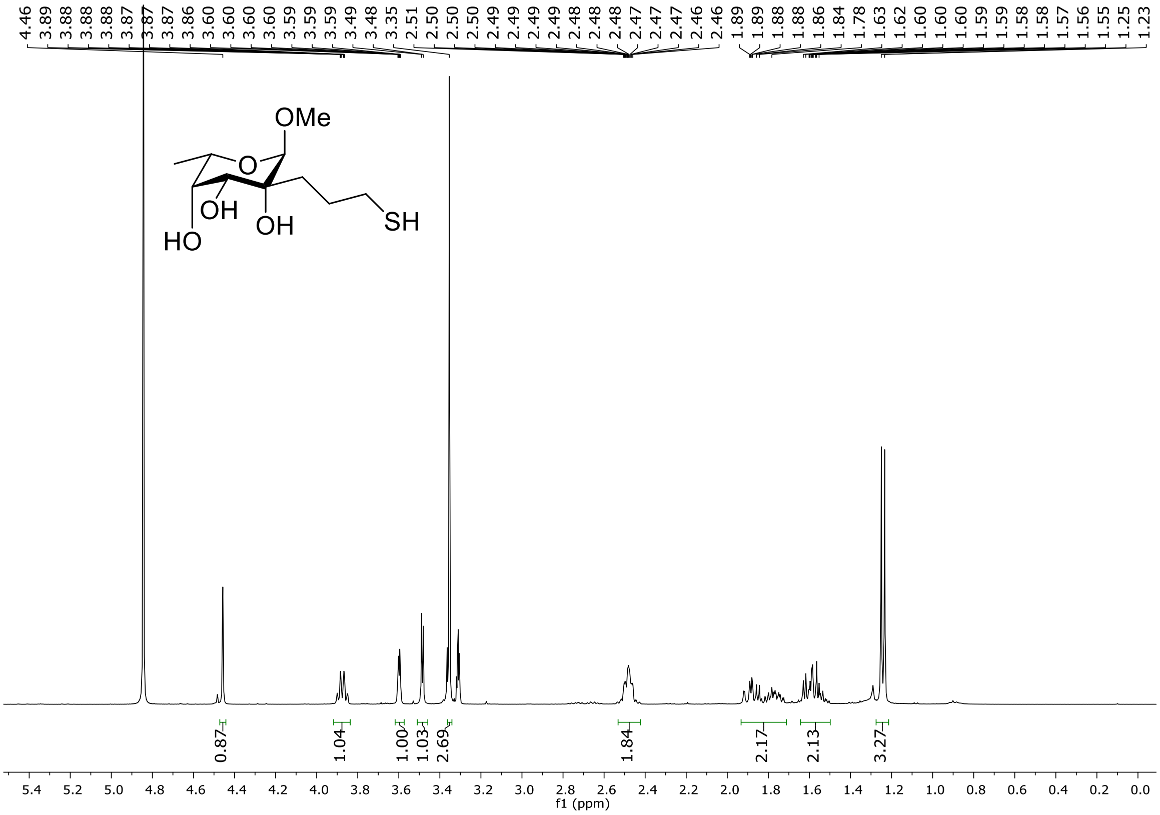

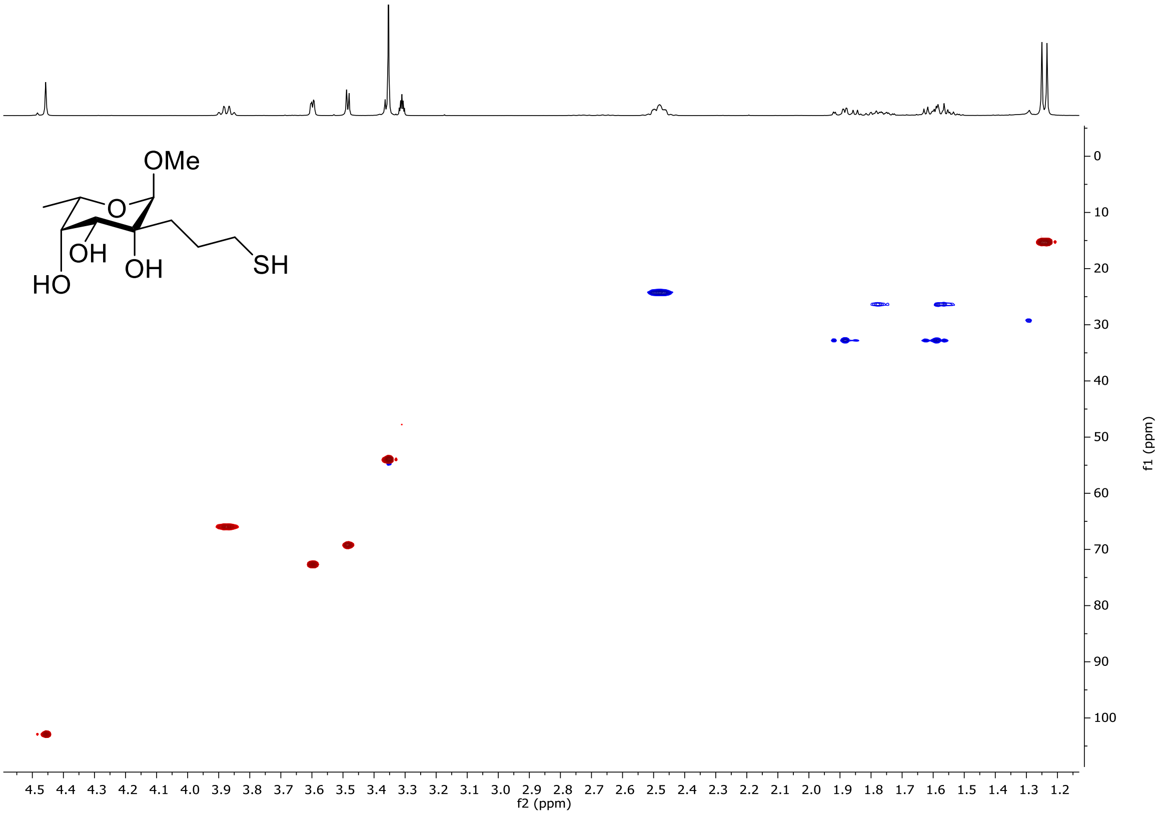

Methyl 4-*O*-acetyl-2,3-*O*-(2’,3’-dimethoxybutane-2’,3’-diyl)-α-L-quinovopyranoside (17)

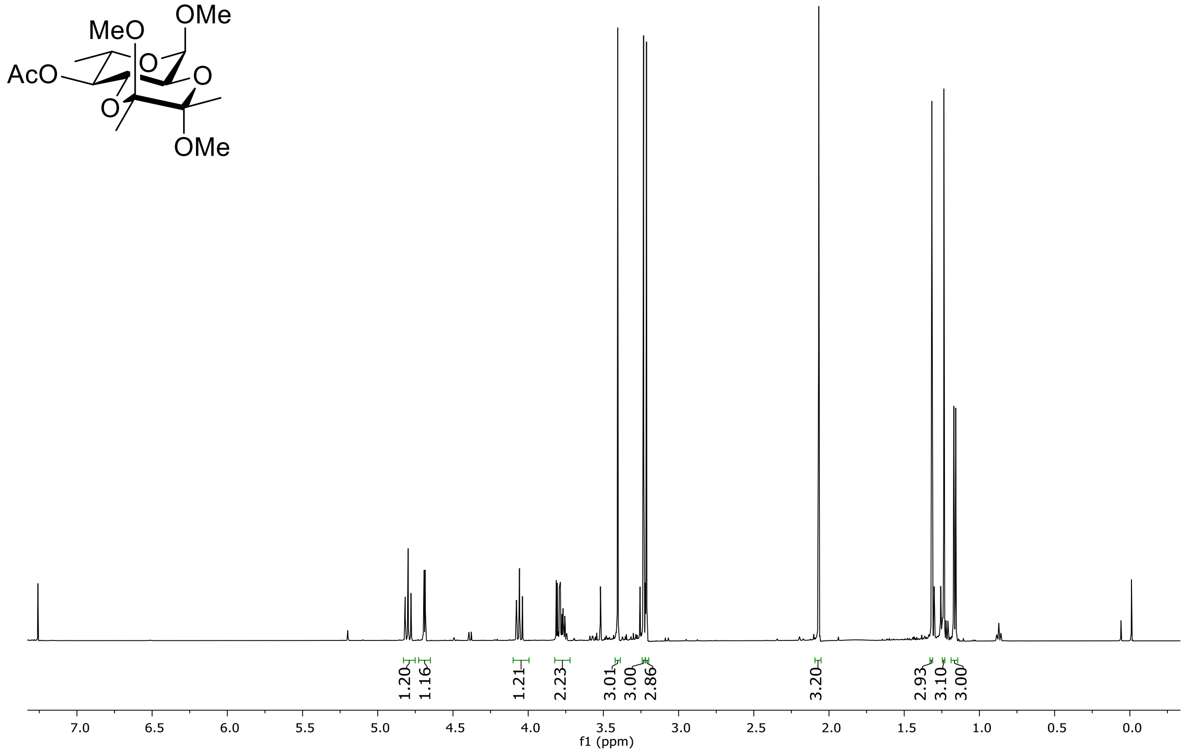

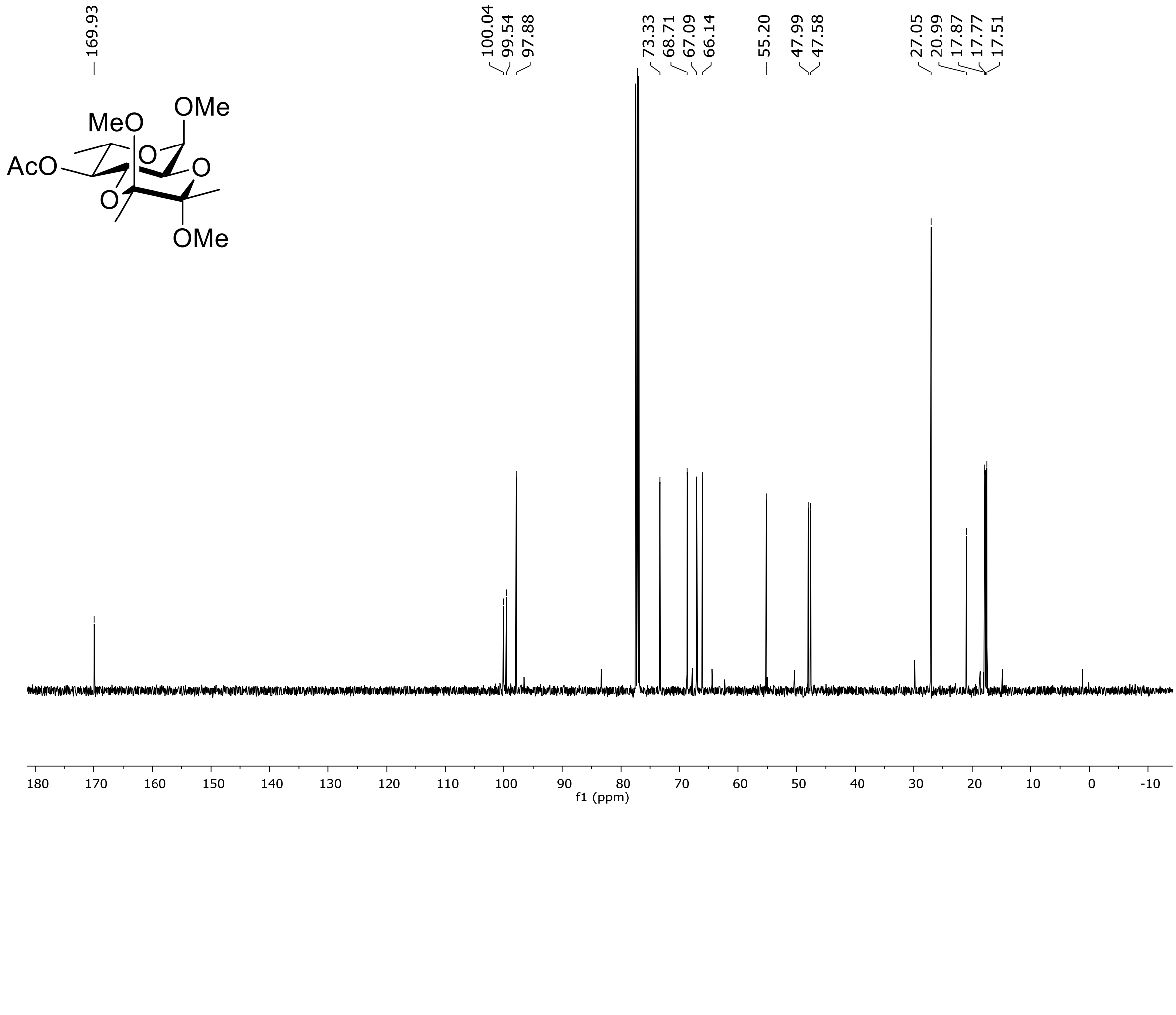

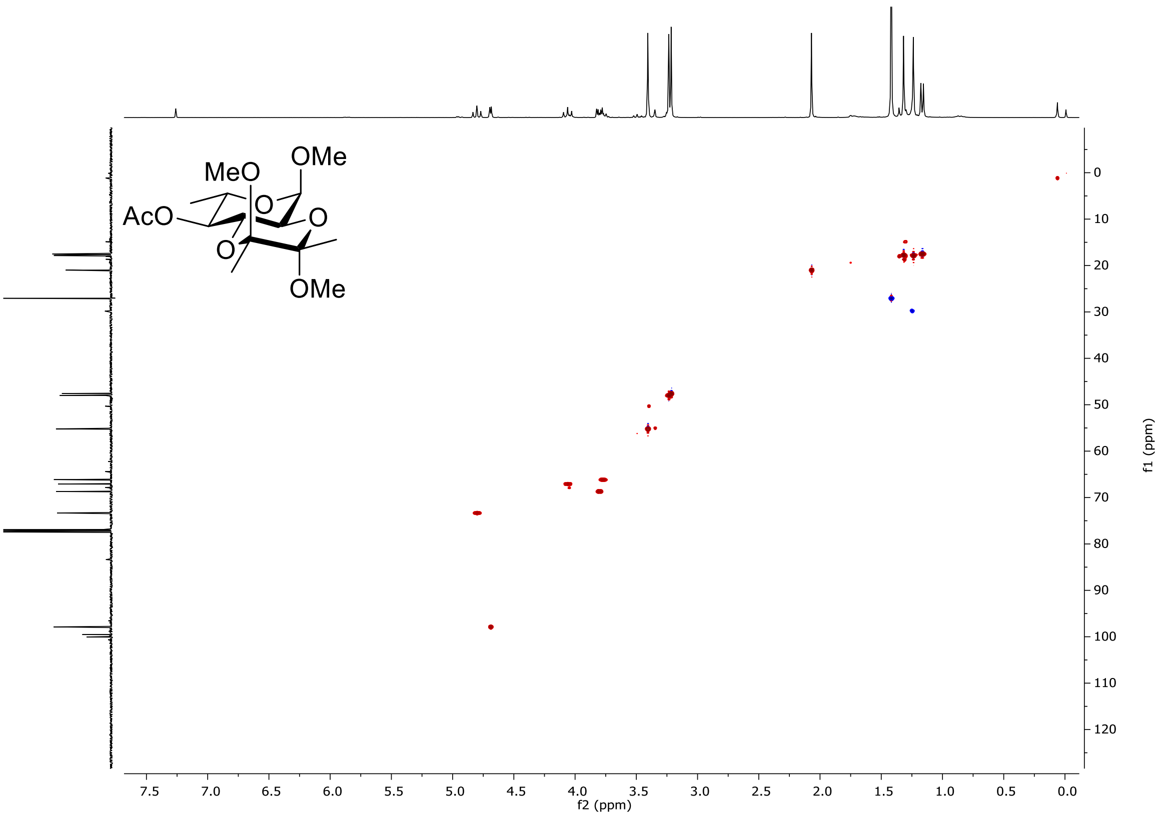
OMe
MeO
AcO
O
O
O
OMe
f2 (ppm)
f1 (ppm)

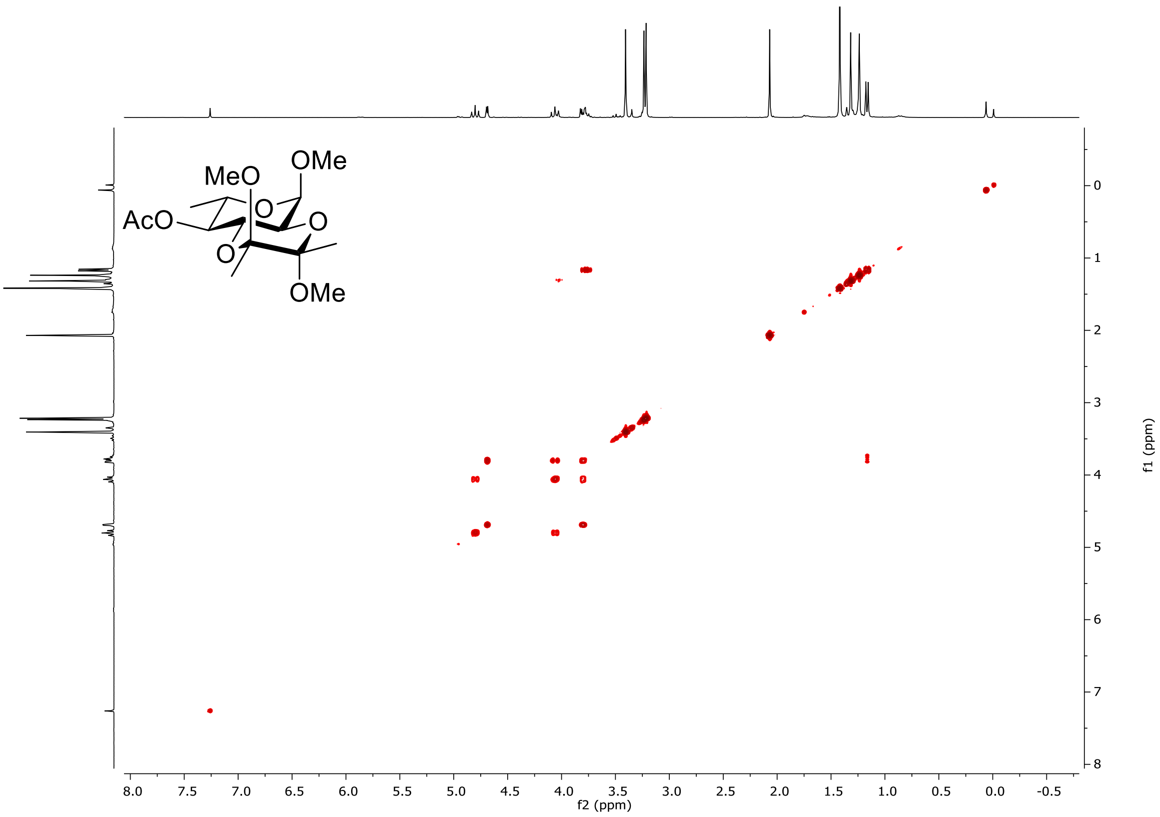
OMe
MeO
AcO
O
O
O
OMe
f2 (ppm)
f1 (ppm)

Methyl 2,3-*O*-(2’,3’-dimethoxybutane-2’,3’-diyl)-α-L-quinovopyranoside (18)

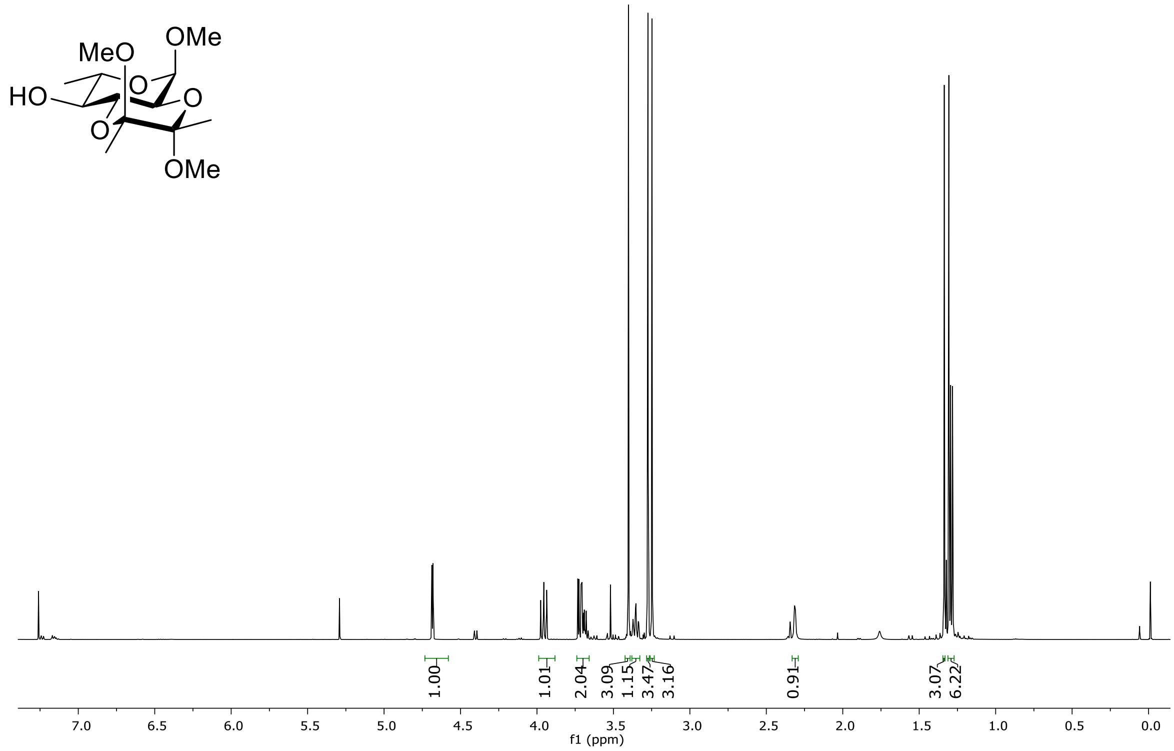

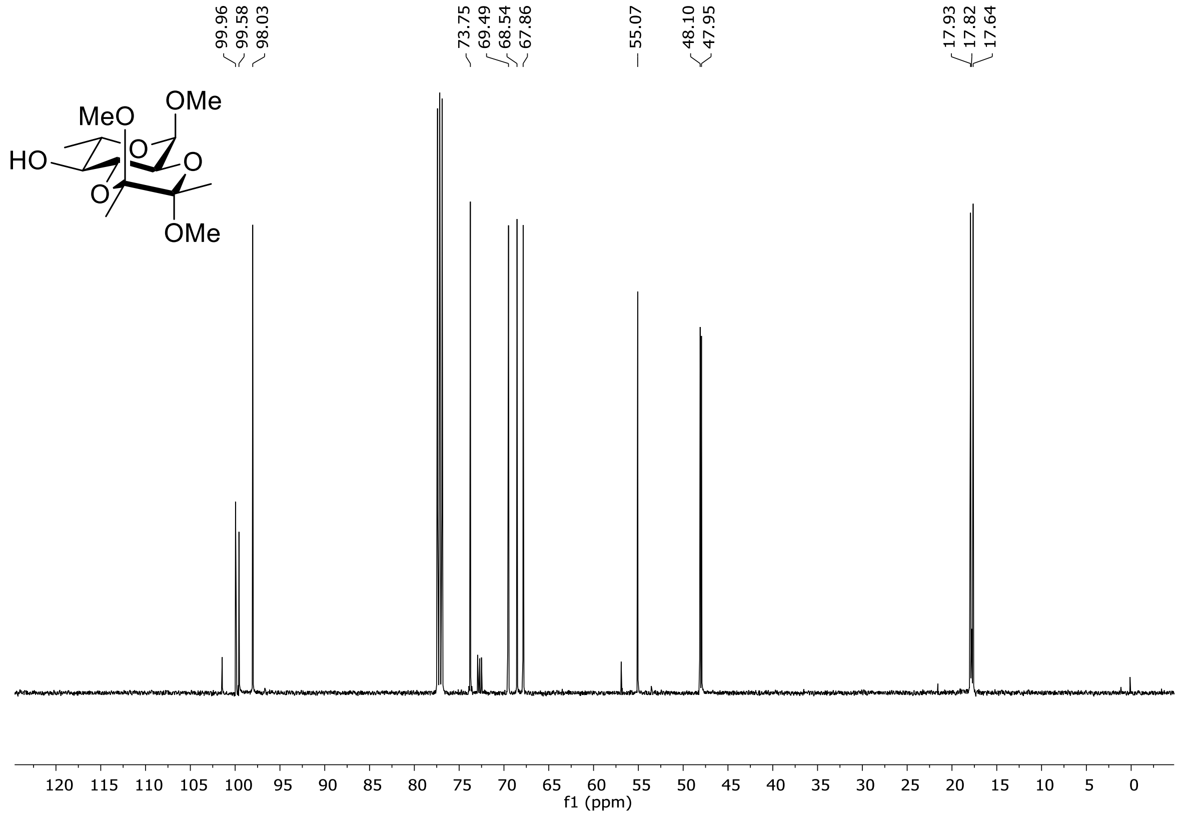

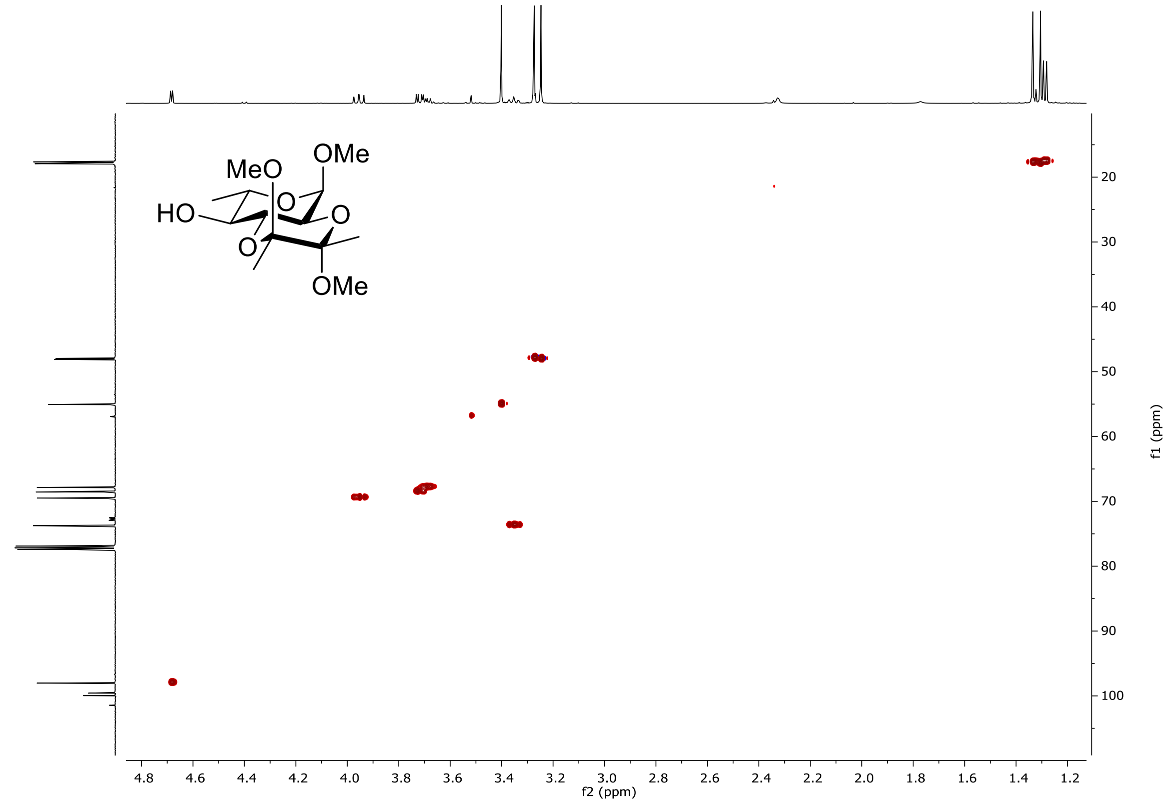

OMe
MeO
HO
O
O
O
OMe
f1 (ppm)
f2 (ppm)

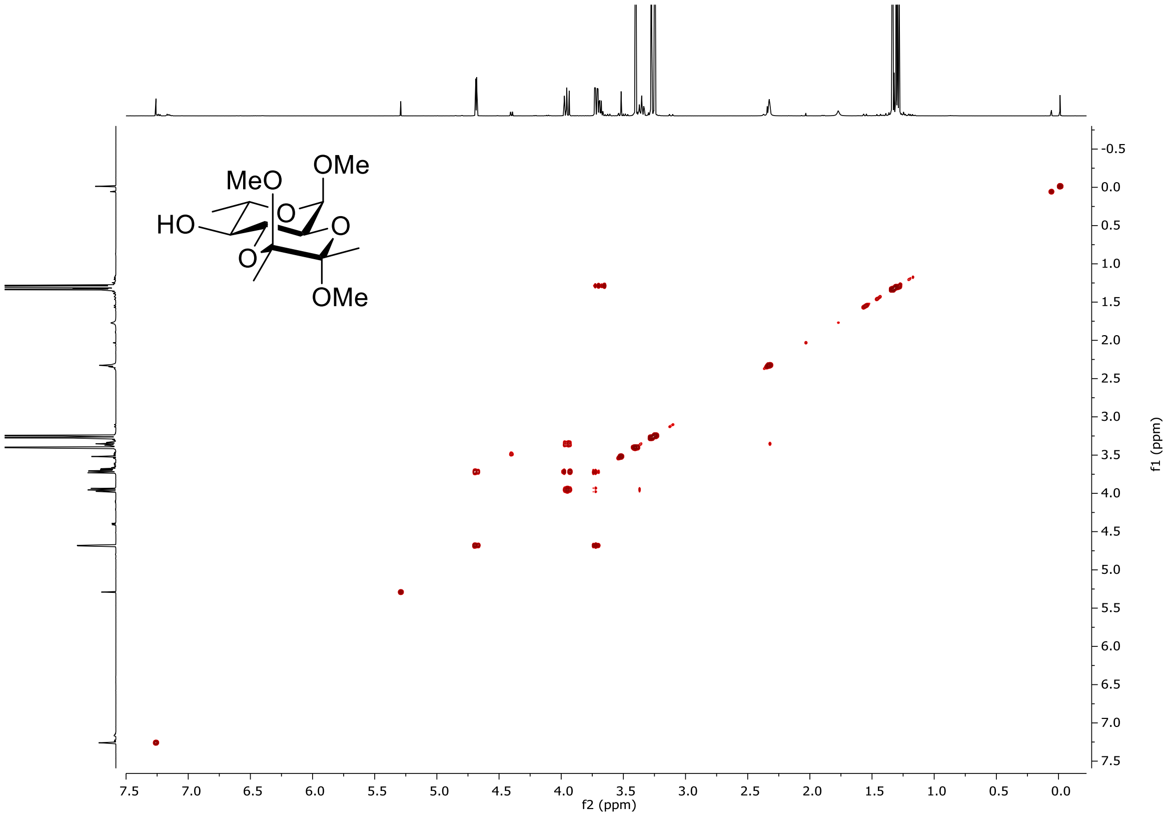

OMe
MeO
HO
O
O
O
OMe
f1 (ppm)
f2 (ppm)

Methyl 4-azido-4-deoxy-2,3-*O*-(2',3'-dimethoxybutane-2',3'-diyl)-α-L-fucopyranoside (19)

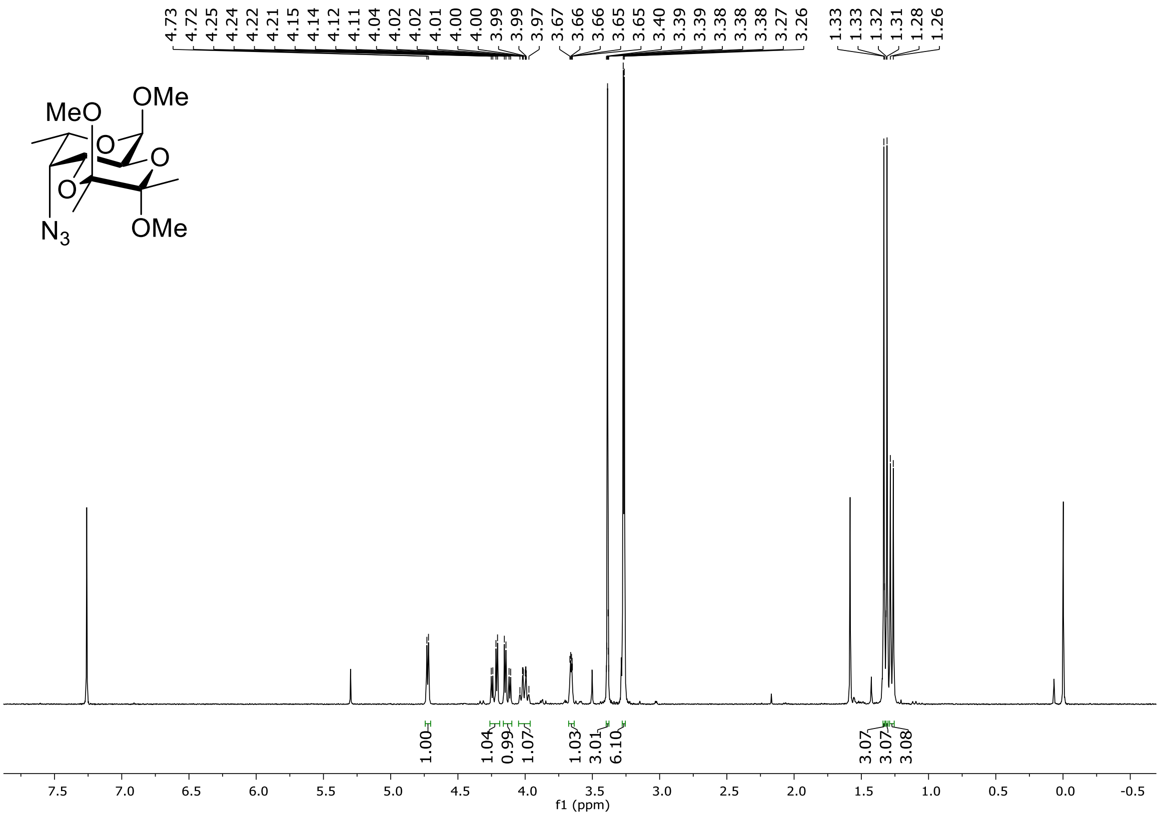

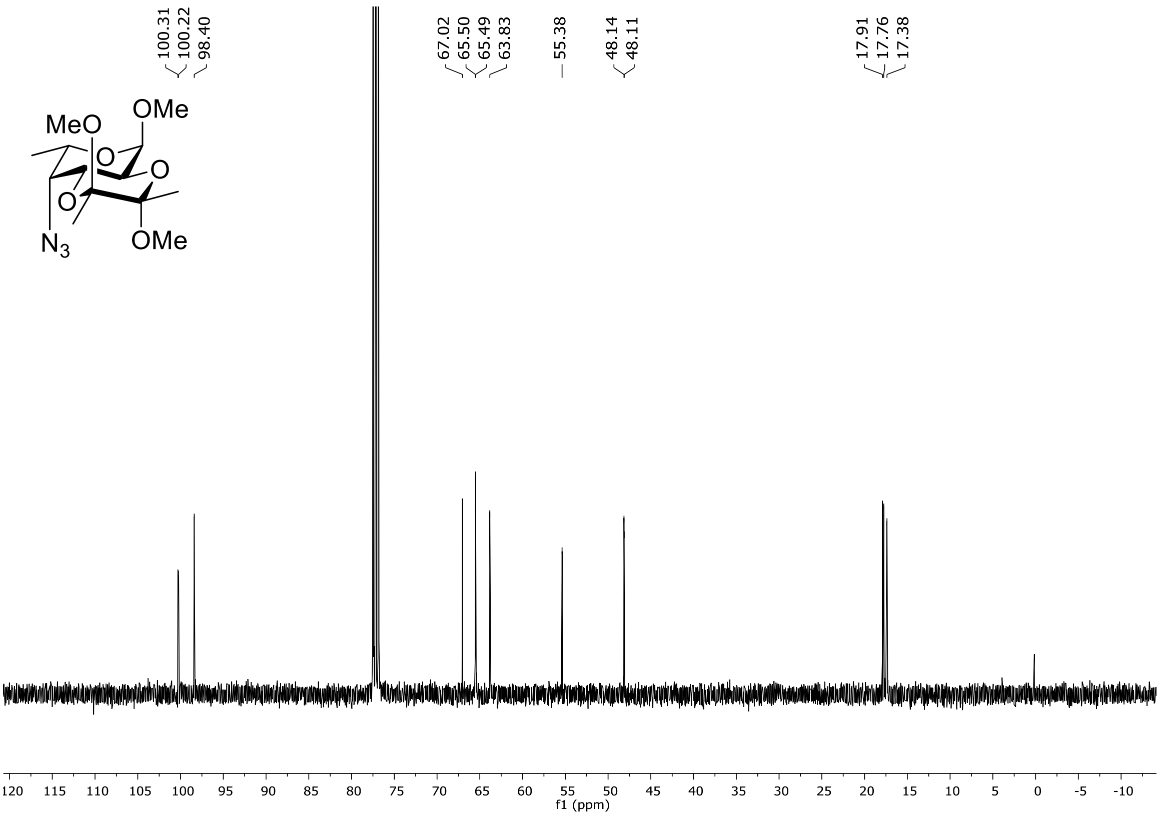

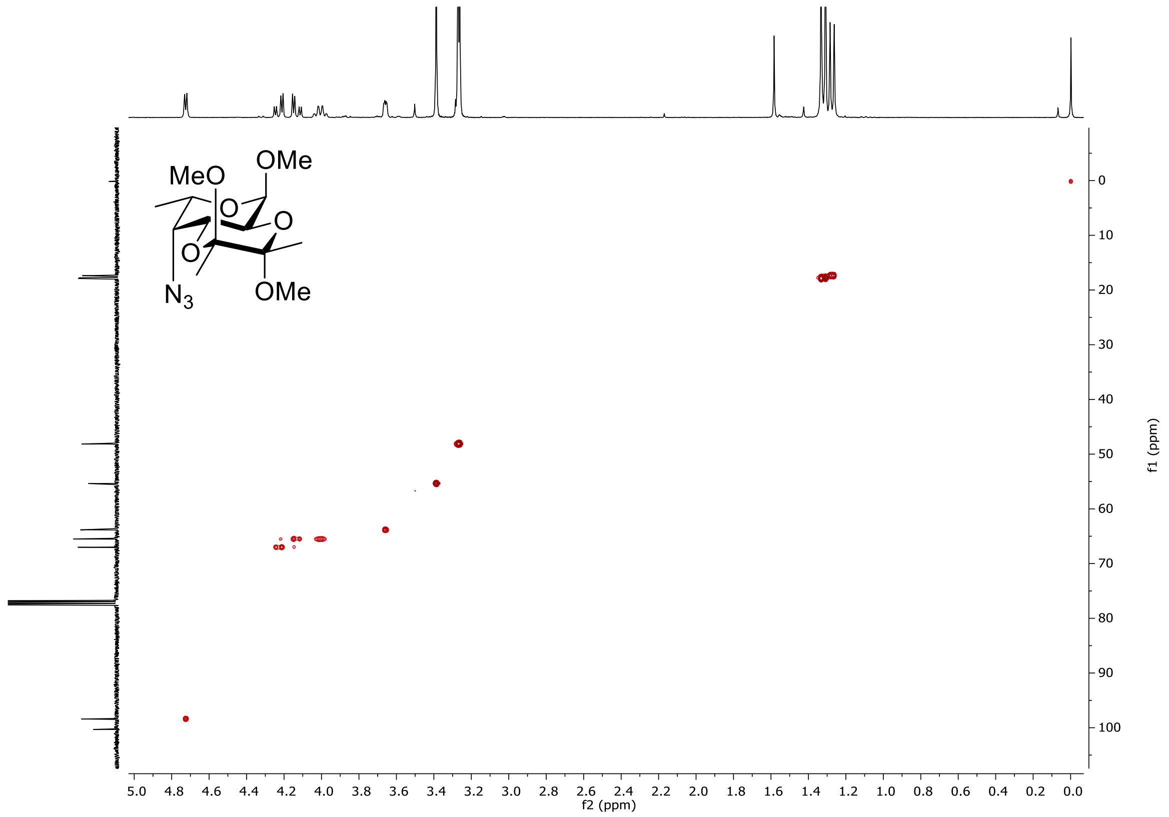
MeO
OMe
O
O
O
N3
OMe
f2 (ppm)
f1 (ppm)

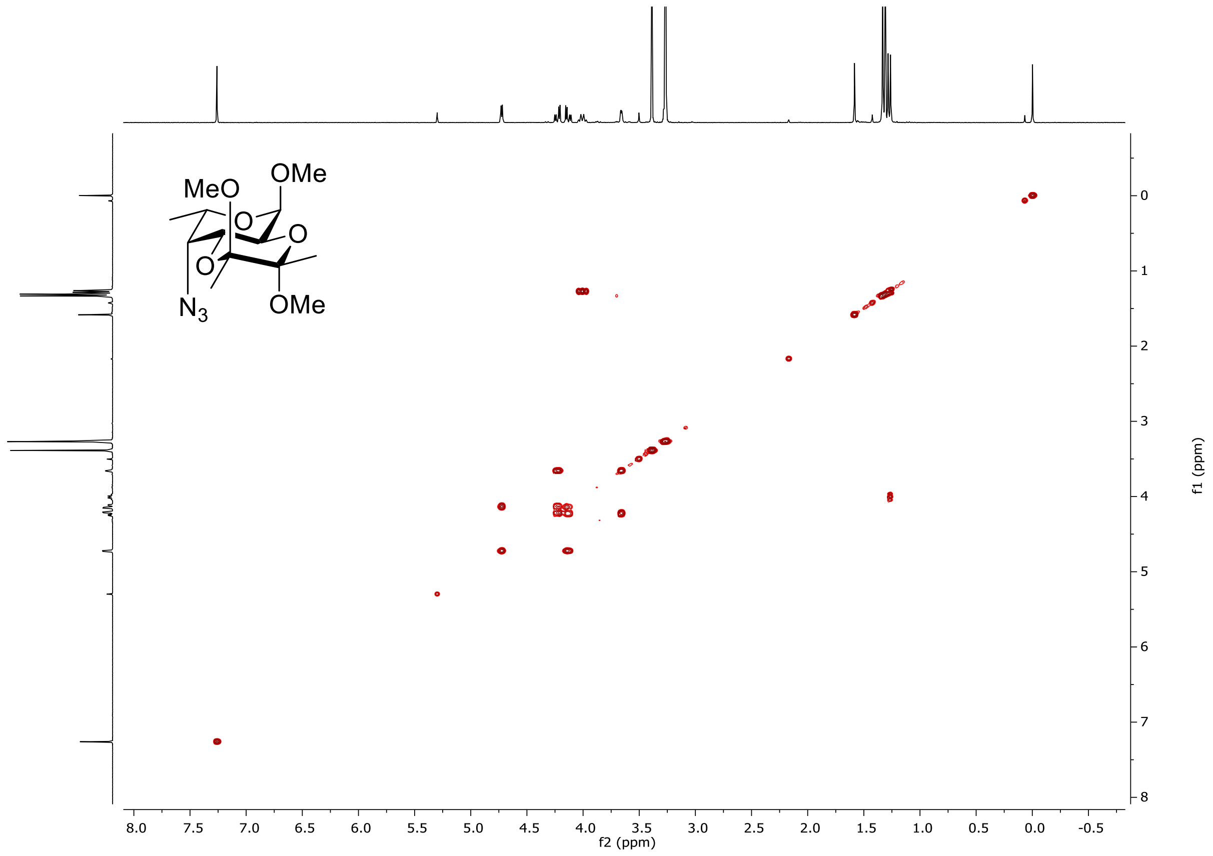
MeO
OMe
O
O
O
N3
OMe
f2 (ppm)
f1 (ppm)

Methyl 4-amino-4-deoxy-2,3-*O*-(2’,3’-dimethoxybutane-2’,3’-diyl)-α-L-fucopyranoside (20)

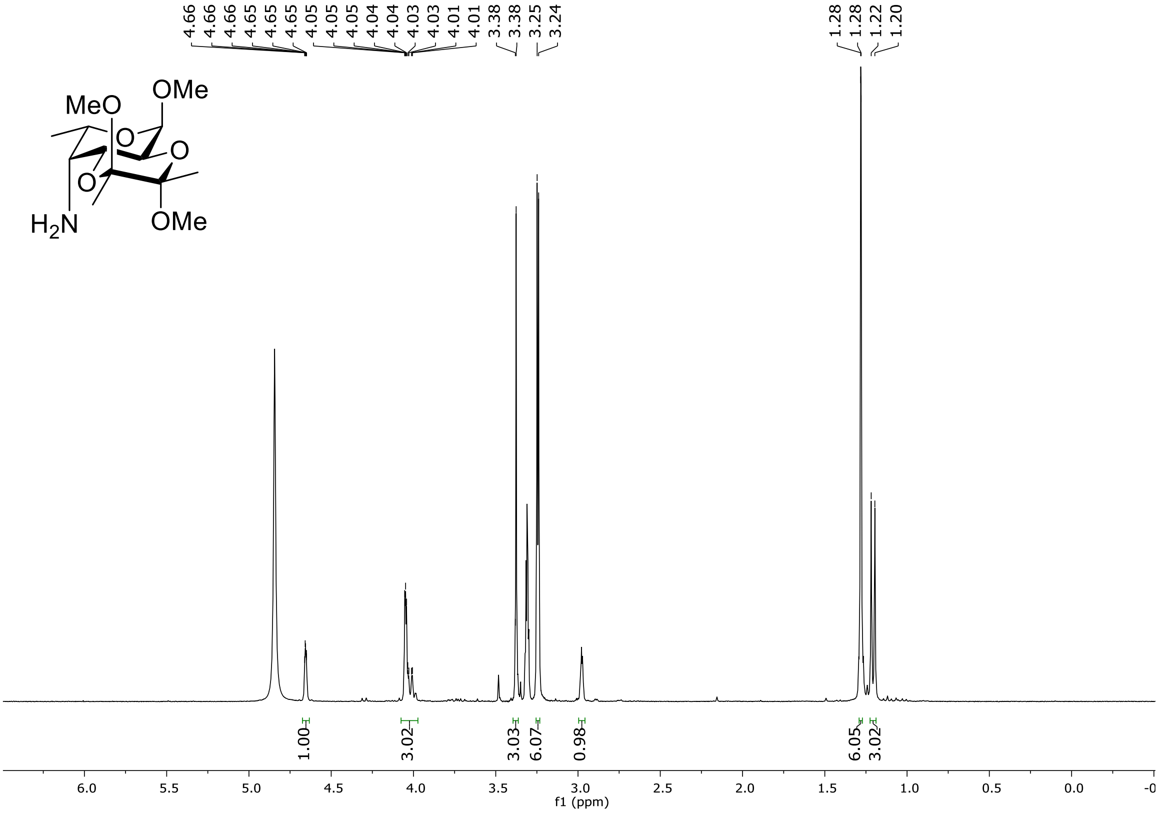

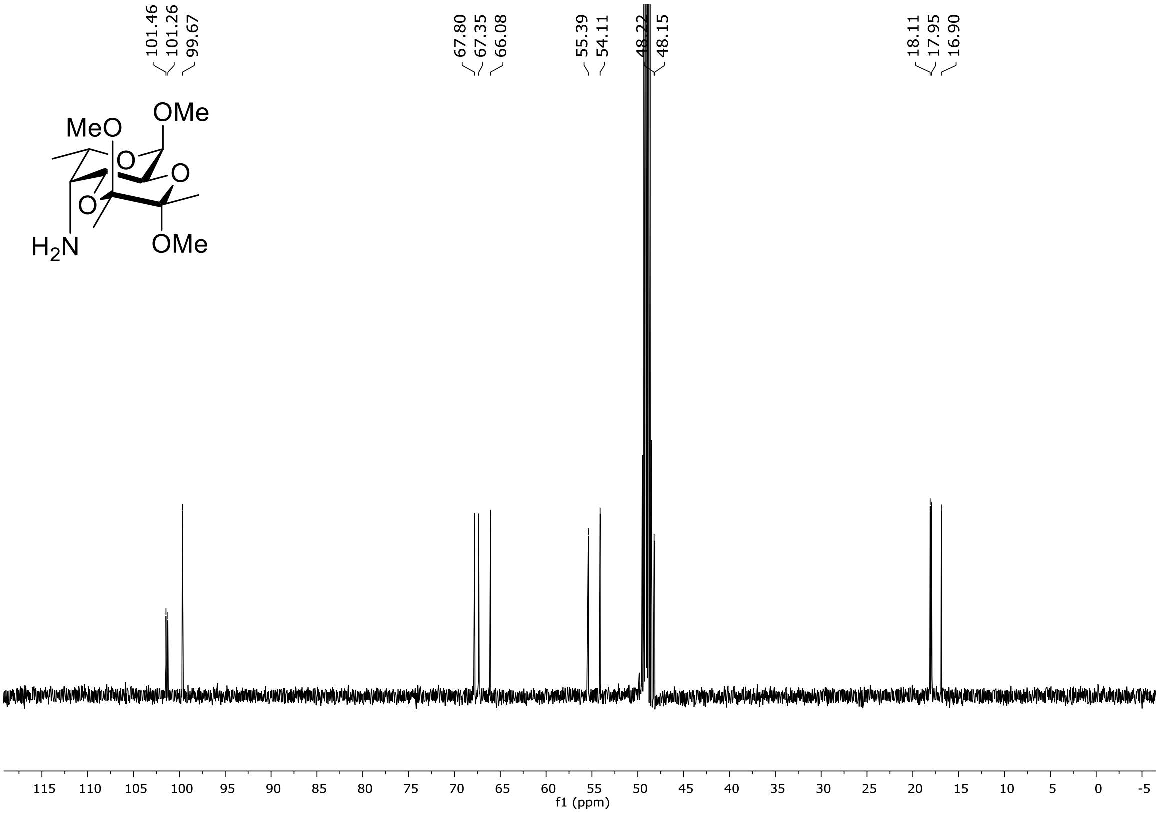

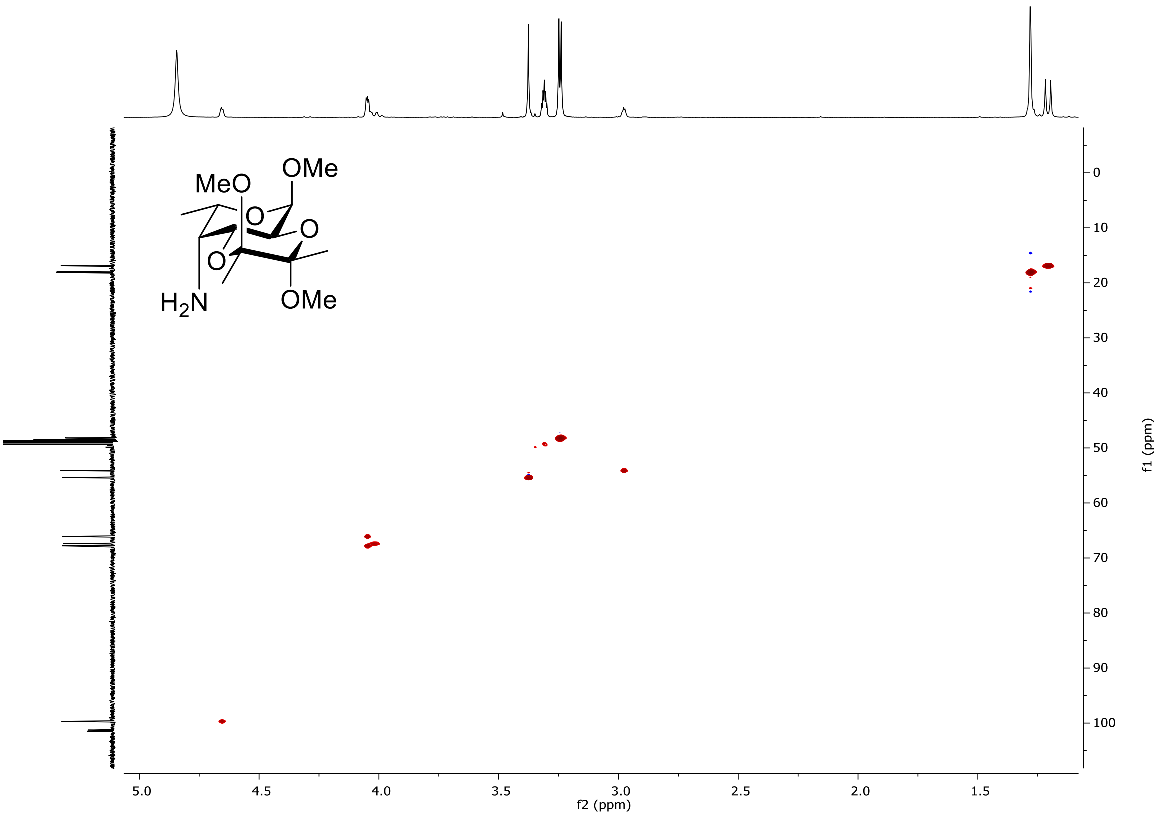
OMe
MeO
O
O
O
H2N
OMe
f1 (ppm)
f2 (ppm)

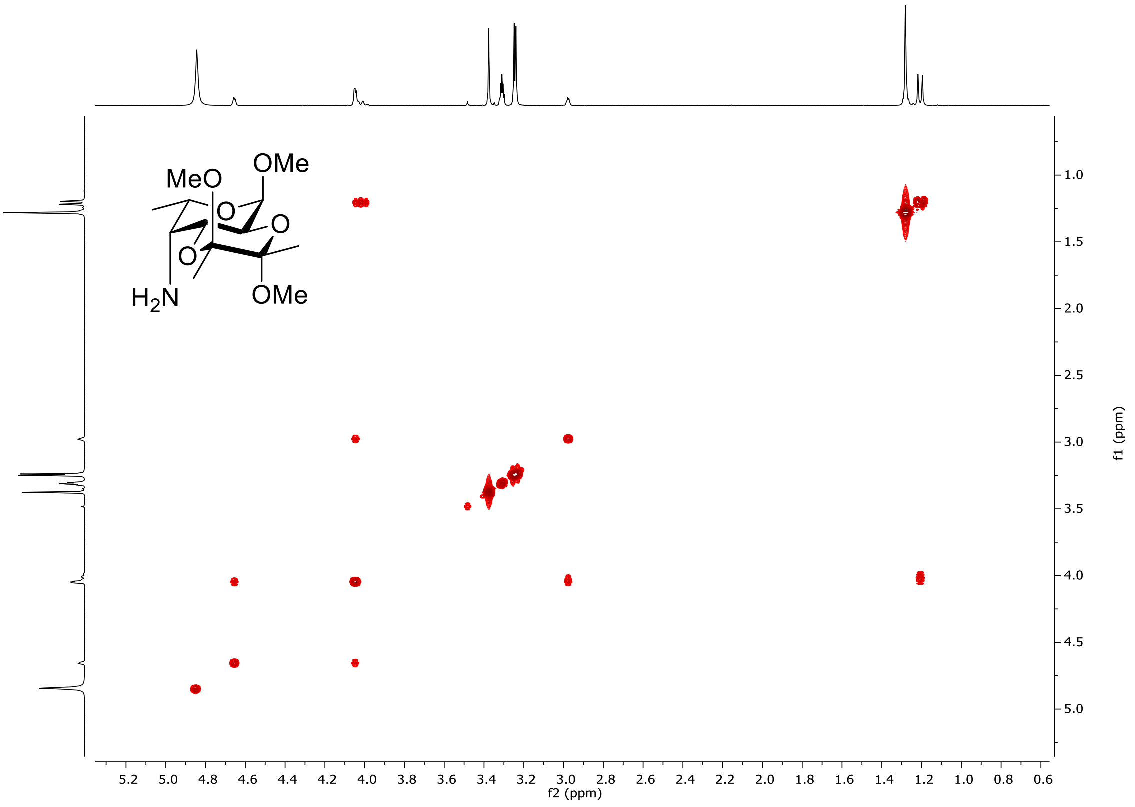
OMe
MeO
O
O
O
H2N
OMe
f1 (ppm)
f2 (ppm)

2-Bromoethyl thioacetate (21) [Ref. 31]

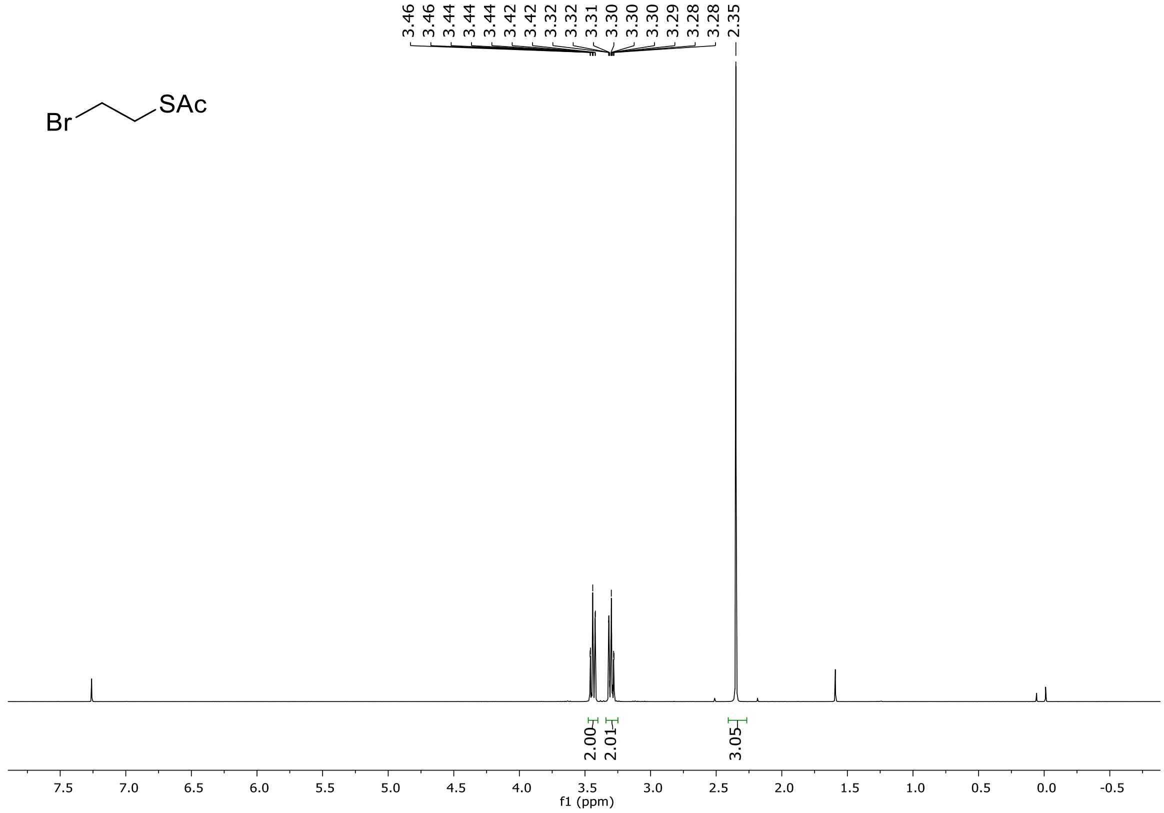

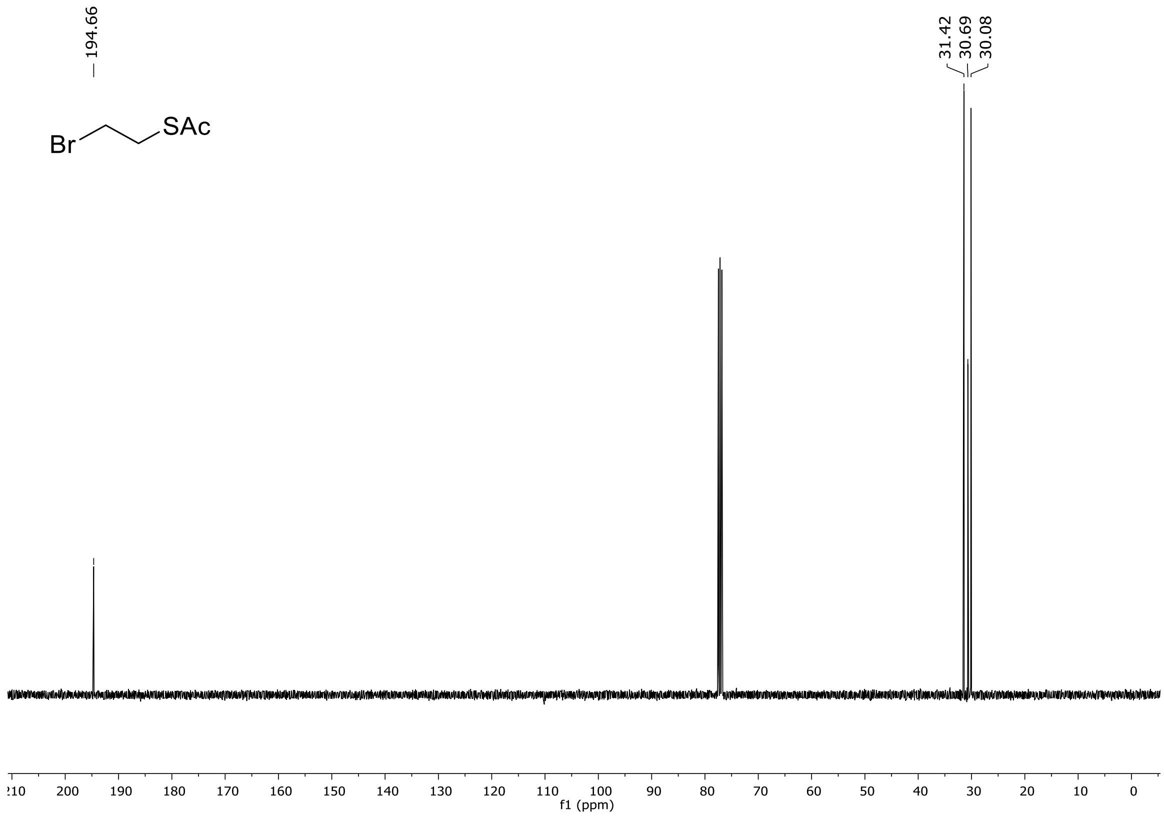

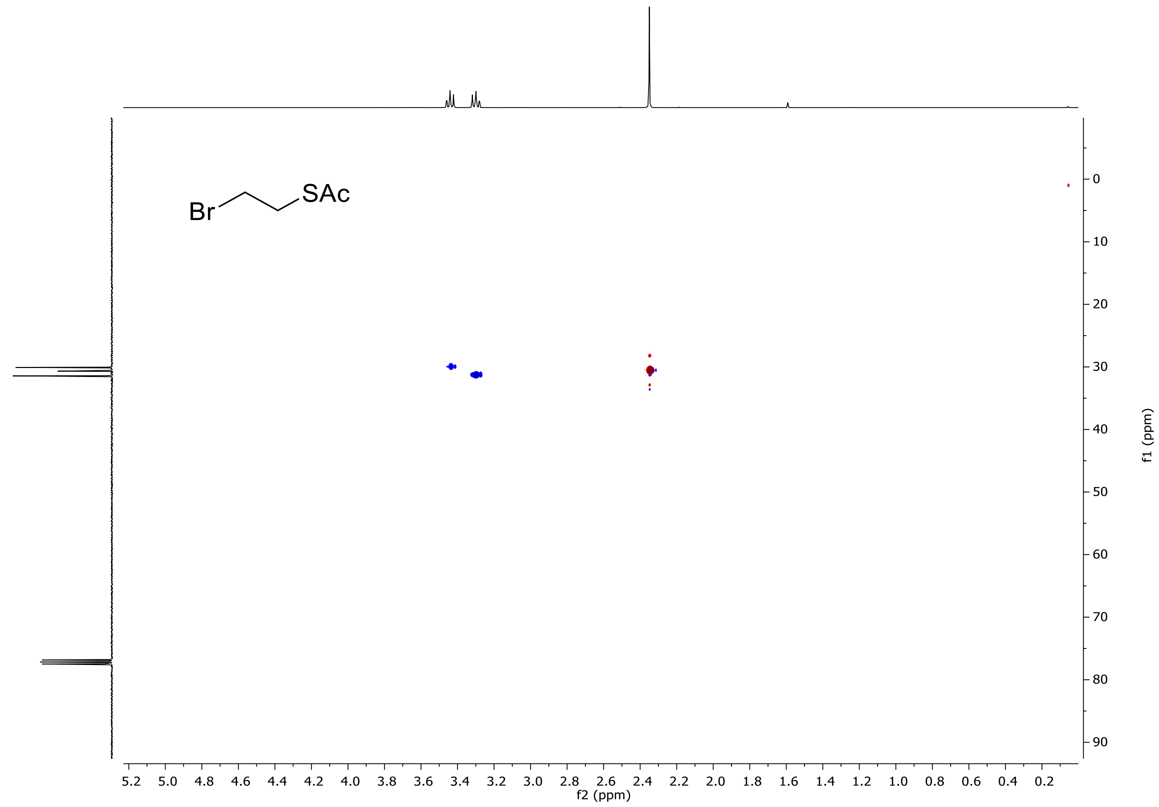
Br
SAc
f1 (ppm)
f2 (ppm)

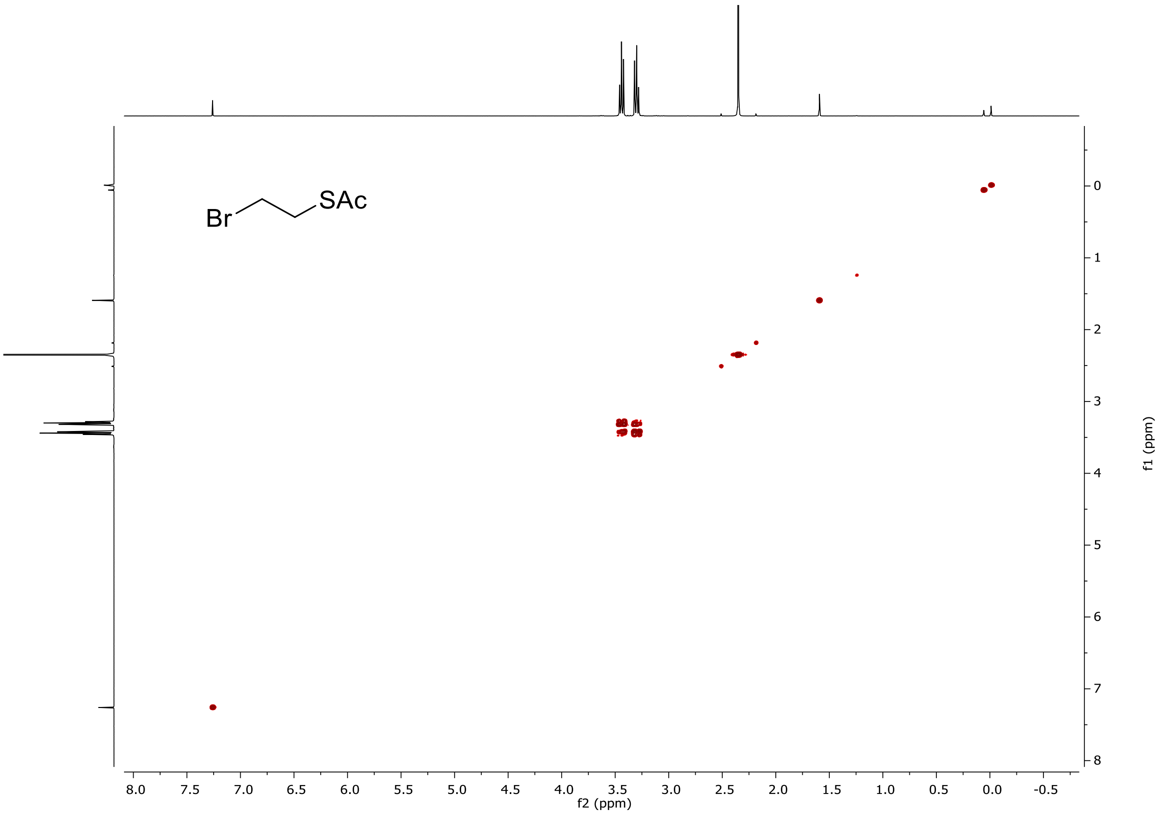
Br
SAc
f1 (ppm)
f2 (ppm)

Methyl 4-[(2-acetylthioethyl)amino]-4-deoxy-2,3-*O*-(2',3'-dimethoxybutane-2',3'-diyl)-α-L-fucopyranoside (22)

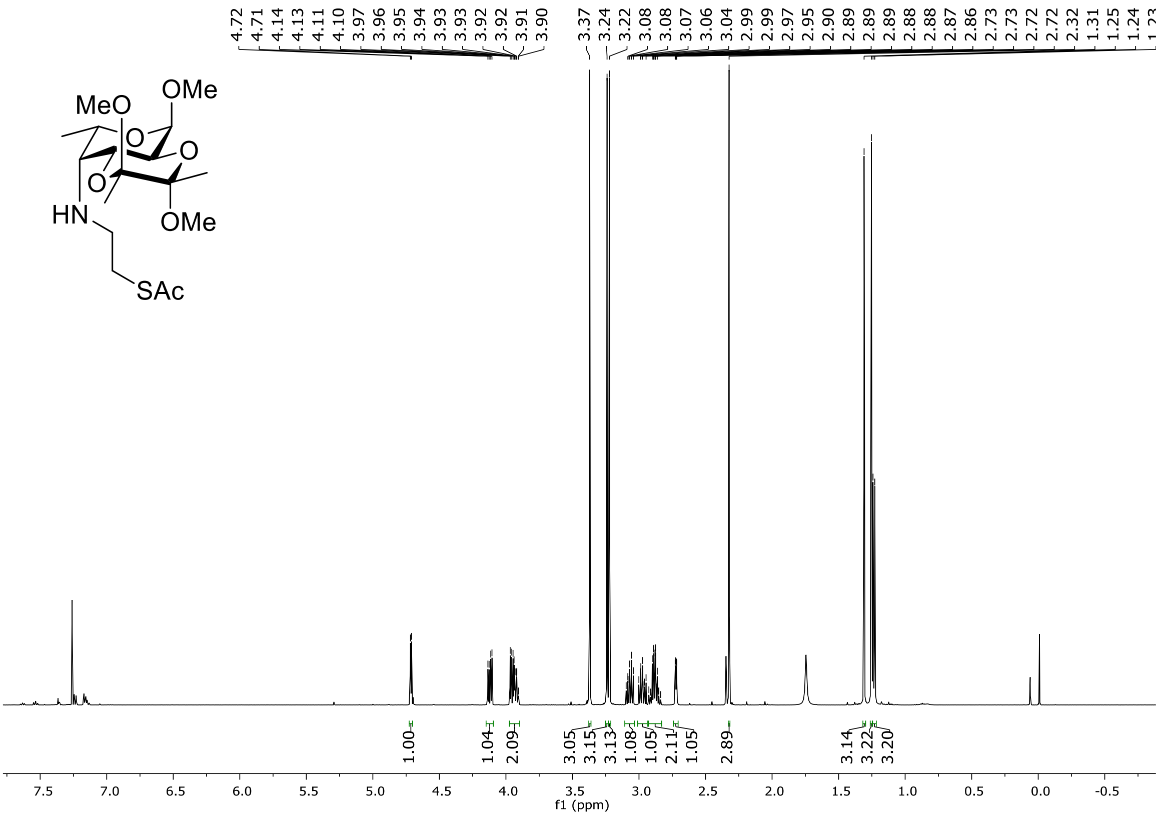

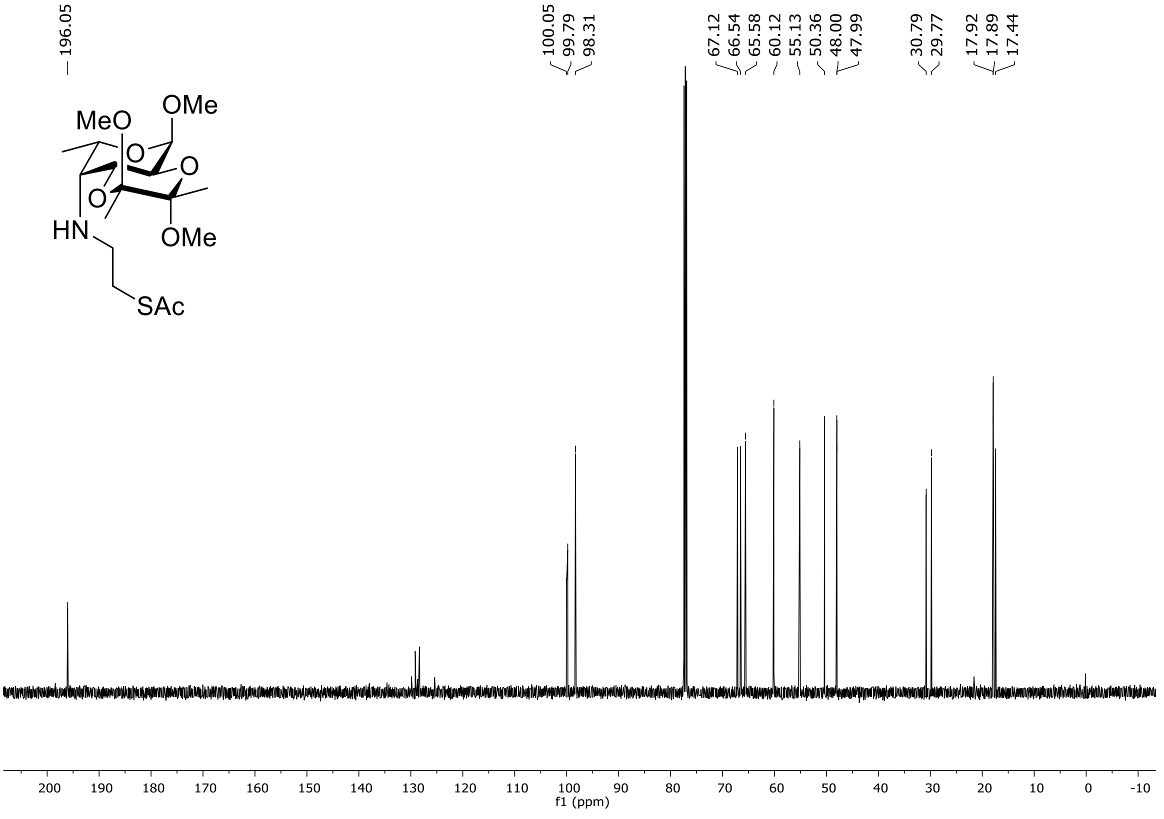

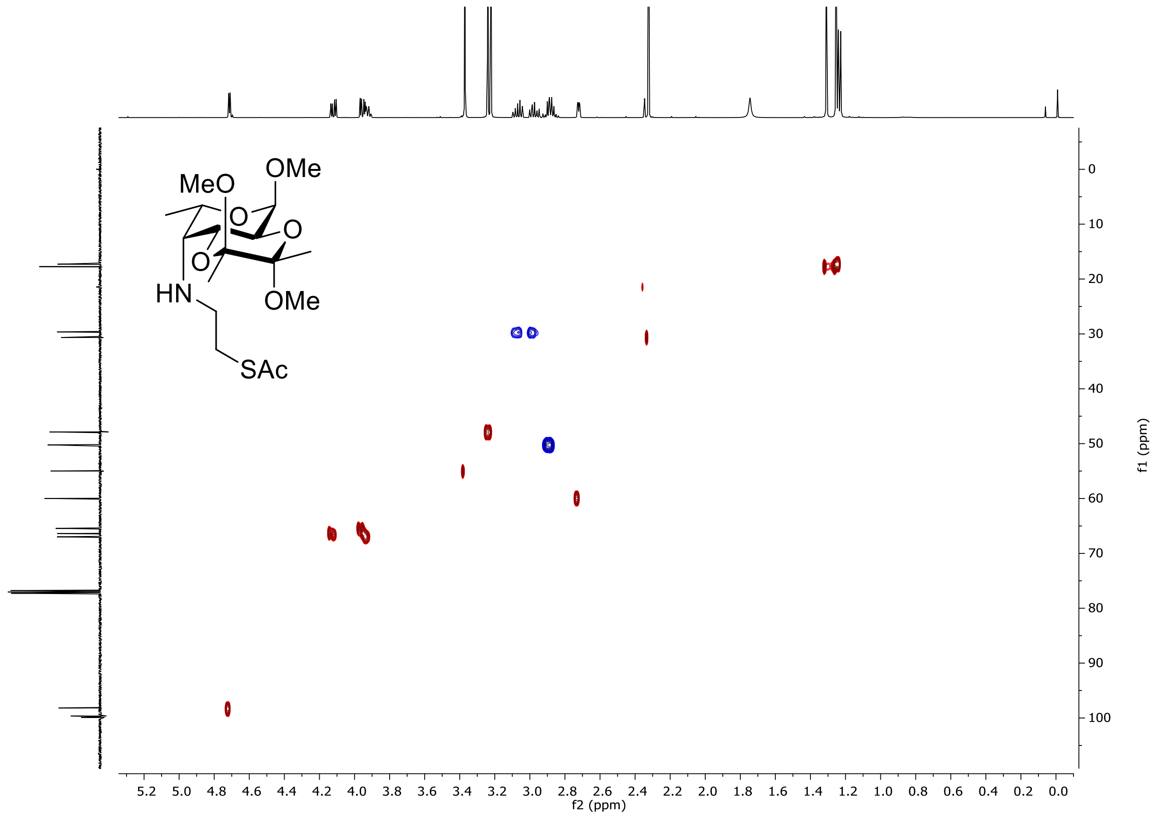
OMe
MeO
O
O
O
HN
OMe
SAc
f1 (ppm)
f2 (ppm)

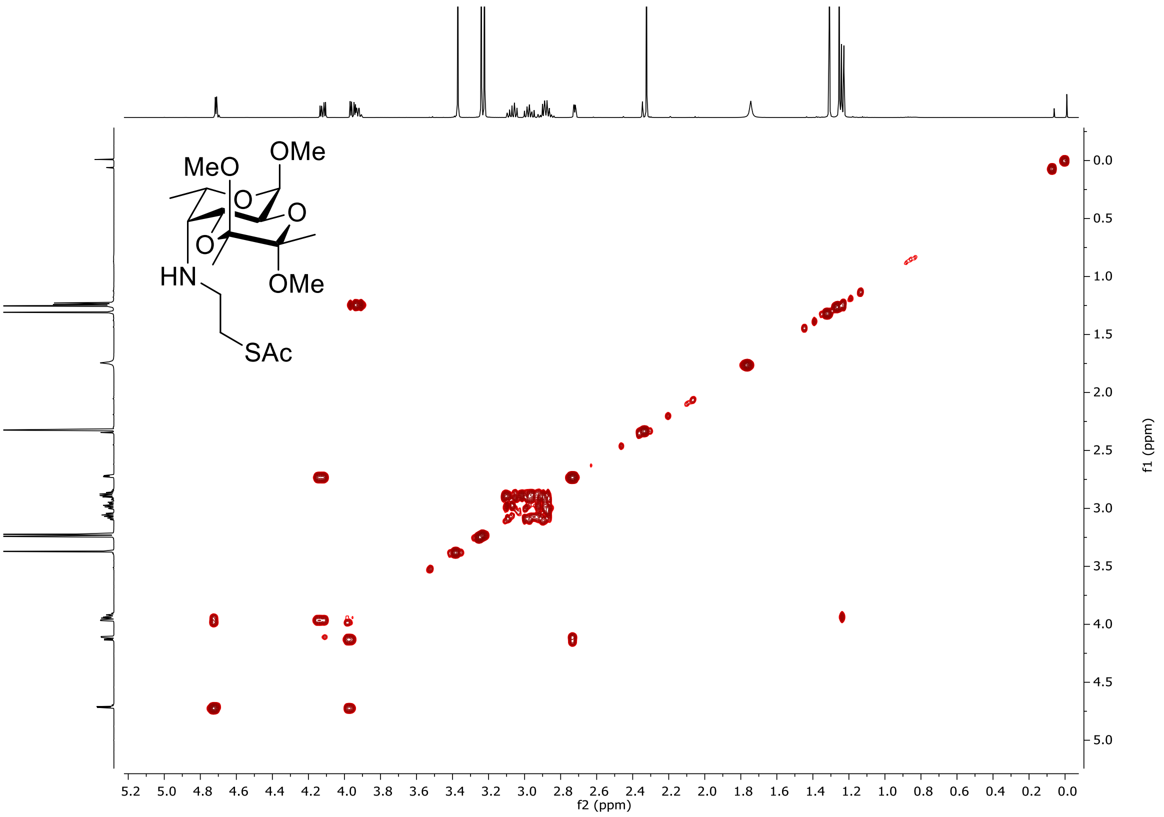
OMe
MeO
O
O
O
HN
OMe
SAc
f1 (ppm)
f2 (ppm)

Methyl 4-[(2-acetylthioethyl)amino]-4-deoxy-α-L-fucopyranoside (23)

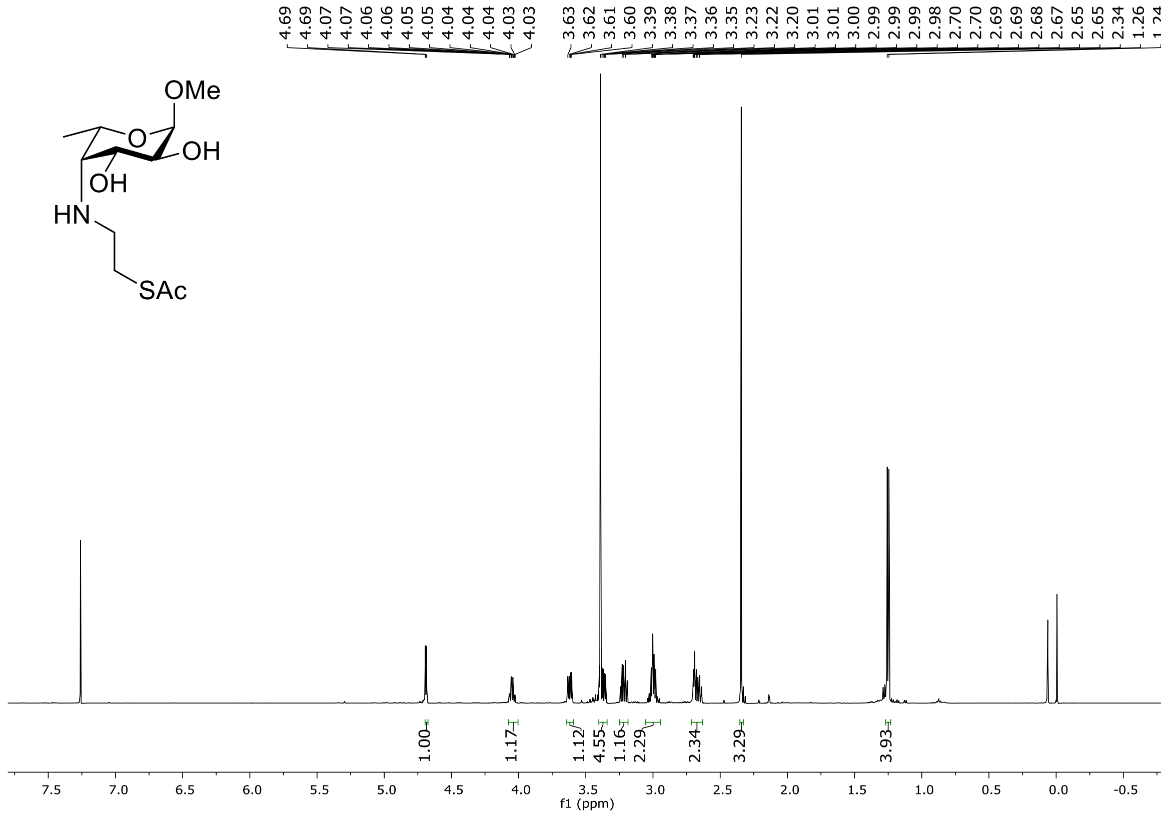

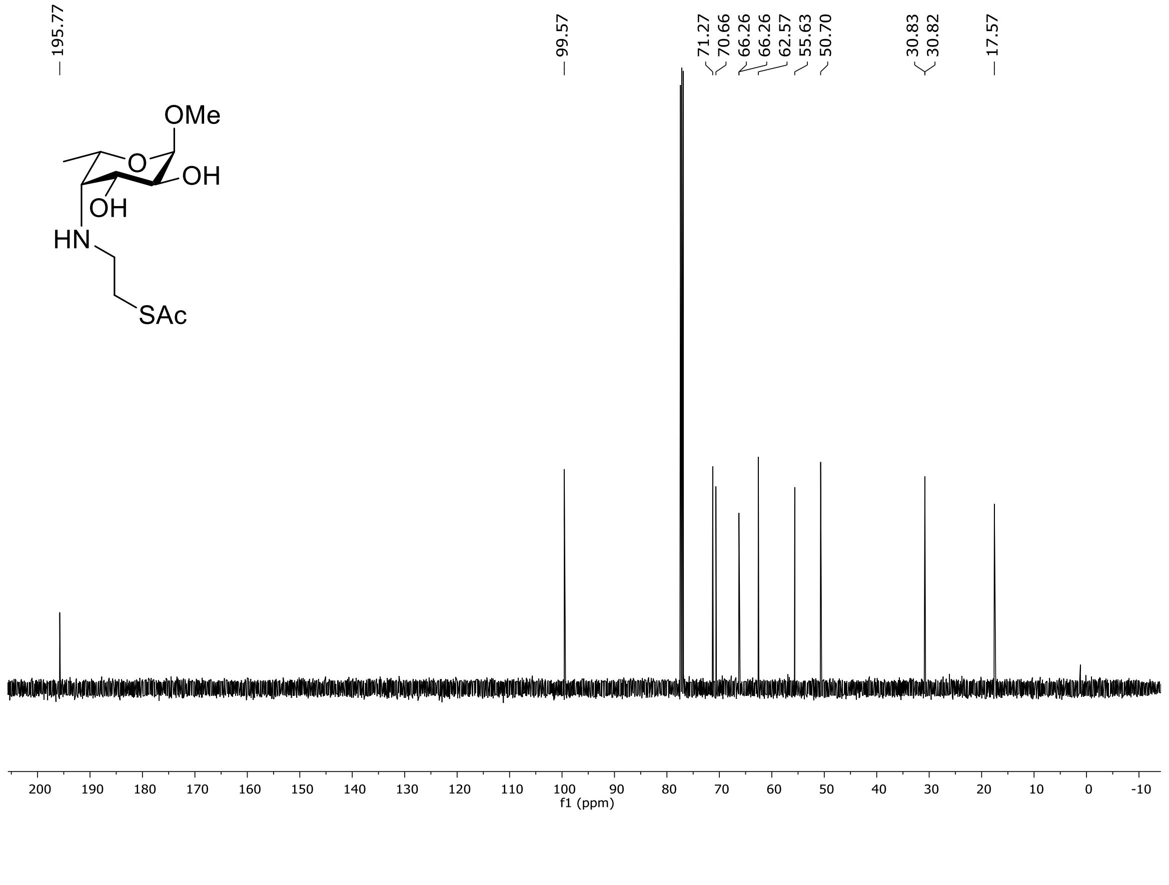

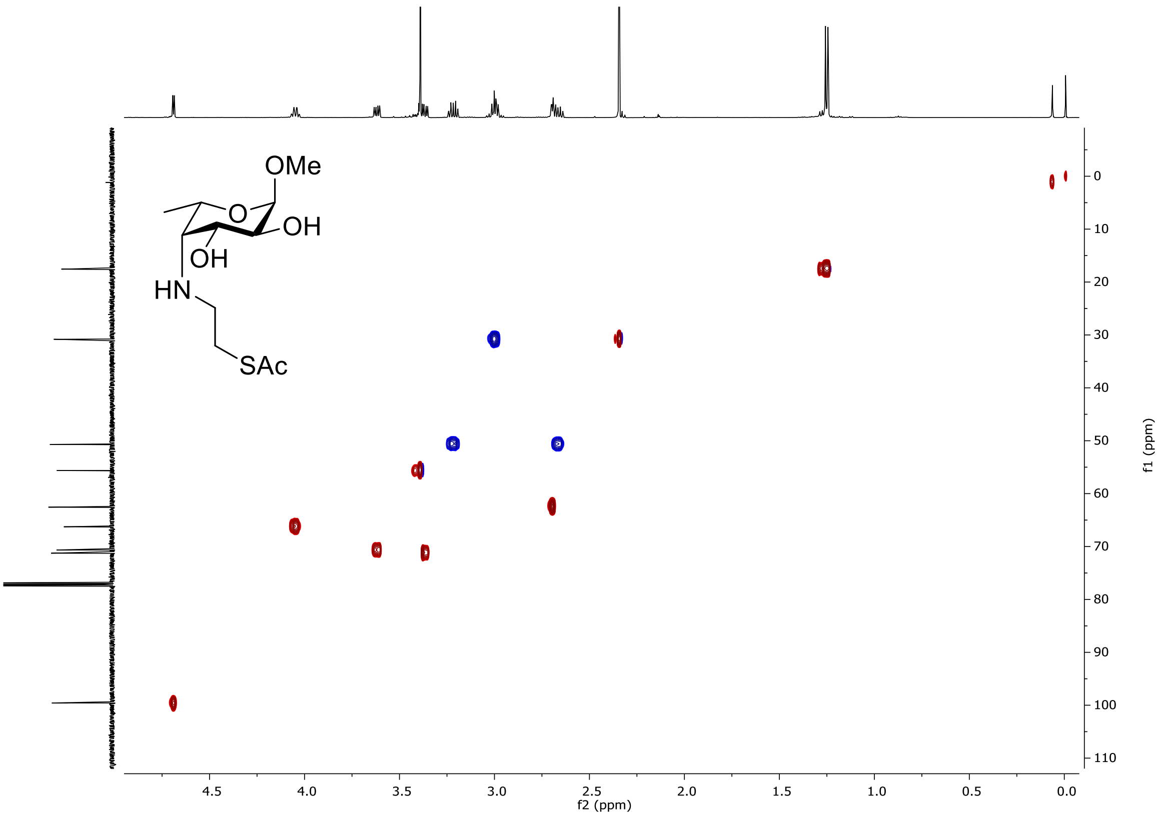
OMe
O
OH
OH
HN
SAc
f2 (ppm)
f1 (ppm)

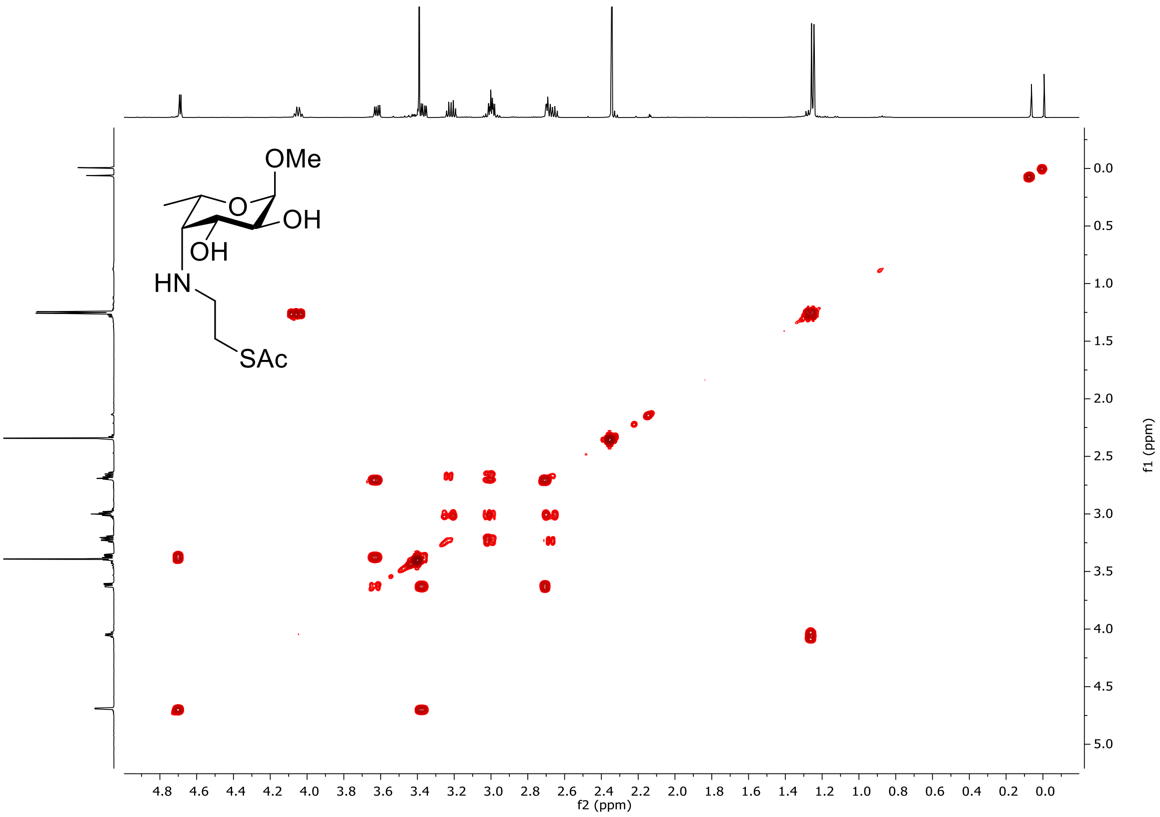
OMe
O
OH
OH
HN
SAc
f2 (ppm)
f1 (ppm)

2-Bromoethyl thiobenzoate (24) [Ref. 35]

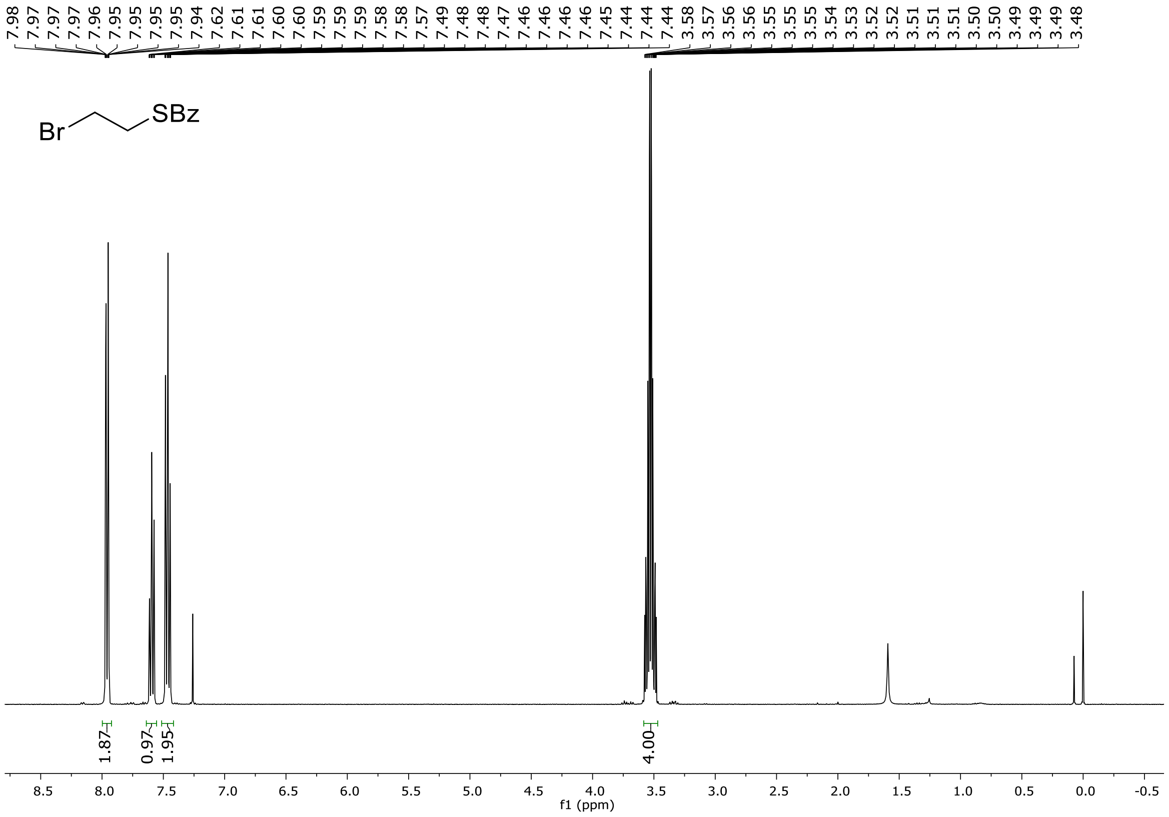

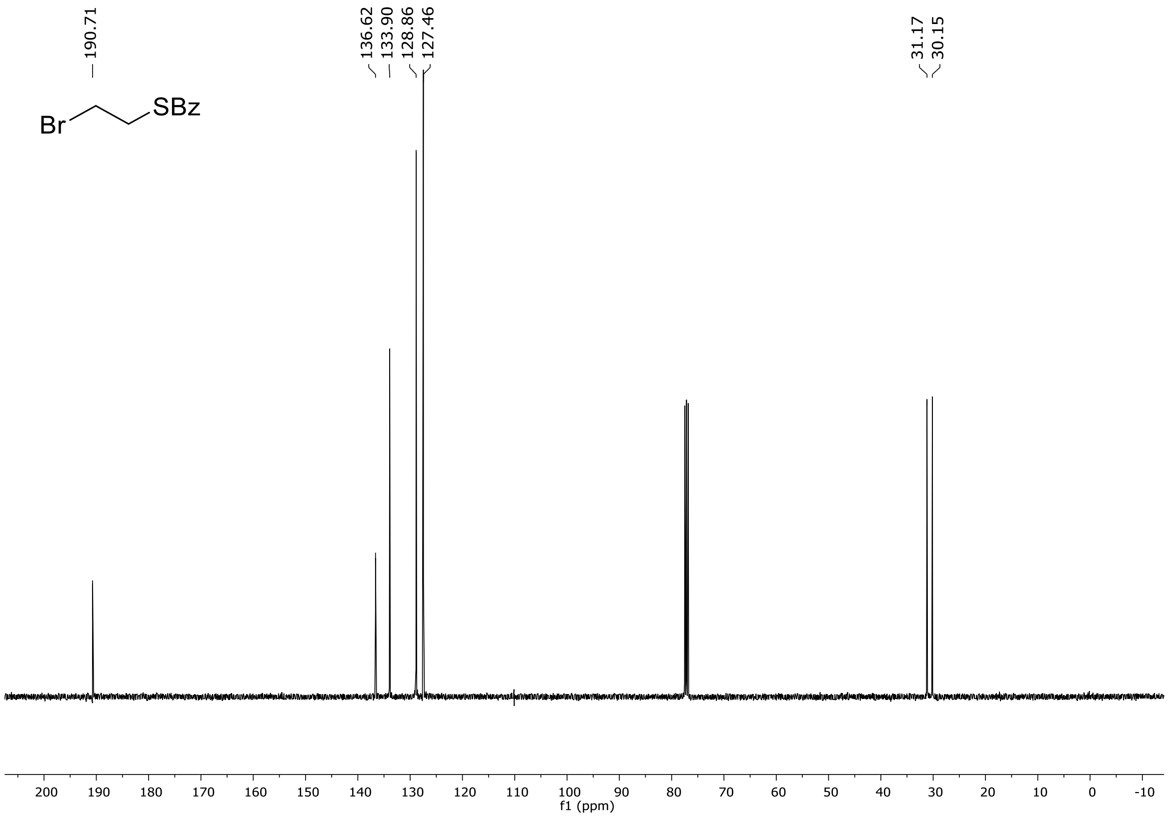

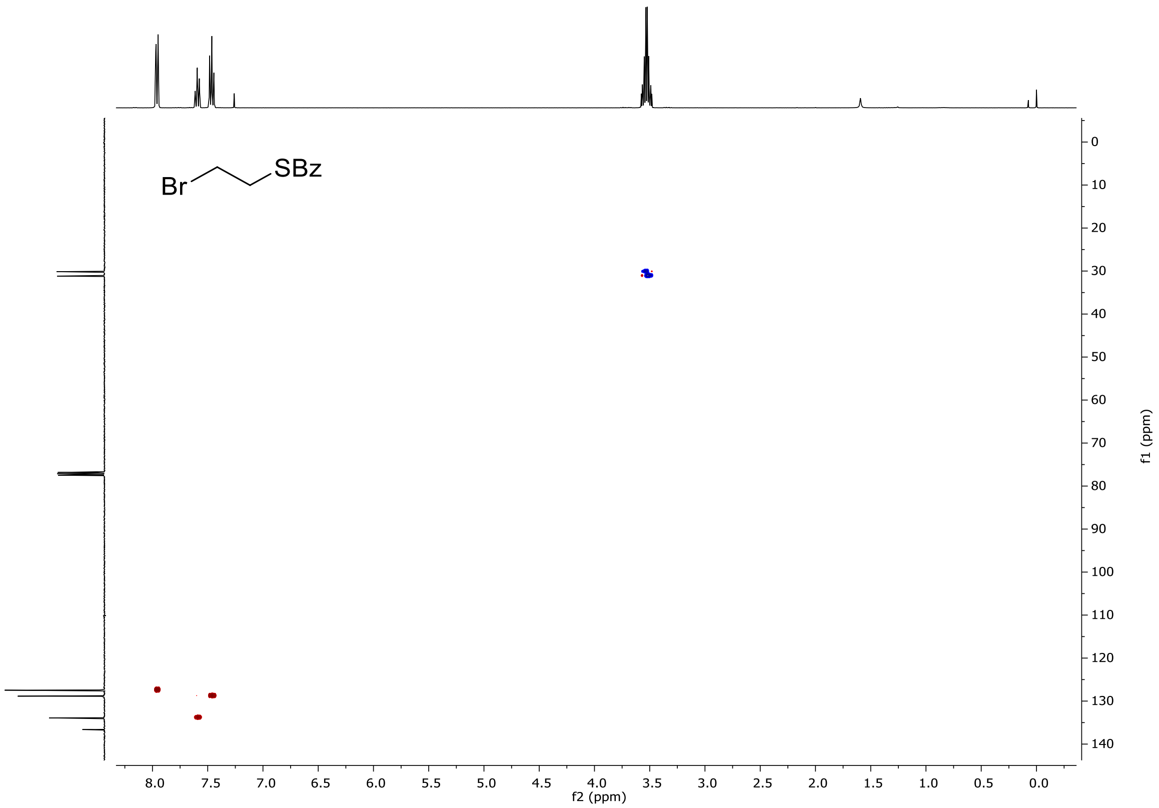
Br
SBz
f2 (ppm)
f1 (ppm)

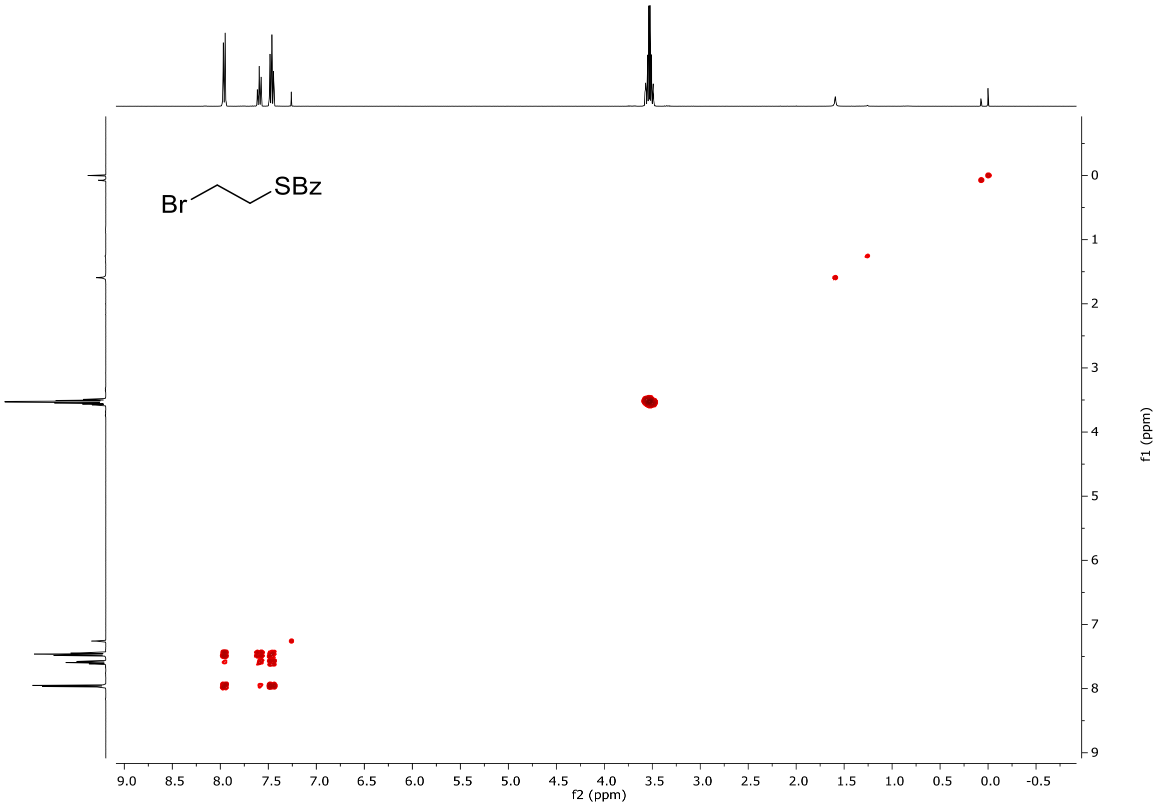
Br
SBz
f2 (ppm)
f1 (ppm)

Methyl 4-[(2-benzoylthioethyl)amino]-4-deoxy-2,3-*O*-(2’,3’-dimethoxybutane-2’,3’-diyl)-α-L-fucopyranoside (25)

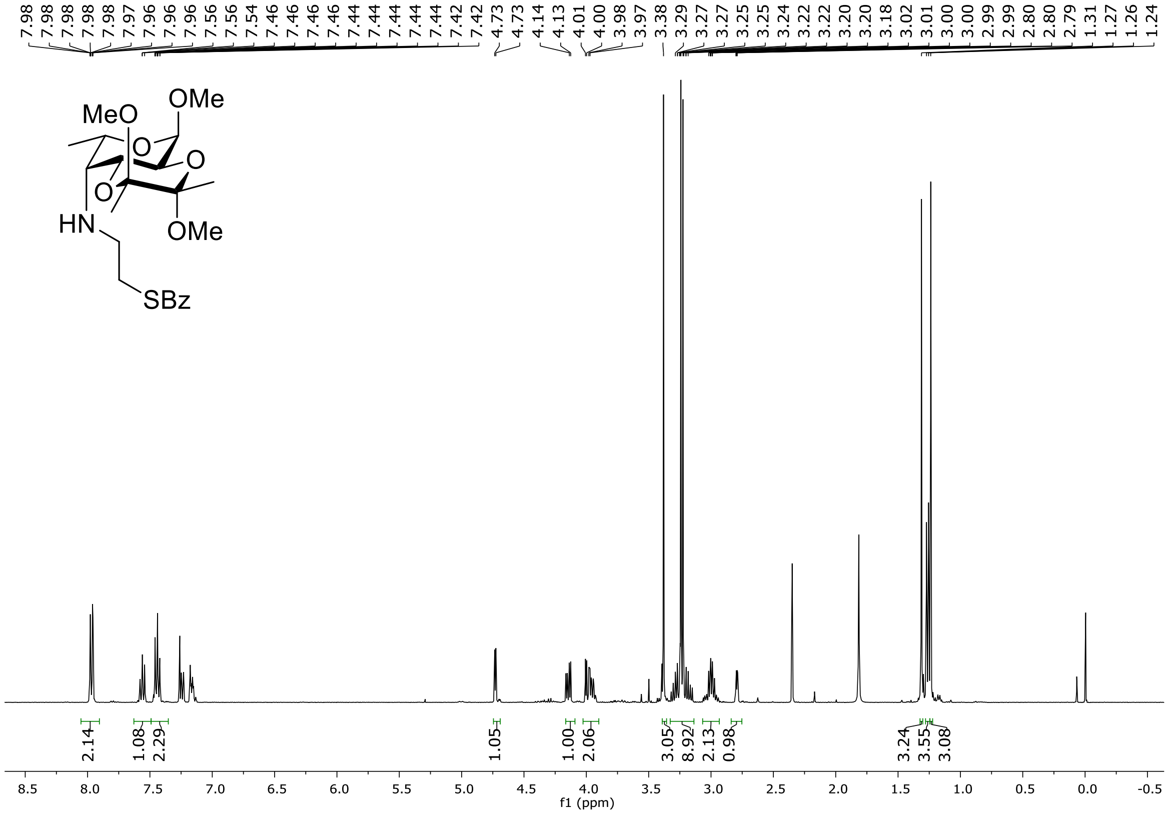

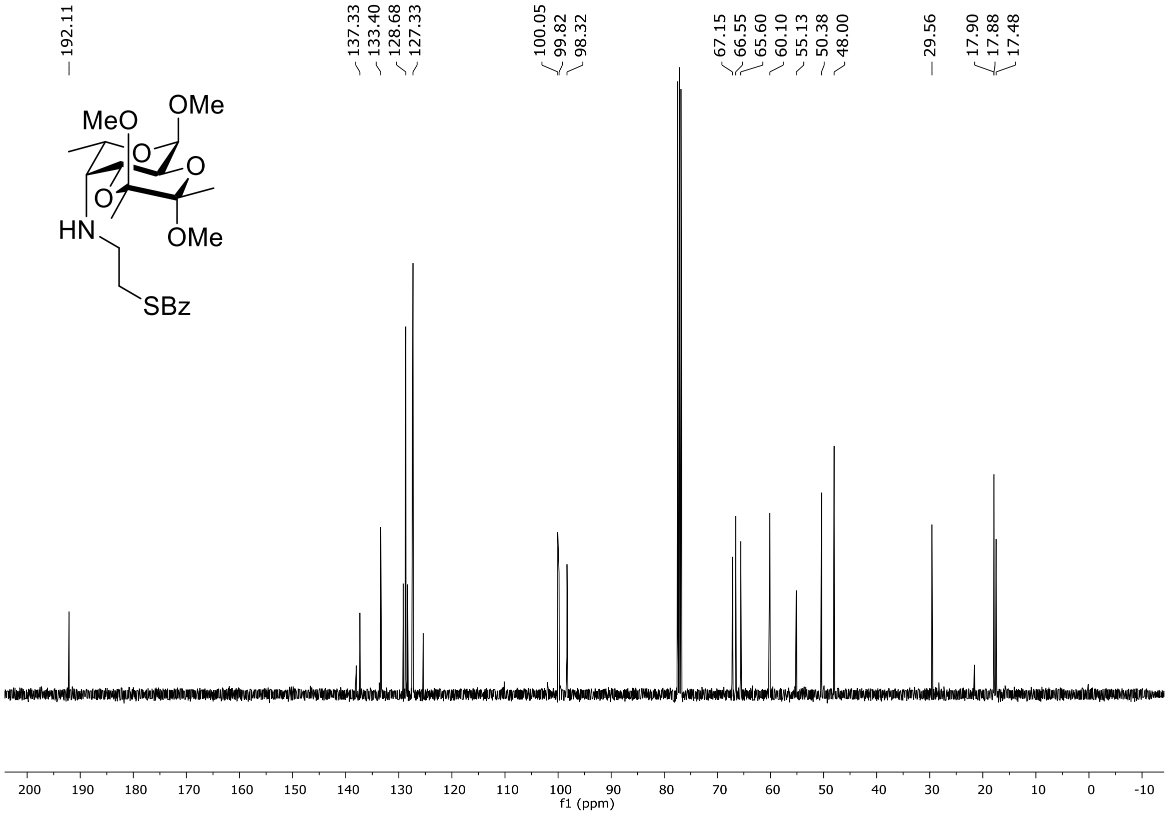

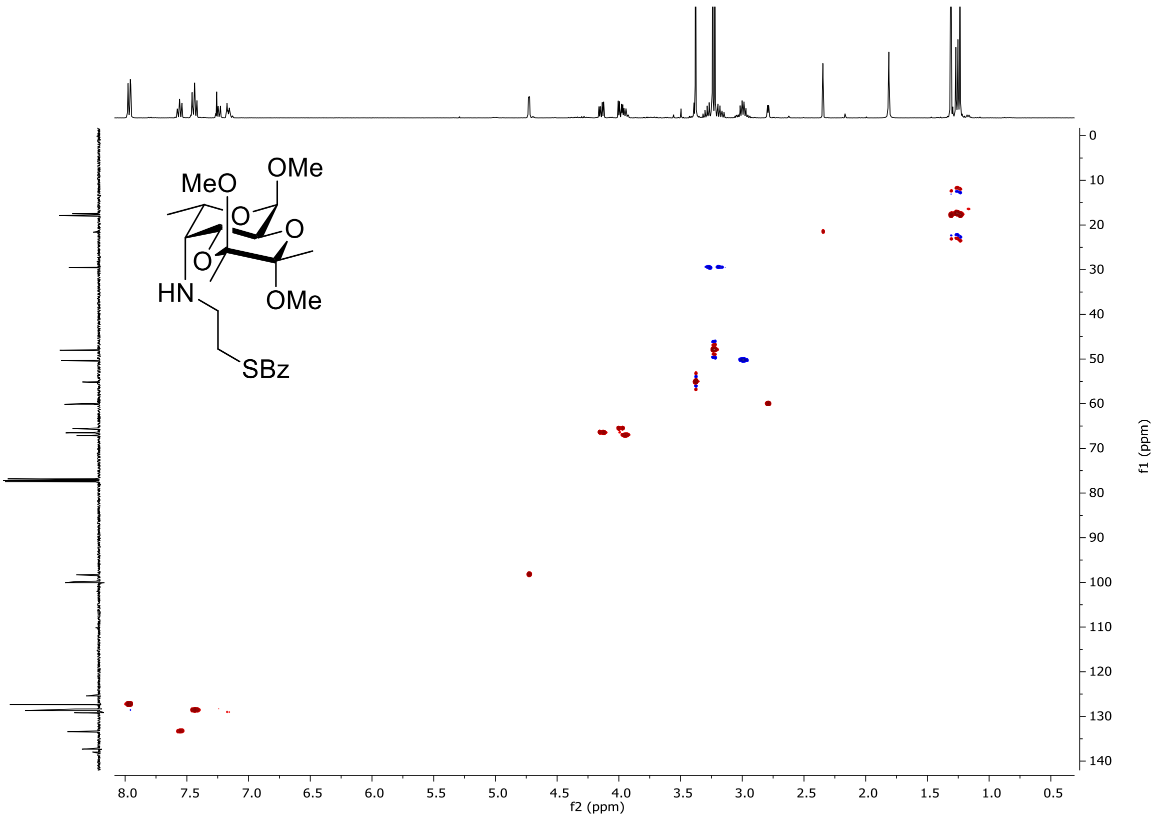
MeO
OMe
O
O
O
HN
OMe
SBz
f2 (ppm)
f1 (ppm)

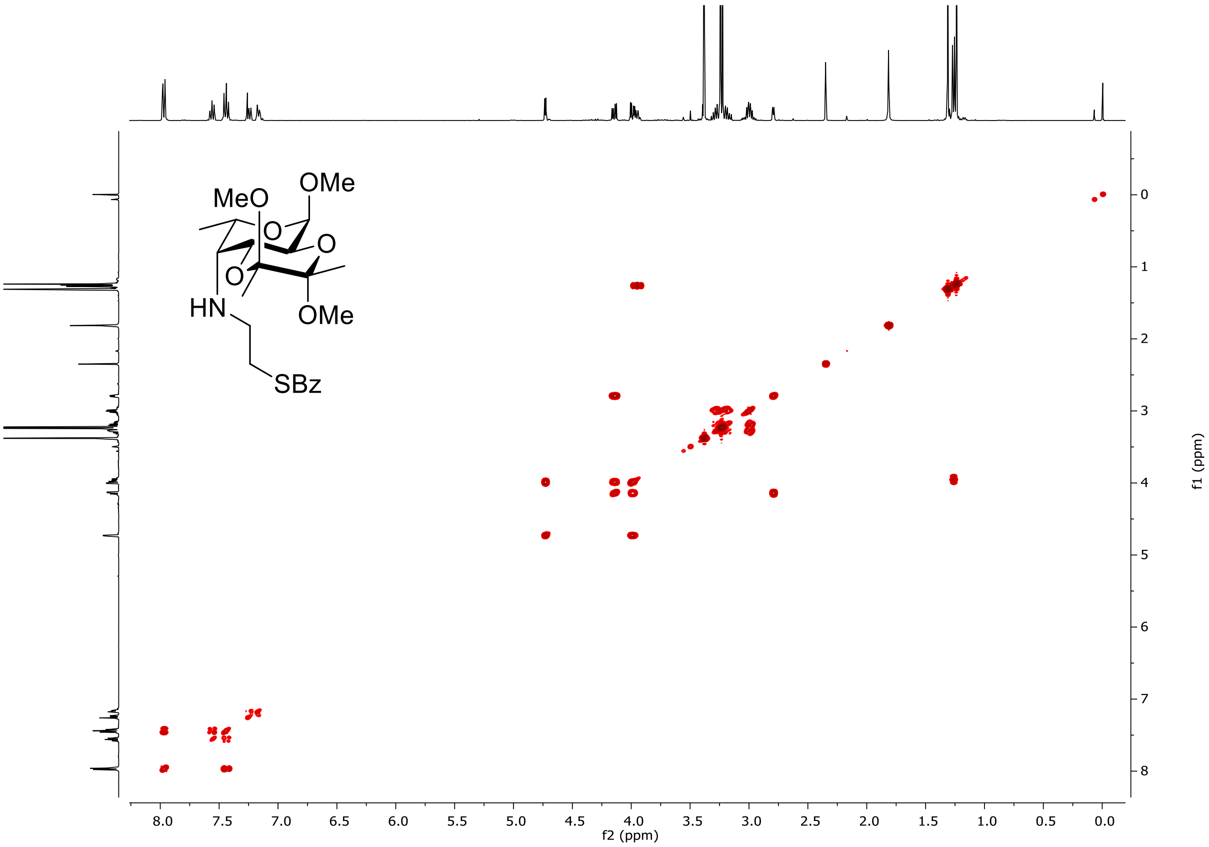
MeO
OMe
O
O
O
HN
OMe
SBz
f2 (ppm)
f1 (ppm)

Methyl 4-[(2-benzoylthioethyl)amino]-4-deoxy-α-L-fucopyranoside (26)

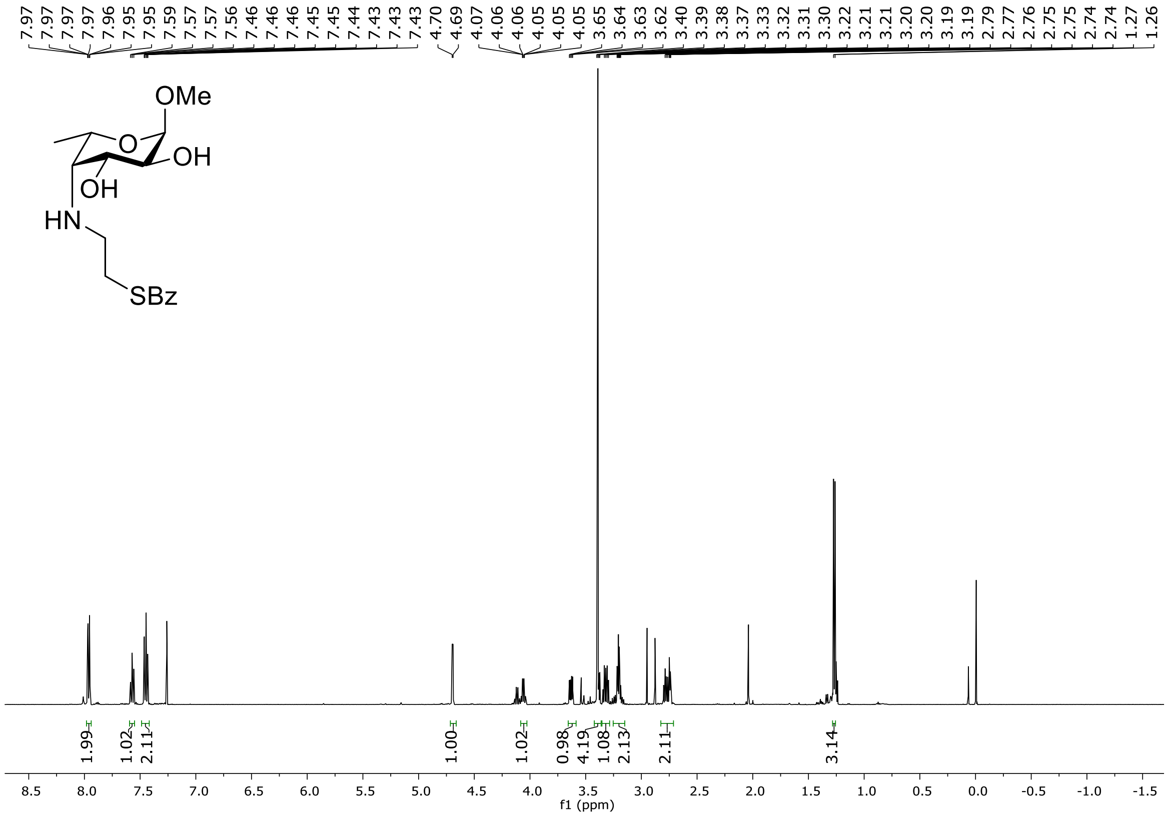

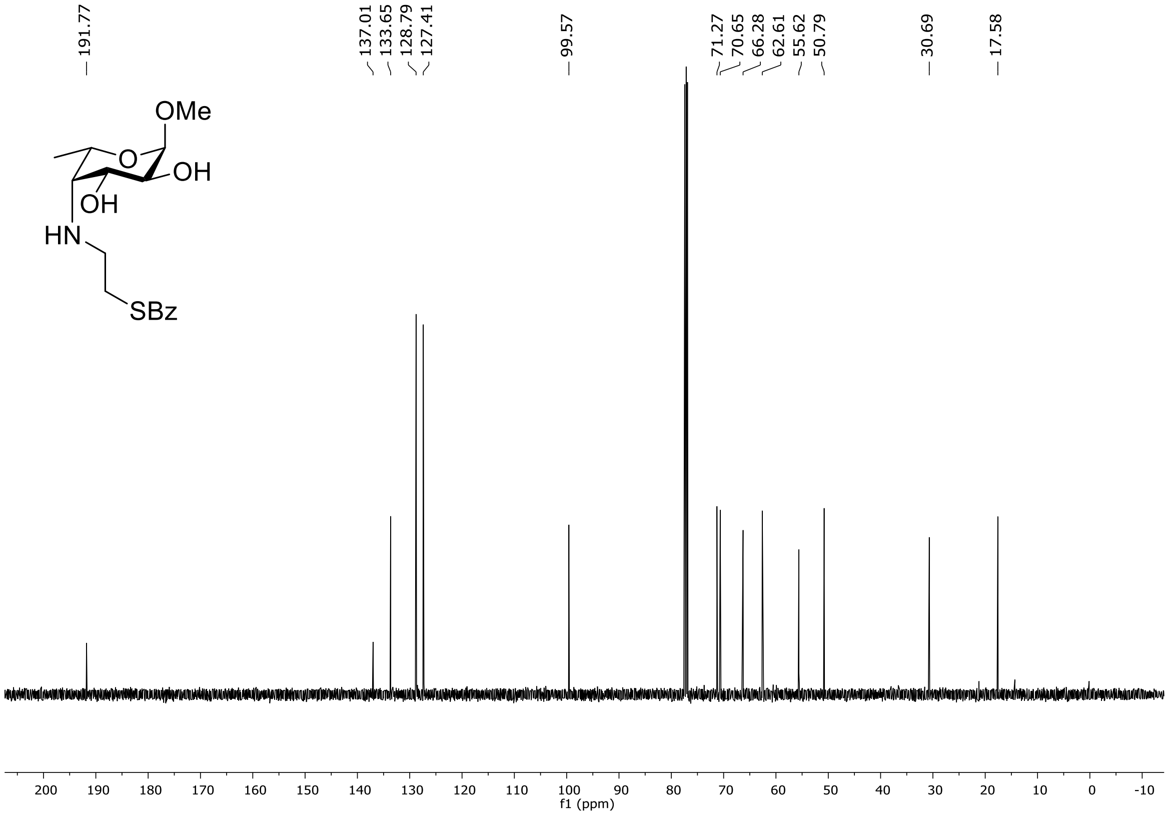

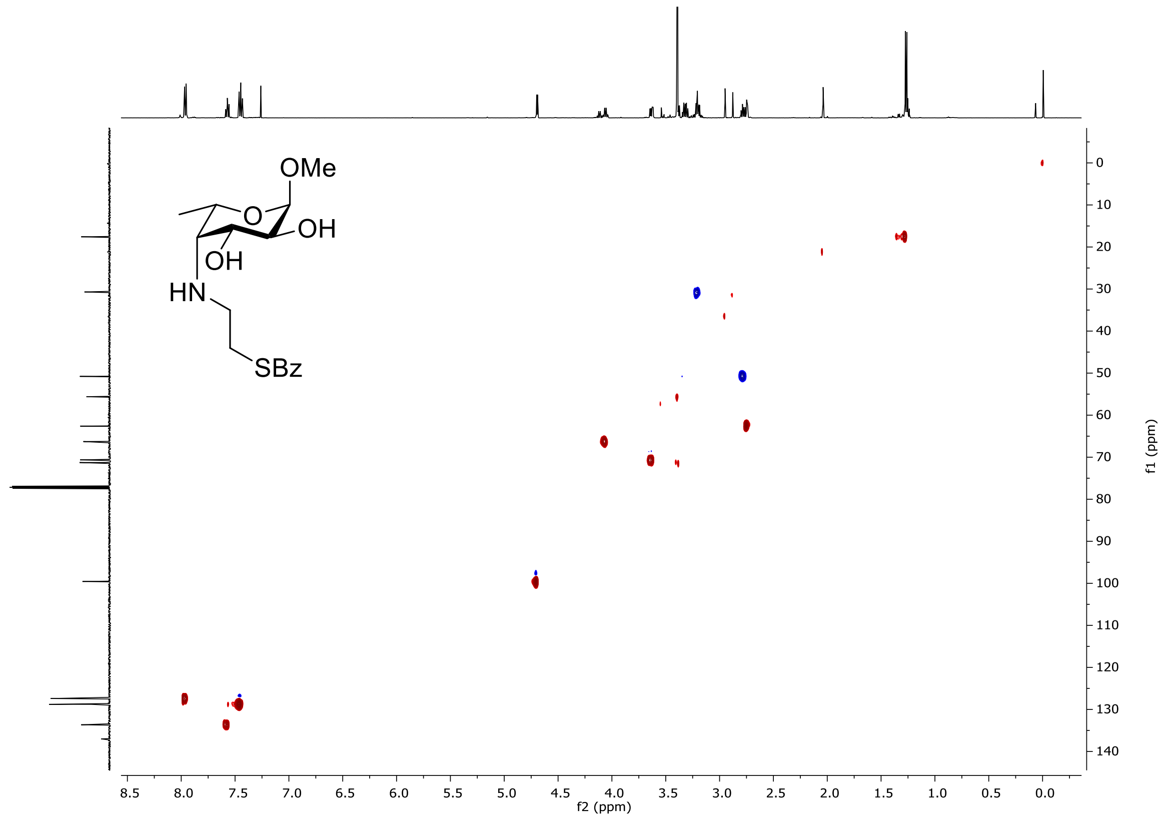
OMe
O
OH
OH
HN
SBz
f1 (ppm)
f2 (ppm)

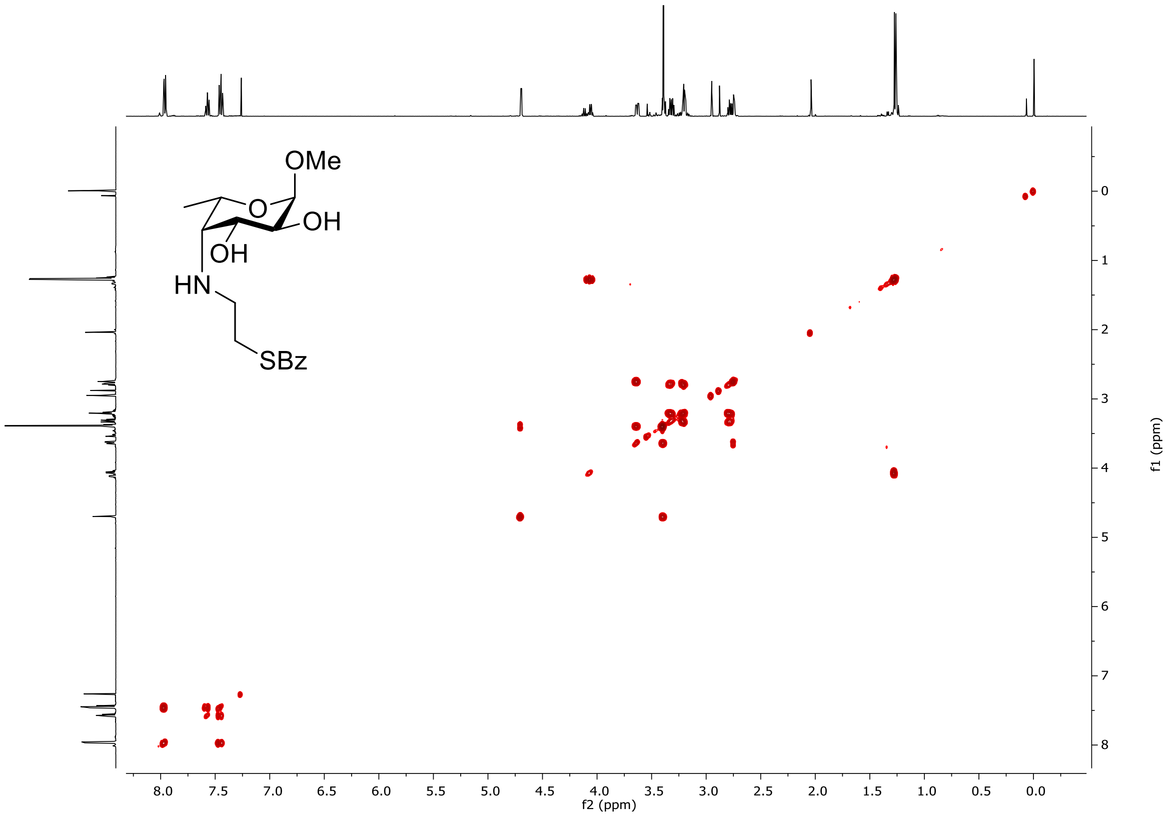
OMe
O
OH
OH
HN
SBz
f1 (ppm)
f2 (ppm)

Methyl 4-deoxy-4-(2-thioethyl)amino-α-L-fucopyranoside (5)

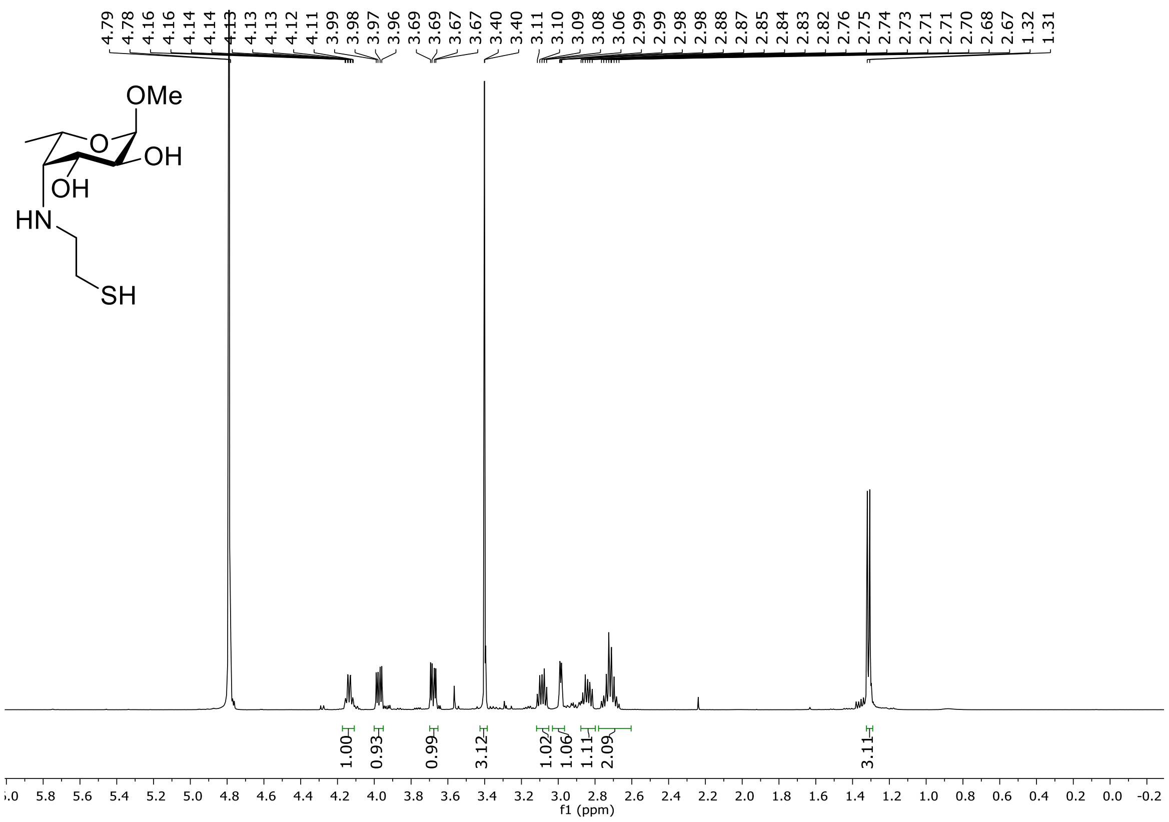

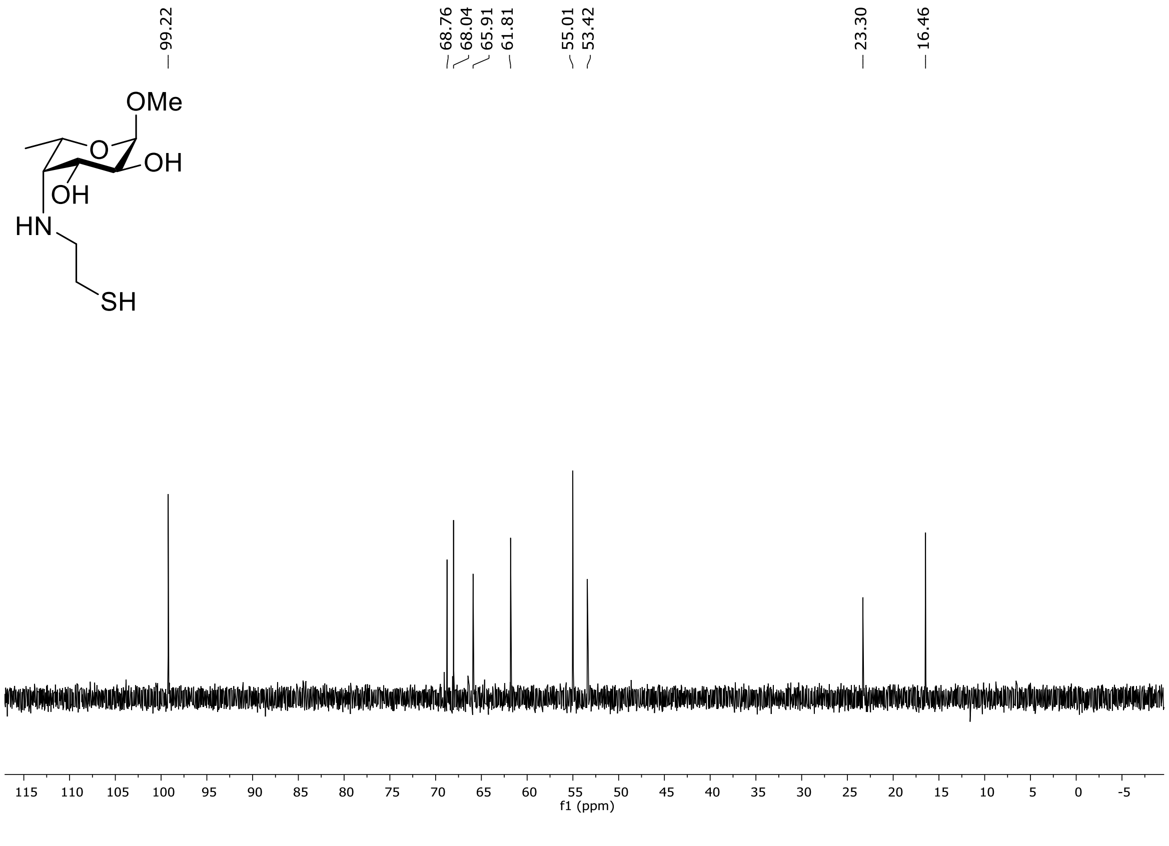

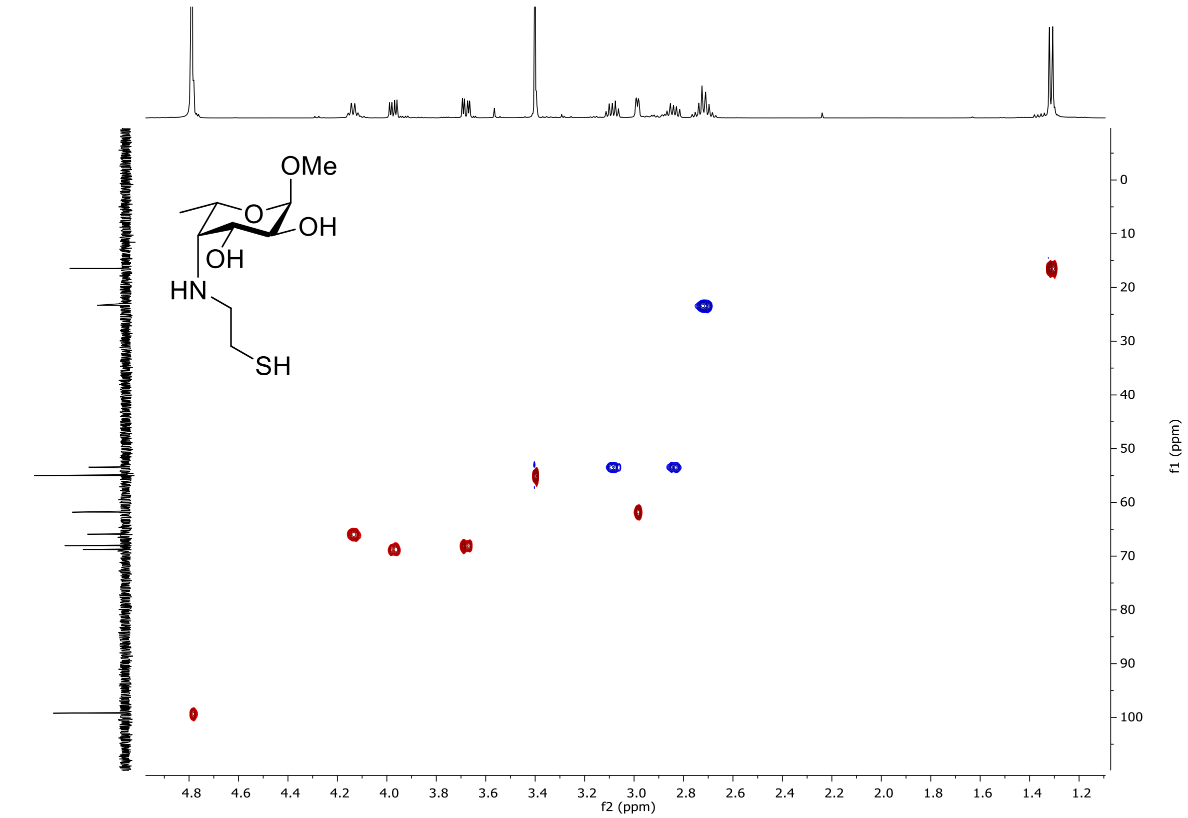
OMe
OH
OH
HN
SH
f1 (ppm)
f2 (ppm)

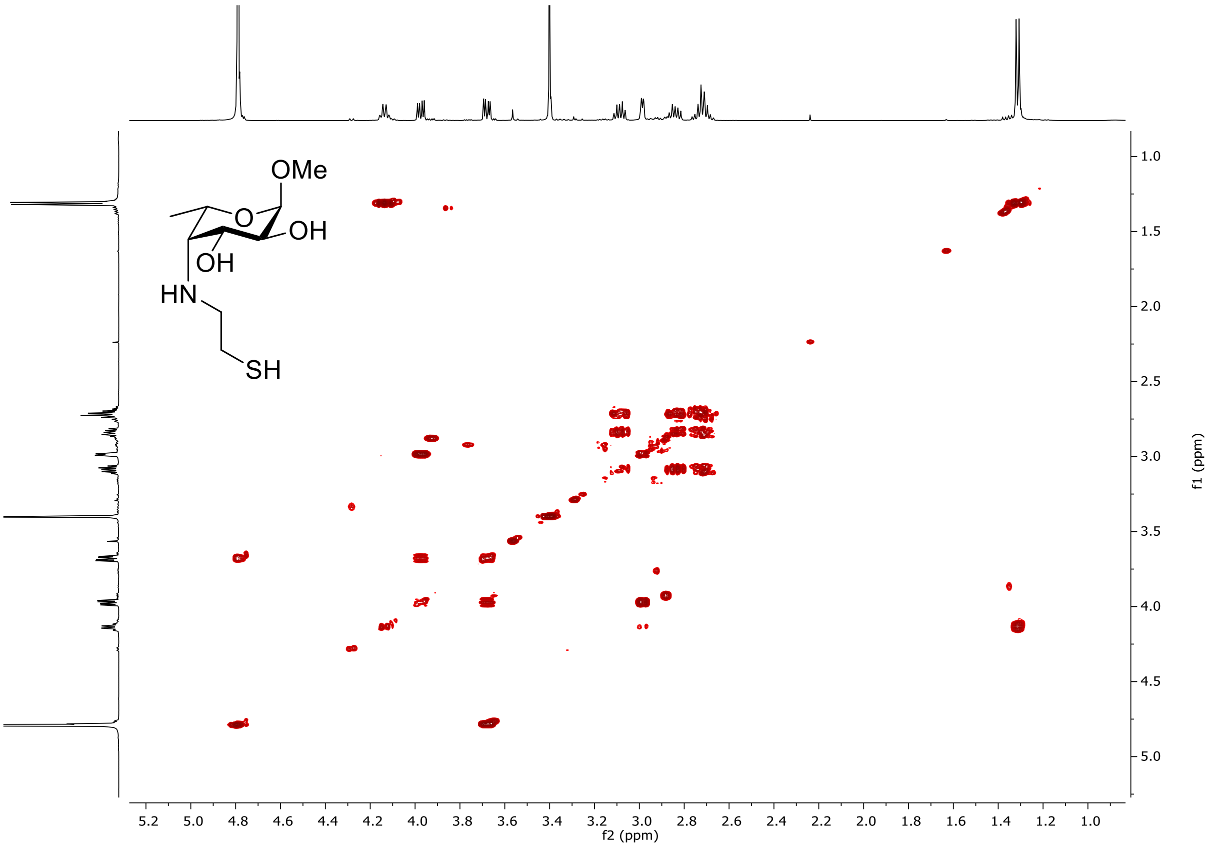
OMe
OH
OH
HN
SH
f1 (ppm)
f2 (ppm)

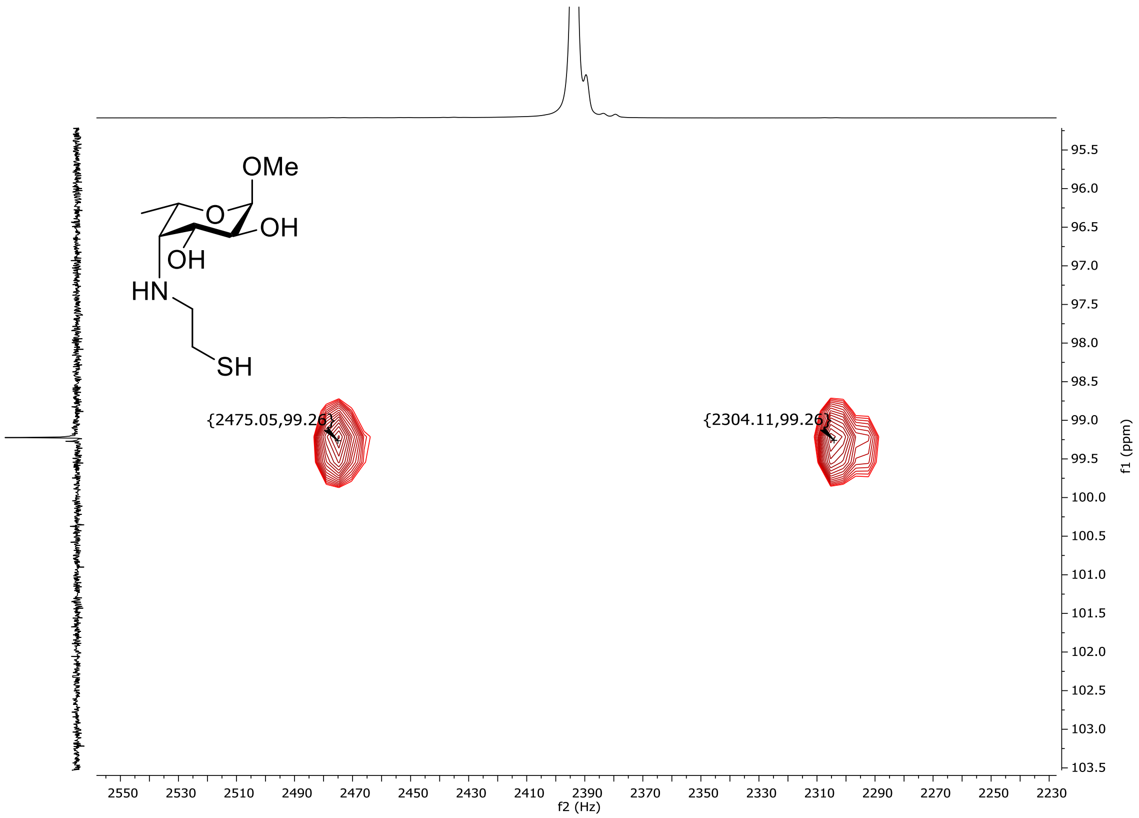
OMe
O
OH
OH
HN
SH
{2475.05,99.26}
{2304.11,99.26}
f1 (ppm)
f2 (Hz)

Methyl 4-allylamino-4-deoxy-2,3-*O*-(2’,3’-dimethoxybutane-2’,3’-diyl)-α-L-fucopyranoside (27)

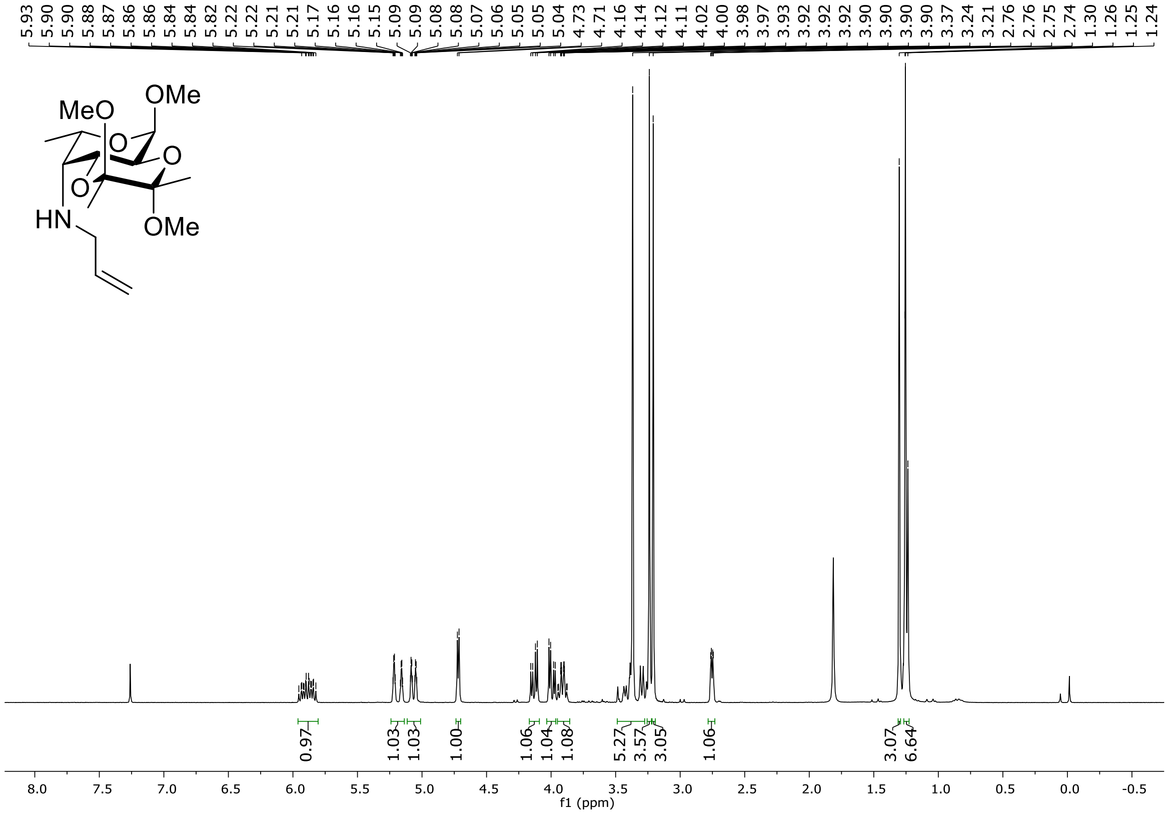

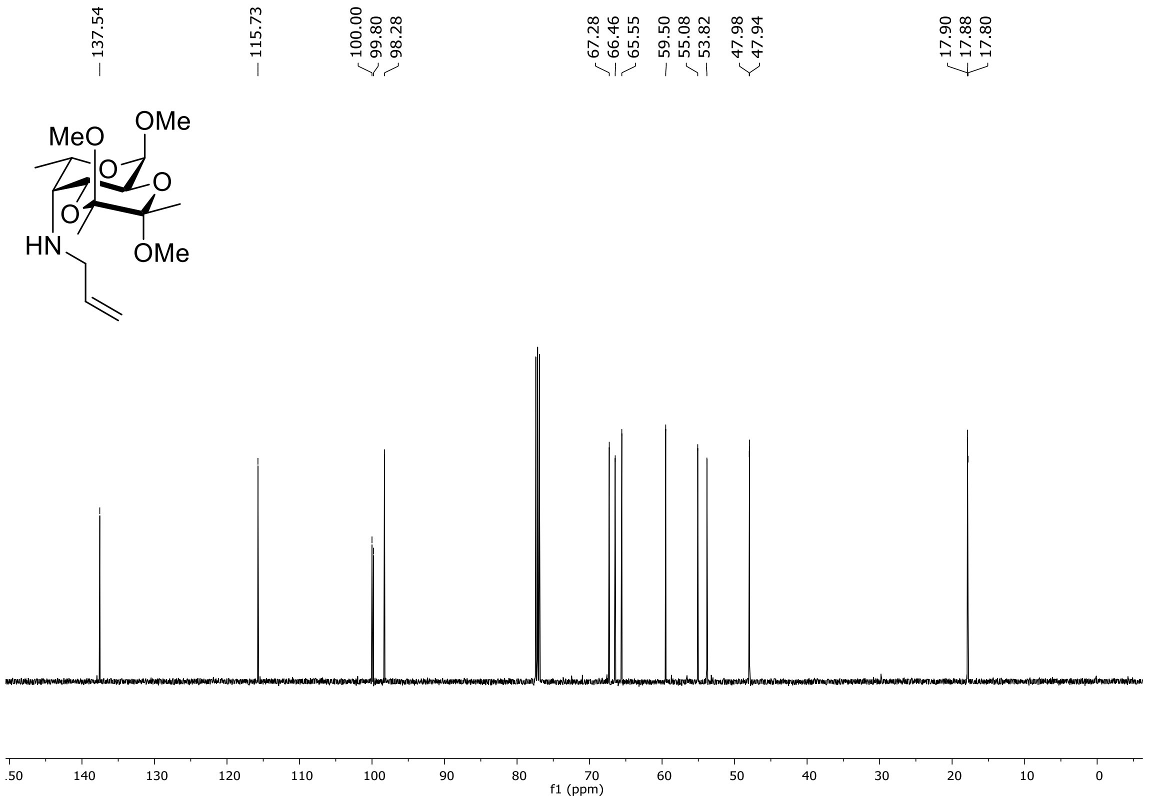

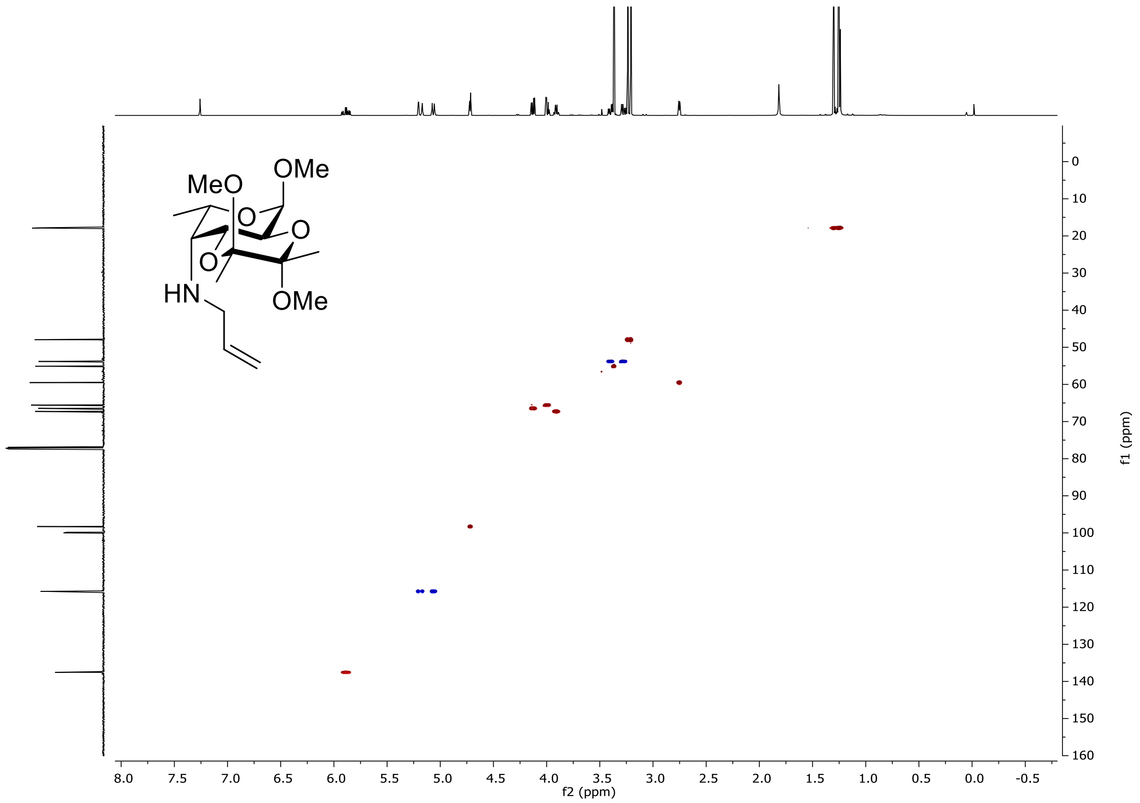
MeO
OMe
O
O
O
HN
OMe
f2 (ppm)
f1 (ppm)

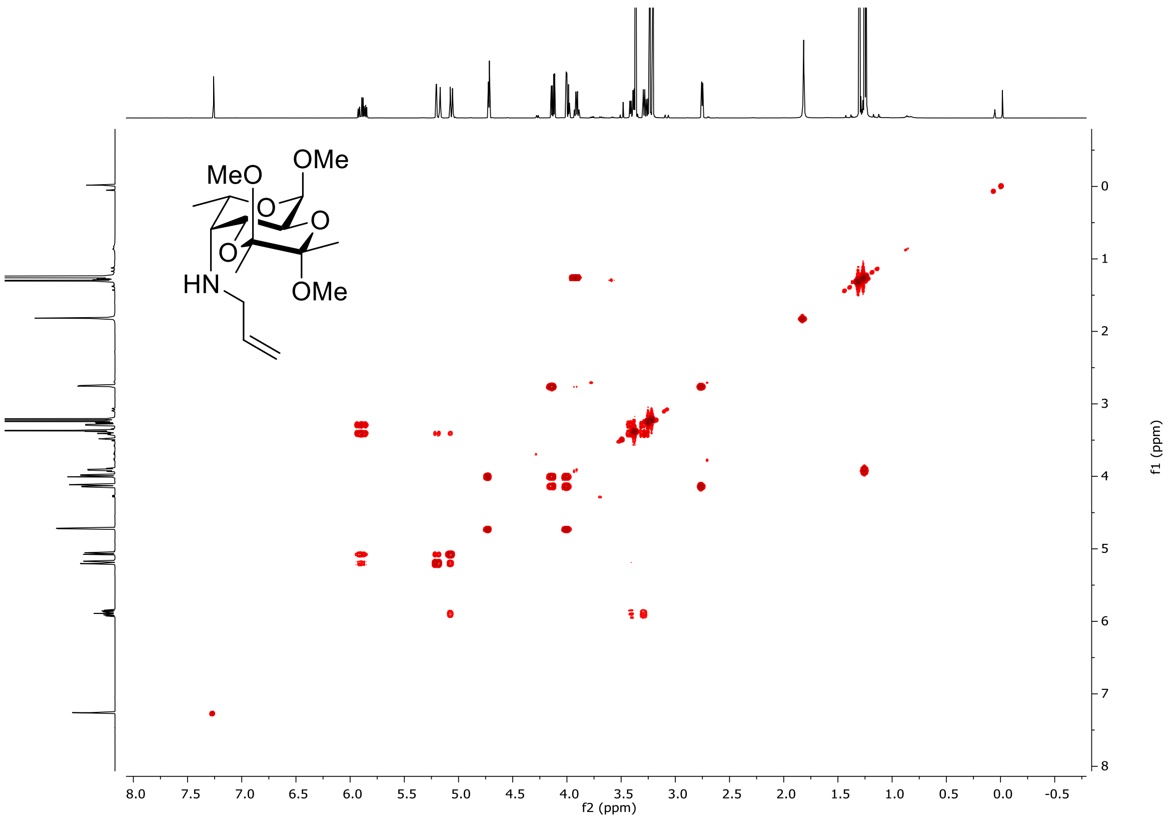
MeO
OMe
O
O
O
HN
OMe
f2 (ppm)
f1 (ppm)

Methyl 4-[(3-acetylthiopropyl)amino]-4-deoxy-2,3-*O*-(2’,3’-dimethoxybutane-2’,3’-diyl)-α-L-fucopyranoside (28)

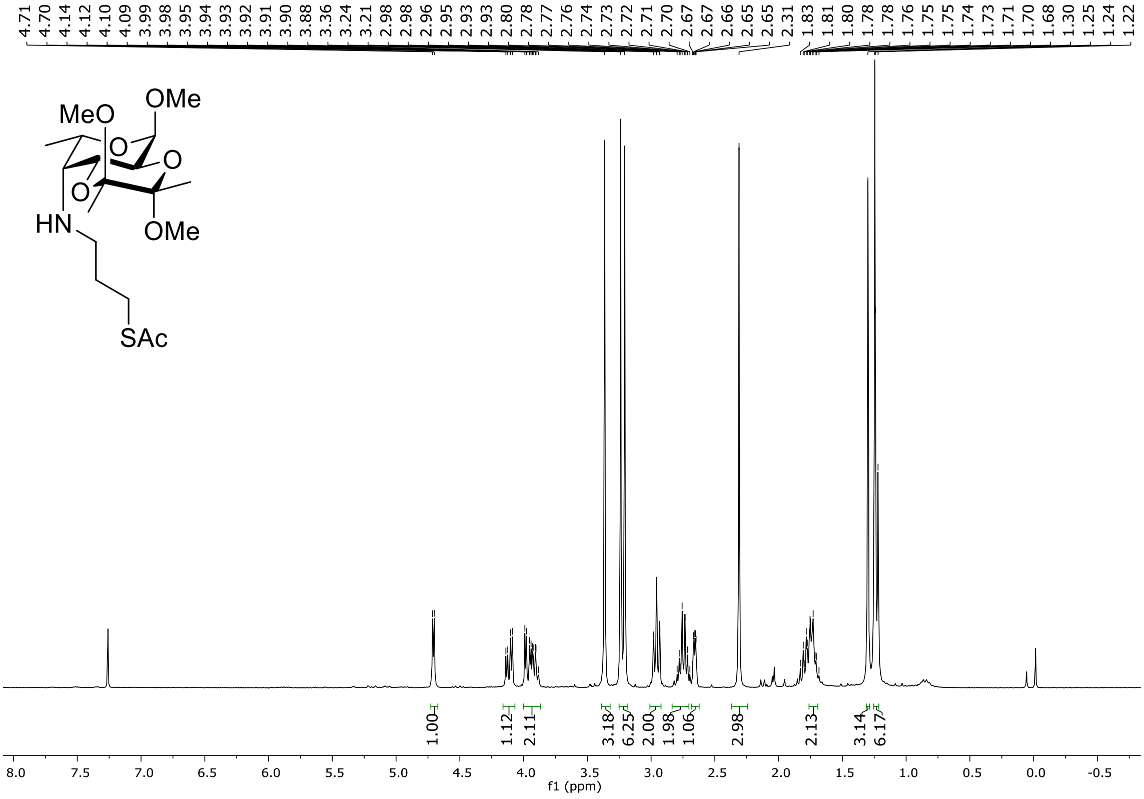

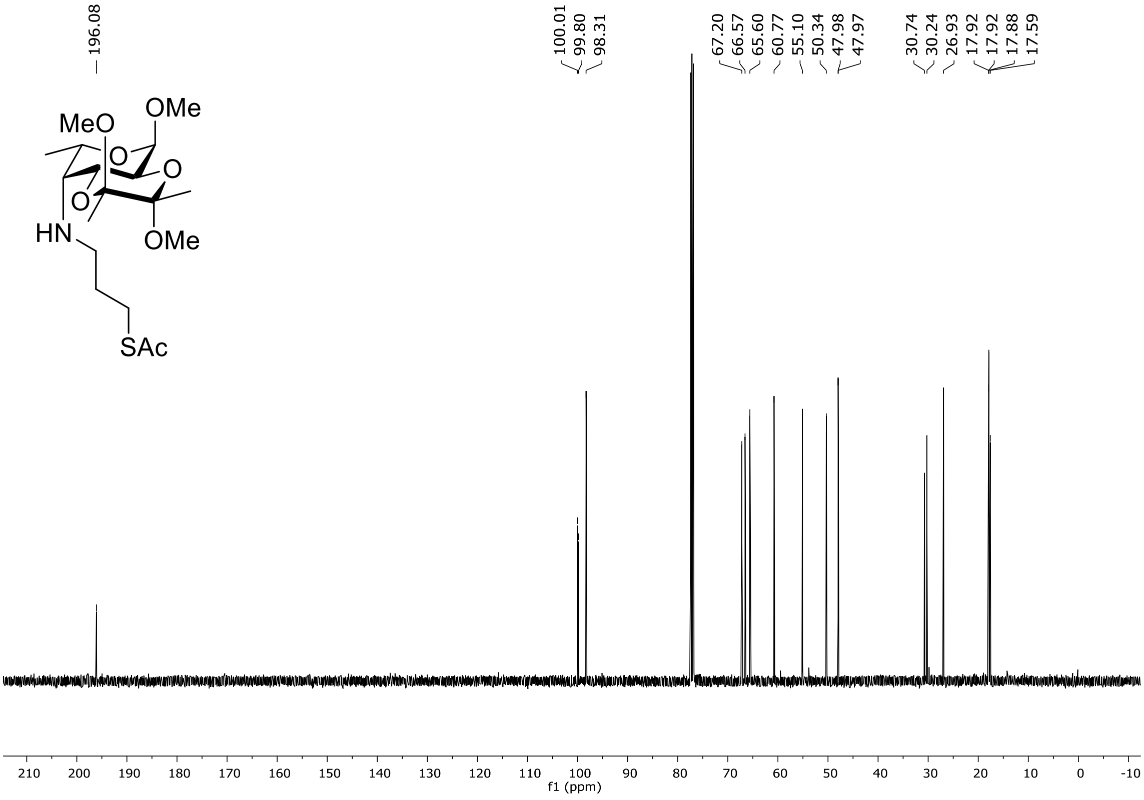

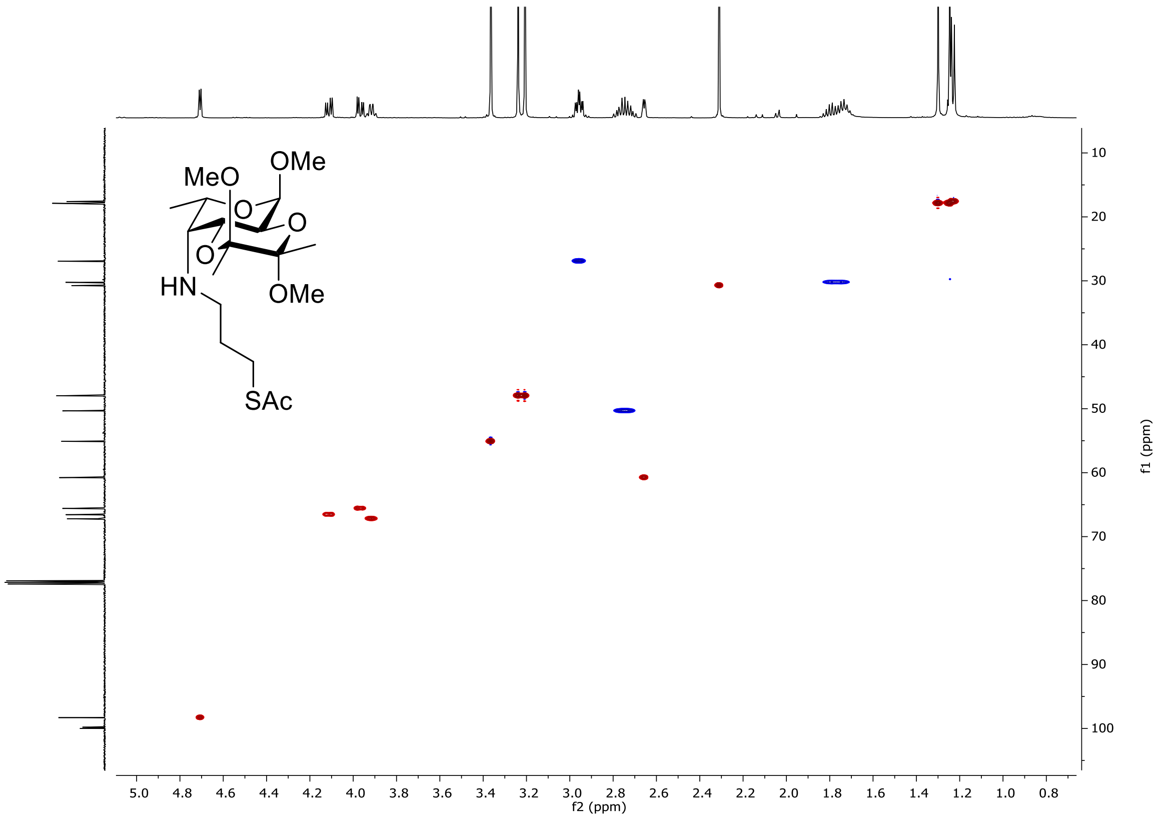
MeO
OMe
O
O
O
HN
OMe
SAc
f2 (ppm)
f1 (ppm)

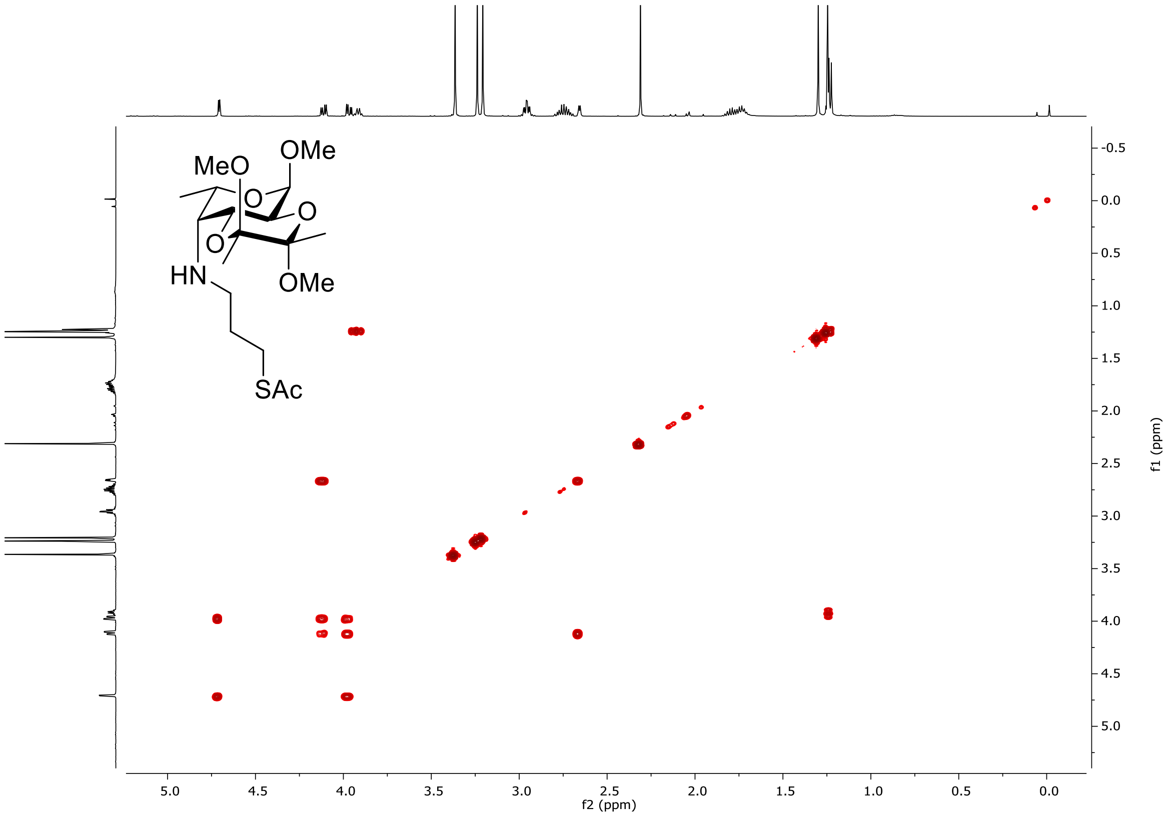
MeO
OMe
O
O
O
HN
OMe
SAc
f2 (ppm)
f1 (ppm)

Methyl 4-[(3-acetylthiopropyl)amino]-4-deoxy-α-L-fucopyranoside (29)

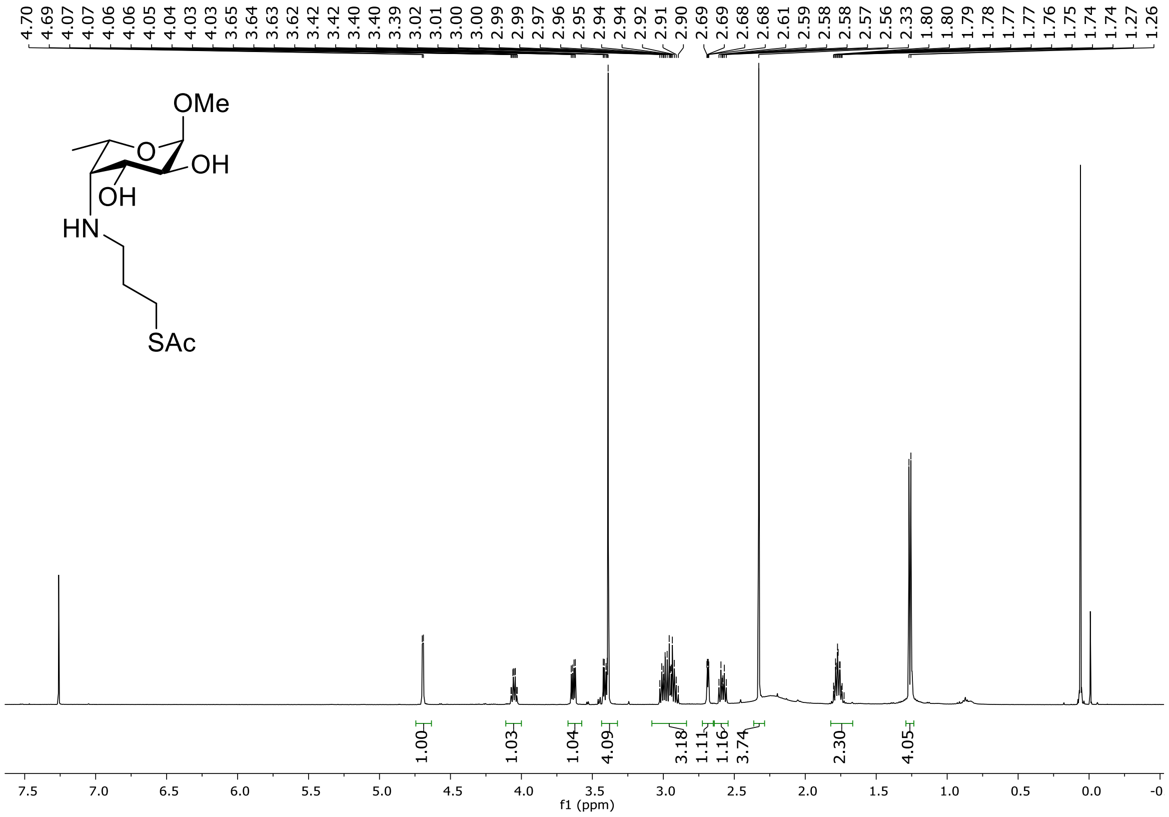

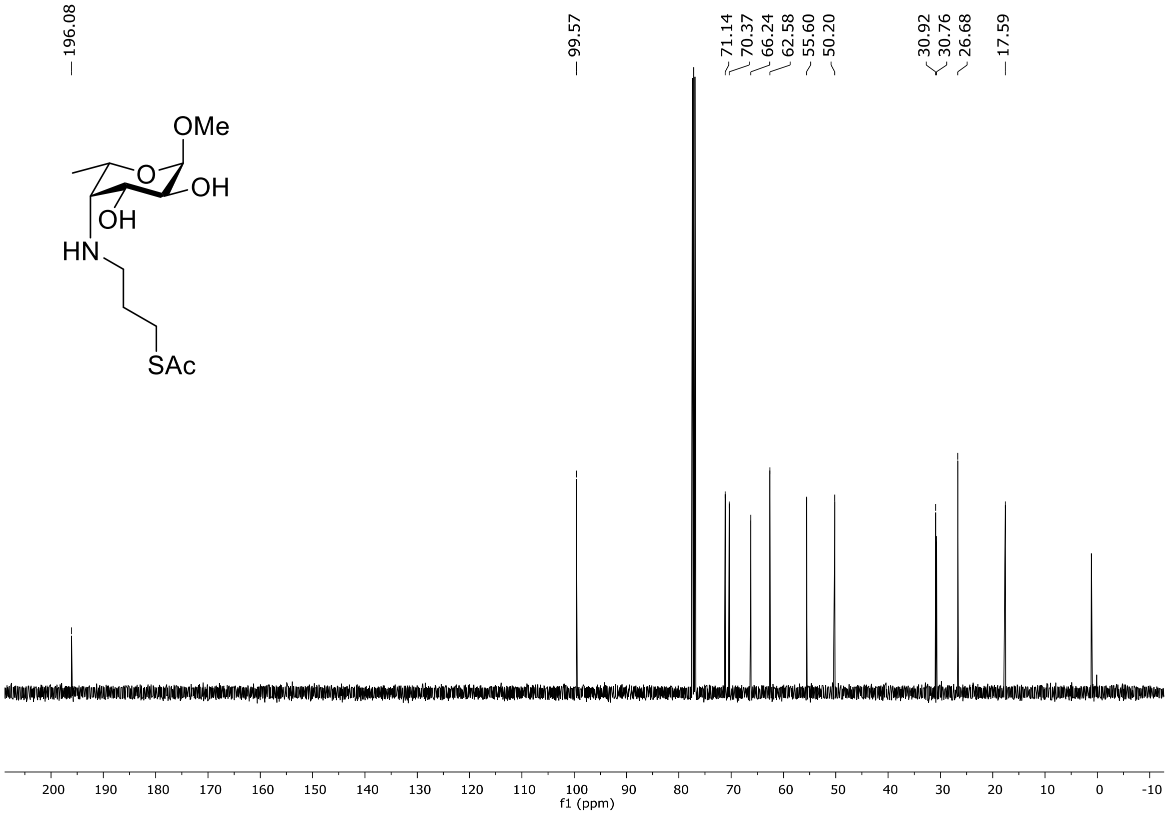

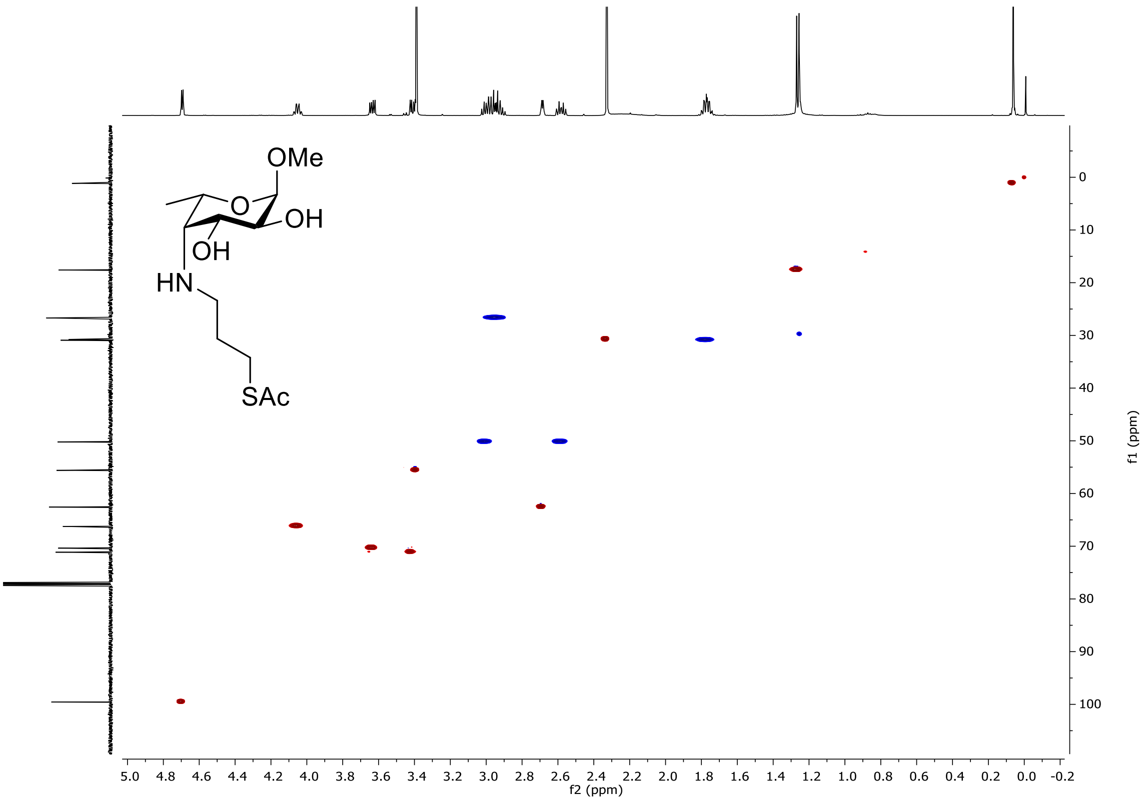
OMe
O
OH
OH
HN
SAc
f1 (ppm)
f2 (ppm)

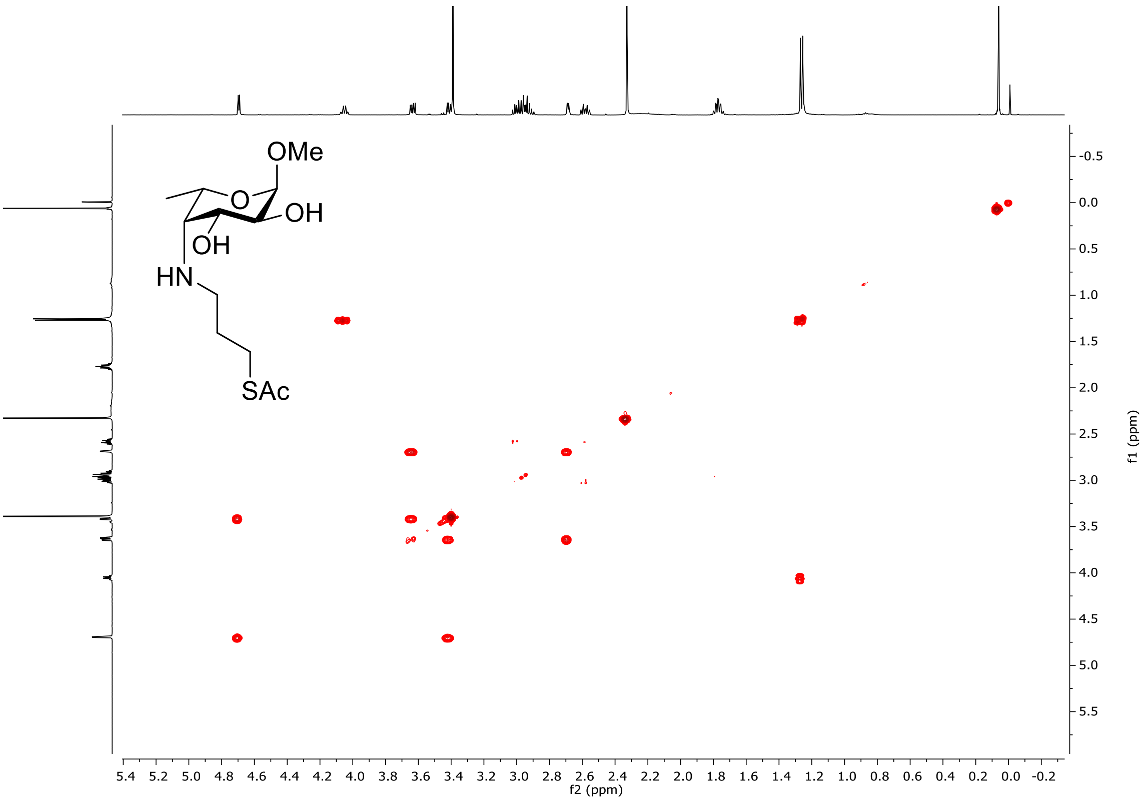
OMe
O
OH
OH
HN
SAc
f1 (ppm)
f2 (ppm)

Methyl 4-deoxy-4-[(3-thiopropyl)amino]-α-L-fucopyranoside (6)

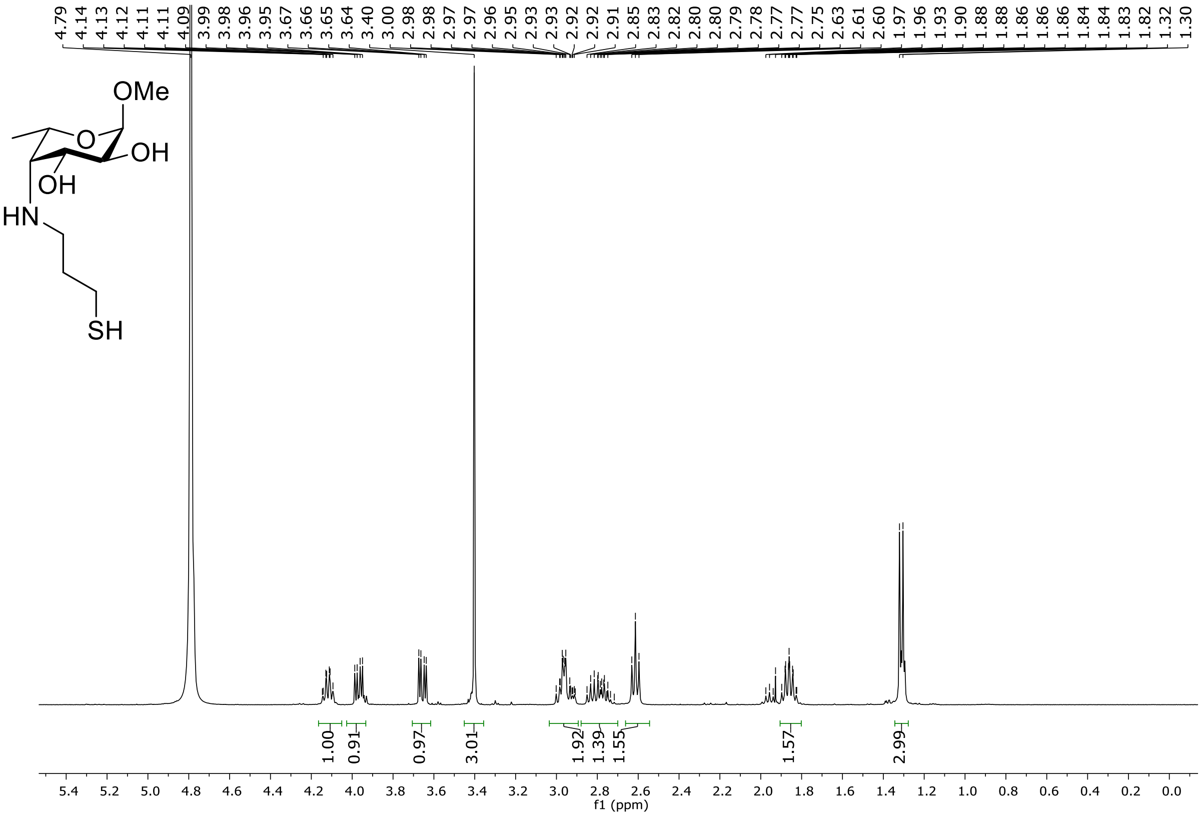

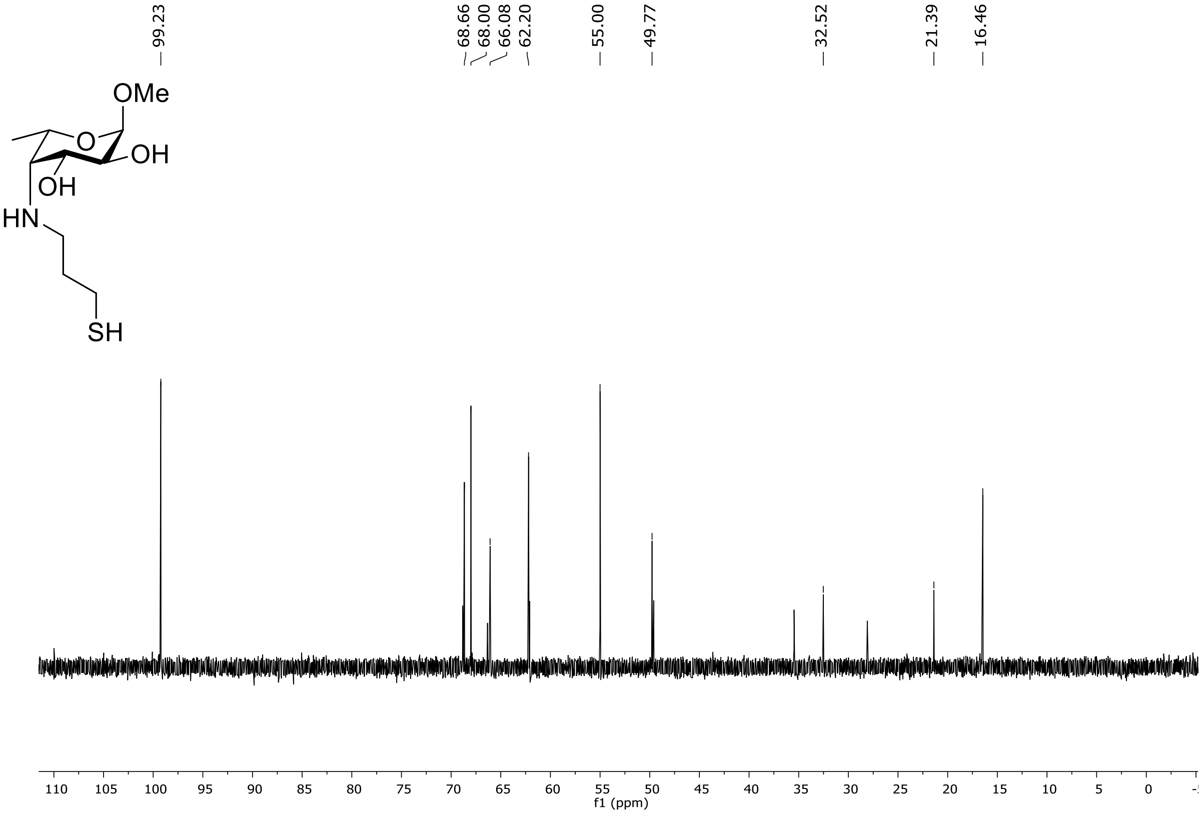

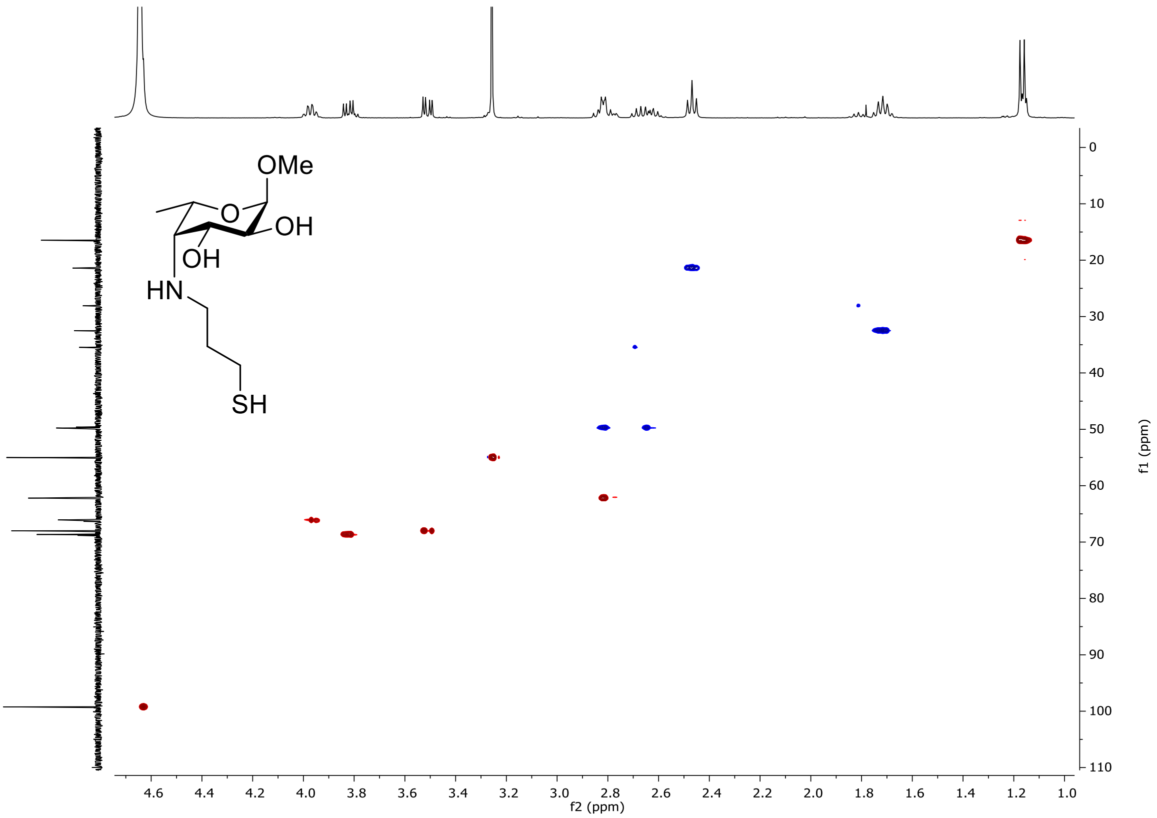
OMe
O
OH
OH
HN
SH
f1 (ppm)
f2 (ppm)

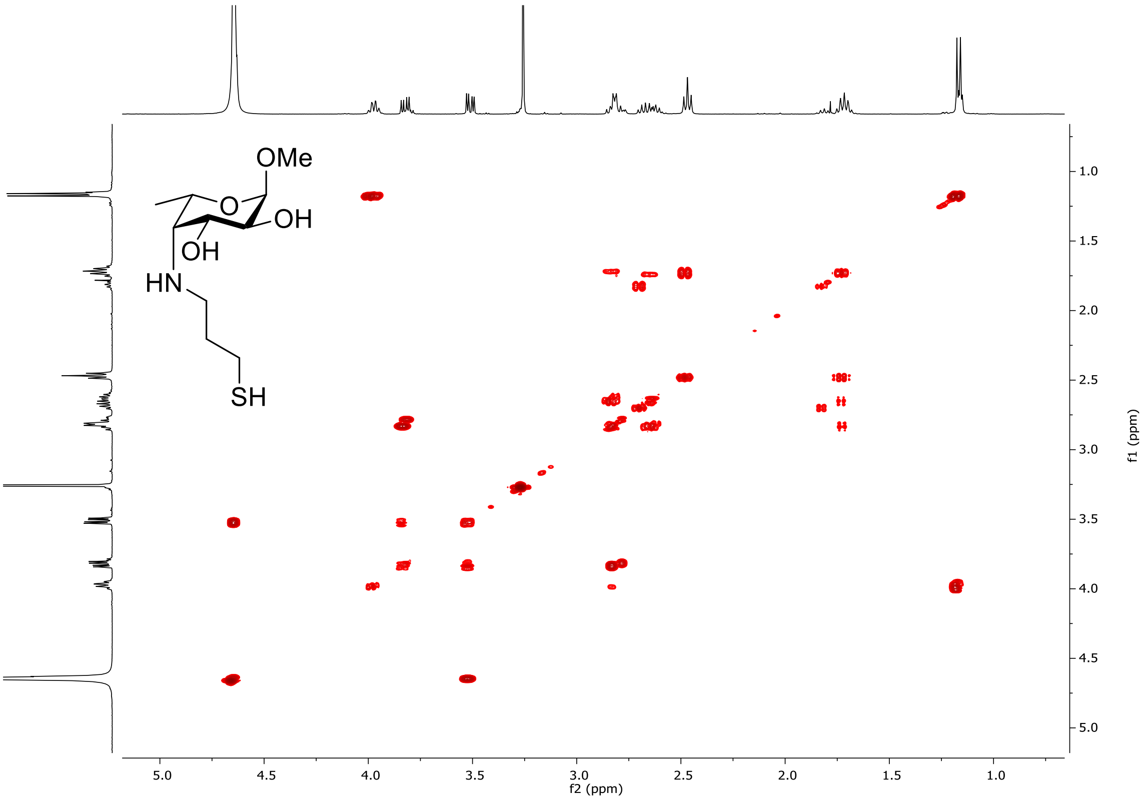
OMe
O
OH
OH
HN
SH
f1 (ppm)
f2 (ppm)

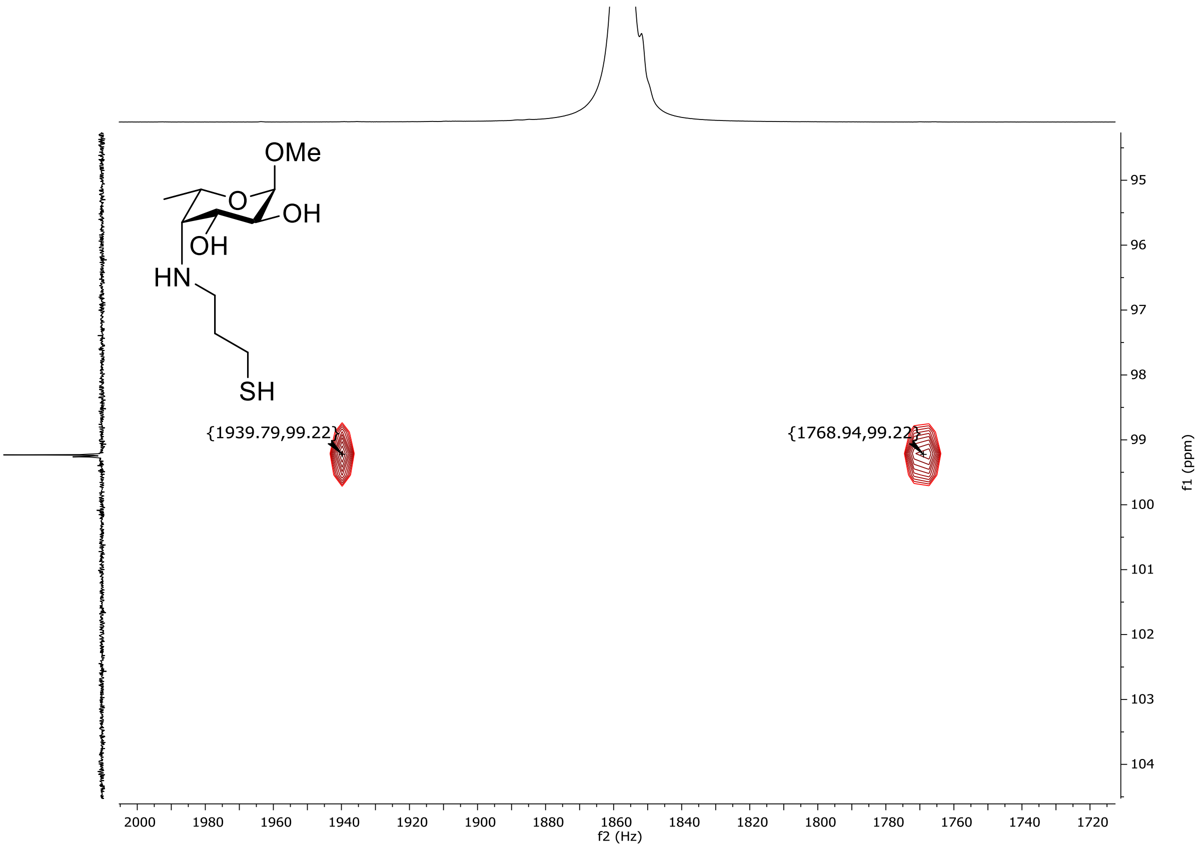
OMe
O
OH
OH
HN
SH
{1939.79,99.22}
{1768.94,99.22}
f1 (ppm)
f2 (Hz)
